# Supplementary material for: PARP7 inhibitors enhance the immunogenic effects of radiation in pancreatic cancer cells
Source: Mol Ther Oncol. 2026 Jun 8;34(3):201258. doi: 10.1016/j.omton.2026.201258 (PMC13316578; doi:10.1016/j.omton.2026.201258)
Supplement: Document S1. Figures S1–S13, Tables S3, and S4 [file mmc1.pdf]

OMTON, Volume *34*

Supplemental information

PARP7 inhibitors enhance the immunogenic effects of radiation in pancreatic cancer cells

Niccolò Bragato, Ana Beatriz Dias, Anna Ohradanova-Repic, Alma Dupanovic, Filip Horvat, Patrick Fischer, Lisa-Marie Appel, Lena Walch, Ava Kleinwächter, Anna Röhrer, Sylvia Kerschbaum-Gruber, Sandra Barna, Piero Fossati, Dietmar Georg, Joachim Widder, Klaus Podar, Michael Cohen, and Dea Slade

Supplemental Figures

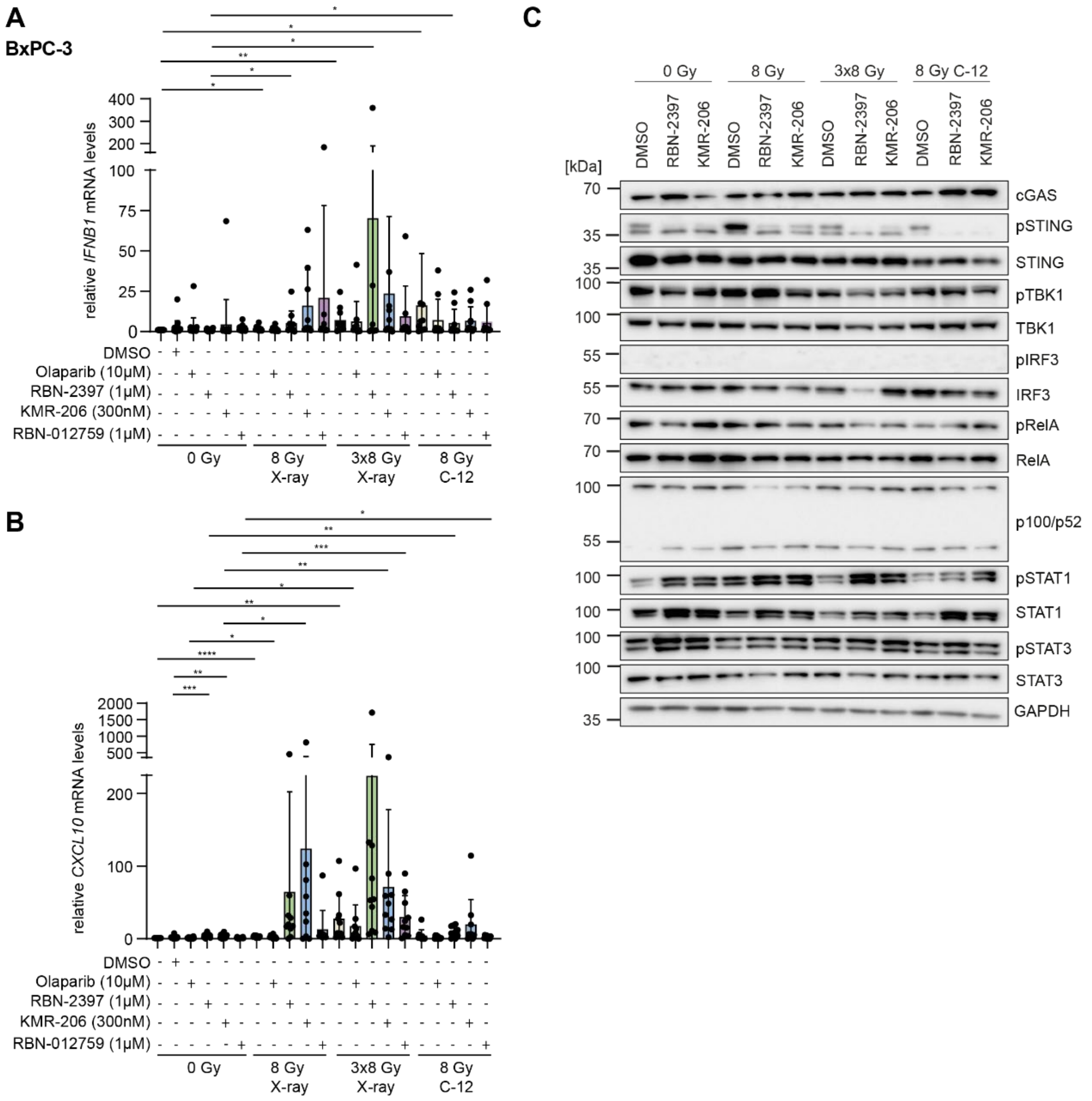

Fig. S1. PARP7 inhibitors potentiate the immunogenic effects of hypofractionated X-ray irradiation in BxPC-3 cells. (A, B) RT-qPCR analysis of the expression of *IFNB1* and *CXCL10* in BxPC-3 cells after 8 Gy or 3x8 Gy X-ray and 8 Gy C-ions without or with the PARP1/2 inhibitor olaparib, PARP7 inhibitors RBN-2397 and KMR-206 and the PARP14 inhibitor RBN-012759. Inhibitors were added 24 h before irradiation and kept until harvesting 72 h after the last fraction. Gene expression was normalized to TBP. Data points represent mean values ± standard deviation (N=3-6). Multiple t-tests were performed to determine significance (*≤ 0.05; **≤ 0.01; ***≤ 0.005, ****≤0.0001). (C) Western blot analysis of BxPC-3 cells irradiated with 8 Gy or 3x8 Gy X-ray and 8 Gy C-ions without or with PARP7 inhibitors RBN-2397 and KMR-206. GAPDH was used as a loading control.

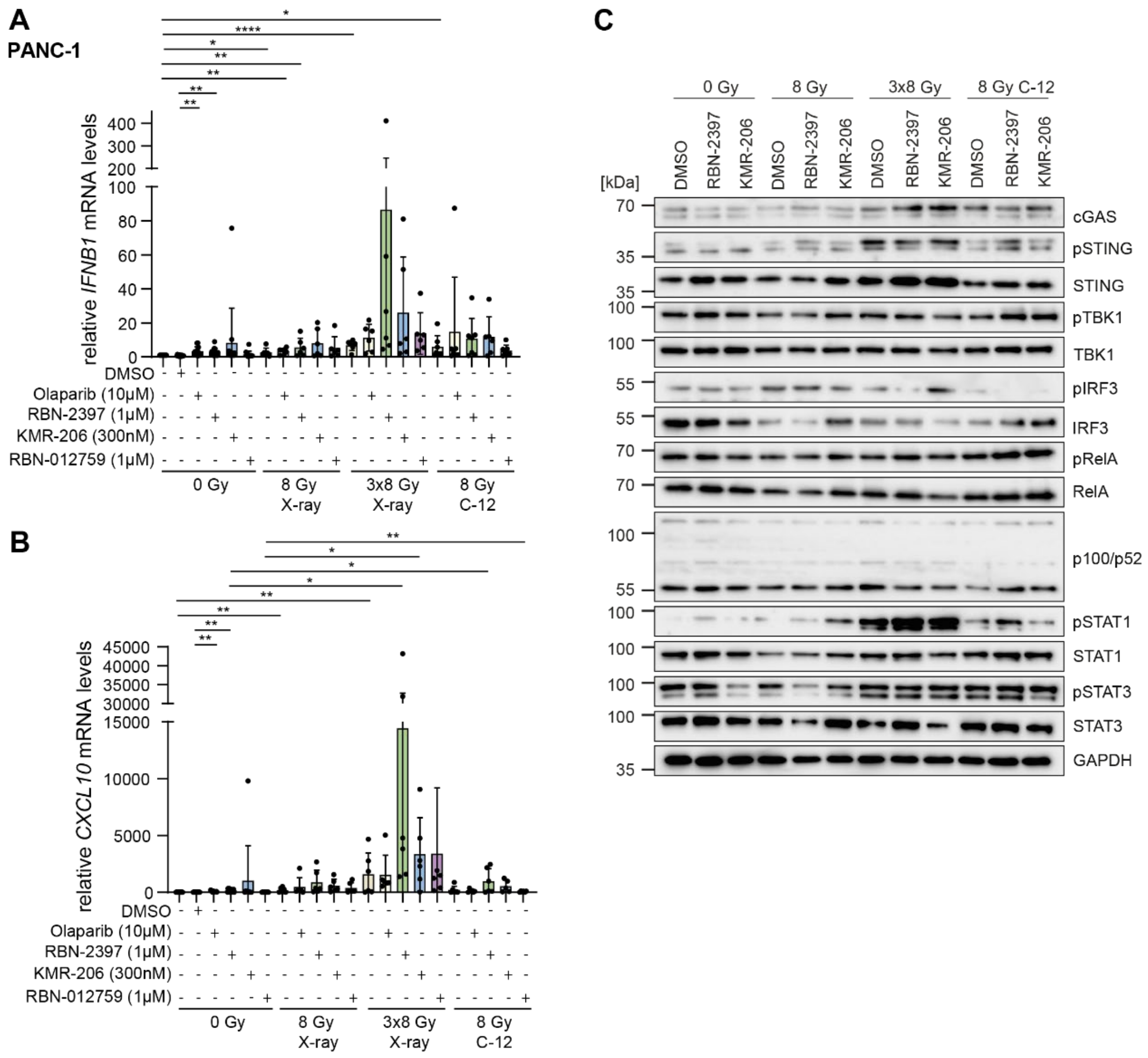

Fig. S2. PARP7 inhibitors potentiate the immunogenic effects of hypofractionated X-ray irradiation in PANC-1 cells. (A, B) RT-qPCR analysis of the expression of *IFNB1* and *CXCL10* in PANC-1 cells after 8 Gy or 3x8 Gy X-ray and 8 Gy C-ions without or with the PARP1/2 inhibitor olaparib, PARP7 inhibitors RBN-2397 and KMR-206 and the PARP14 inhibitor RBN-012759. Inhibitors were added 24 h before irradiation and kept until harvesting 72 h after the last fraction. Gene expression was normalized to TBP. Data points represent mean values ± standard deviation (N=3-6). Multiple t-tests were performed to determine significance (*≤ 0.05; **≤ 0.01; ***≤ 0.005, ****≤0.0001). (C) Western blot analysis of PANC-1 cells irradiated with 8 Gy or 3x8 Gy X-ray and 8 Gy C-ions without or with PARP7 inhibitors RBN-2397 and KMR-206.

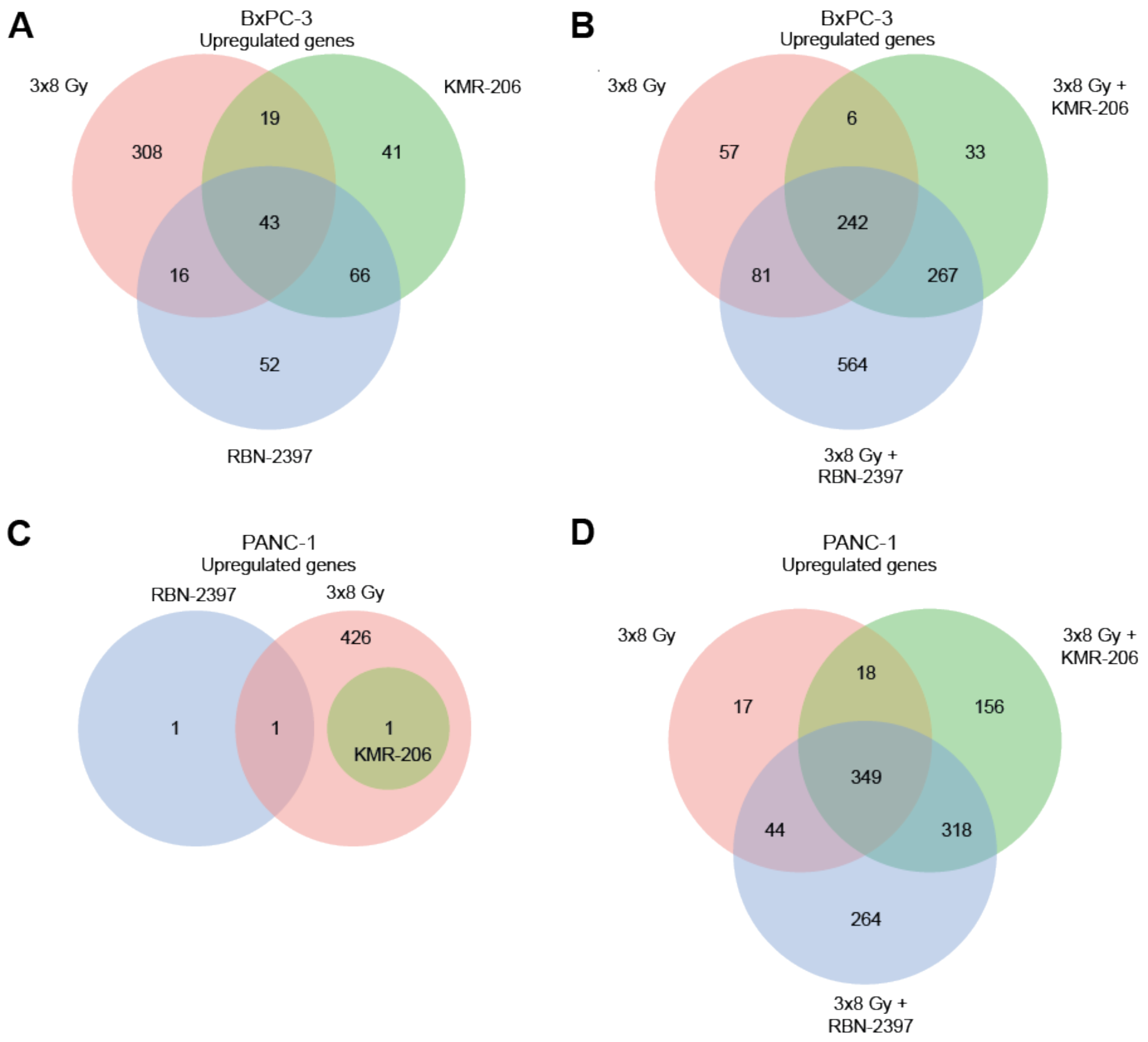

Fig. S3. Overlapping upregulated genes after radiation and PARP7i inhibitor treatments in BxPC-3 and PANC-1 cells. (A, C) Overlapping upregulated genes for 3x8 Gy, RBN-2397 and KMR-206 in (A) BxPC-3 and (C) PANC-1 cells. (B, D) Overlapping upregulated genes for 3x8 Gy, 3x8 Gy + RBN-2397 and 3x8 Gy + KMR-206 in (B) BxPC-3 and (D) PANC-1 cells.

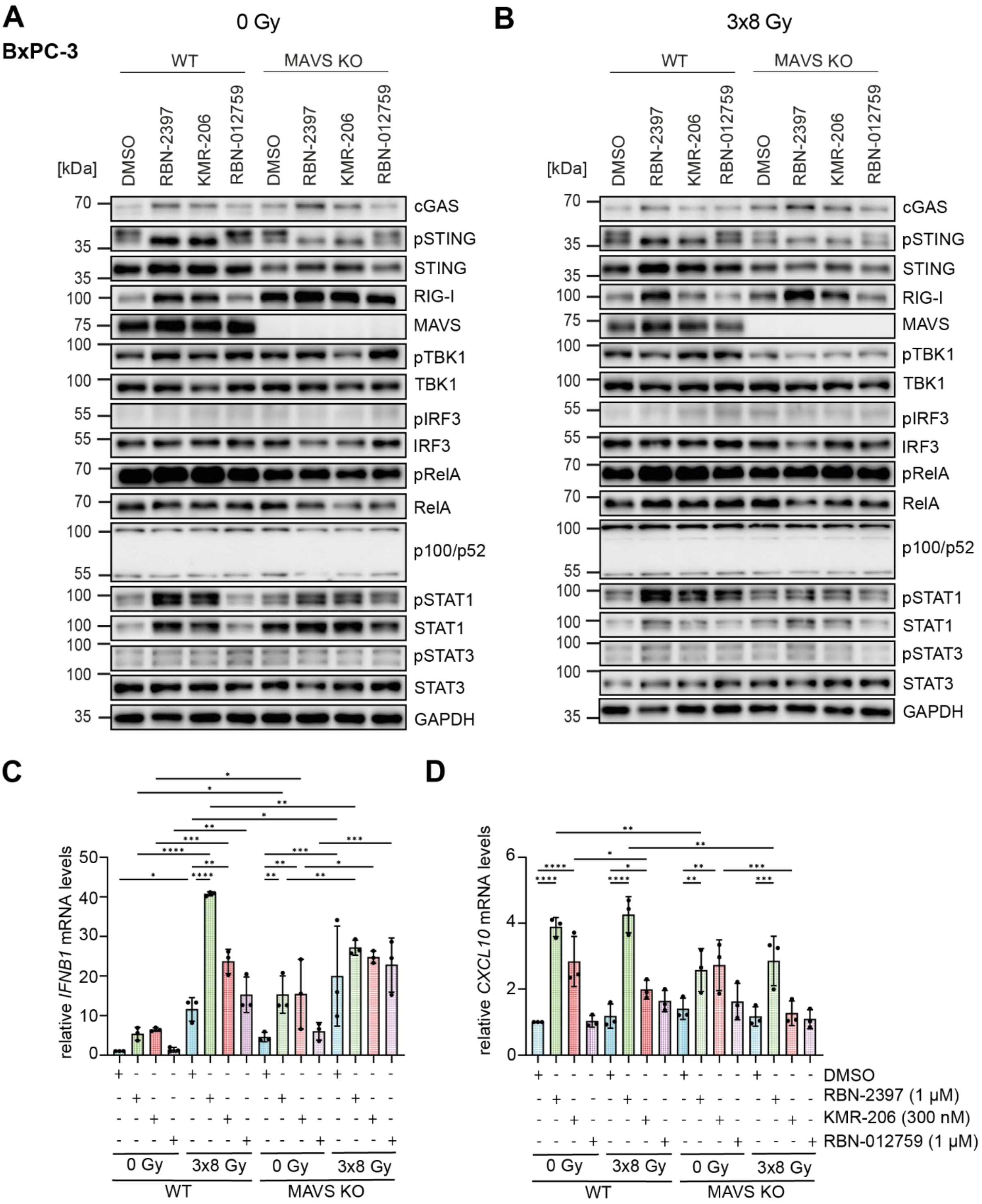

Fig. S4. The type I interferon response induced by radiation and PARP7 inhibition is partly MAVS-dependent in BxPC-3 cells. (A, B) Western blot analysis of WT and MAVS KO BxPC-3 cells treated with PARP7 and PARP14 inhibitors (A) without irradiation and (B) with 3x8 Gy X-ray. Inhibitors were added 24 h before irradiation and kept until harvesting 72 h after the last fraction. GAPDH was used as a loading control. (C, D) RT-qPCR analysis of the expression of *IFNB1* and *CXCL10* in BxPC-3 cells. Gene expression was normalized to TBP. Data points represent mean values ± standard deviation (N=3). One-way ANOVA with Tukey's post-hoc test was performed to determine significance (*≤ 0.05; **≤ 0.01; ***≤ 0.005, ****≤0.0001).

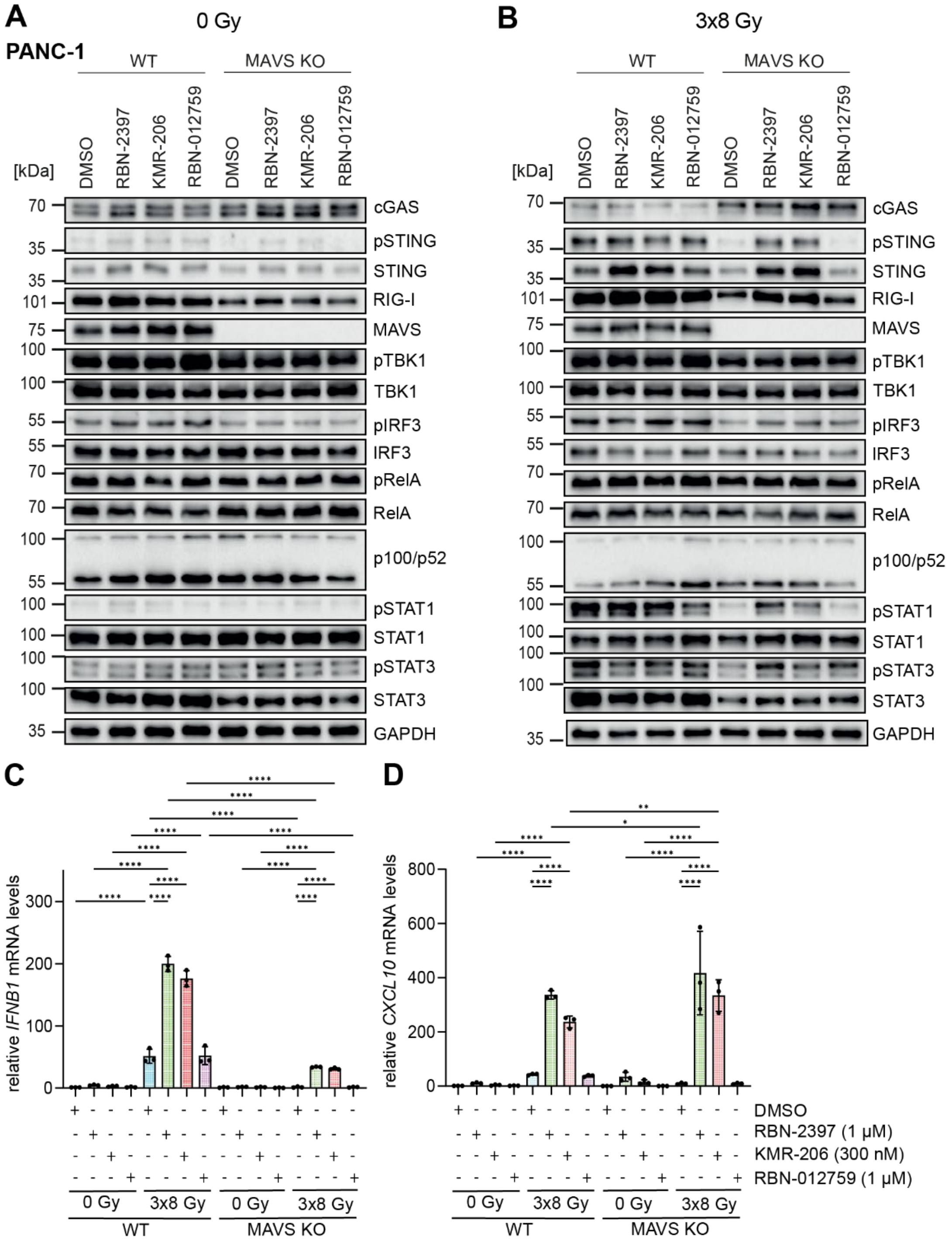

Fig. S5. The type I interferon response induced by radiation and PARP7 inhibition is partly MAVS-dependent in PANC-1 cells. (A, B) Western blot analysis of WT and MAVS KO PANC-1 cells treated with PARP7 and PARP14 inhibitors (A) without irradiation and (B) with 3x8 Gy X-ray. Inhibitors were added 24 h before irradiation and kept until harvesting 72 h after the last fraction. GAPDH was used as a loading control. (C-F) RT-qPCR analysis of the expression of *IFNB1* and *CXCL10* in PANC-1 cells. Gene expression was normalized to TBP. Data points represent mean values ± standard deviation (N=3). One-way ANOVA with Tukey's post-hoc test was performed to determine significance (*≤ 0.05; **≤ 0.01; ***≤ 0.005, ****≤0.0001).

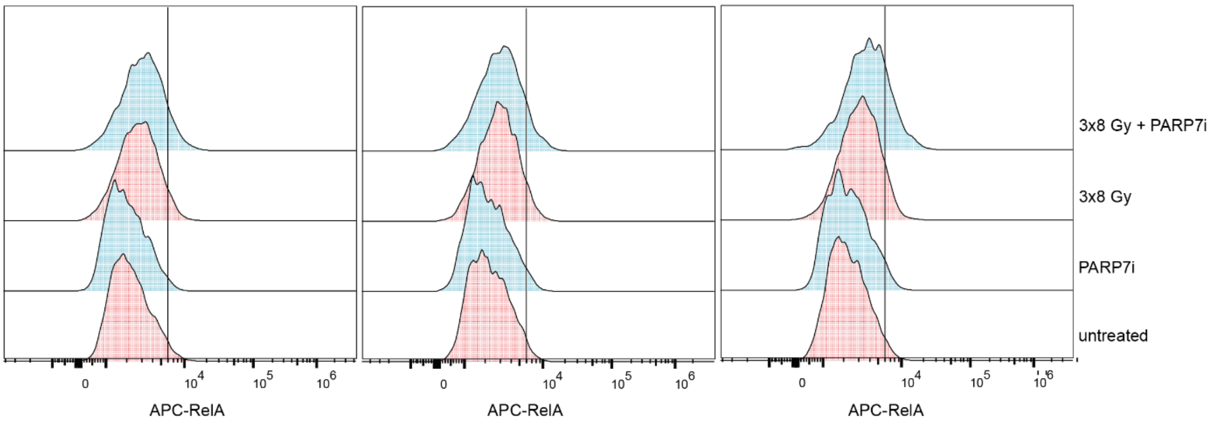

Fig. S6. Flow cytometry analysis of nuclear RelA in PANC-1. To quantify RelA nuclear translocation, nuclear proteins were immunostained with APC-RelA antibody and analyzed by flow cytometry in PANC-1 cells treated with the PARP7 inhibitor KMR-206 (300 nM), 3x8 Gy and their combination. The three sets of graphs represent three biological replicates. Cells were harvested 72 h after treatment. The solid lines represent the gate to determine the percentage of nuclear RelA-stained cells and each gate was adjusted based on the untreated sample. Flow cytometry measurements were analyzed with FlowJo_v10.8.1.

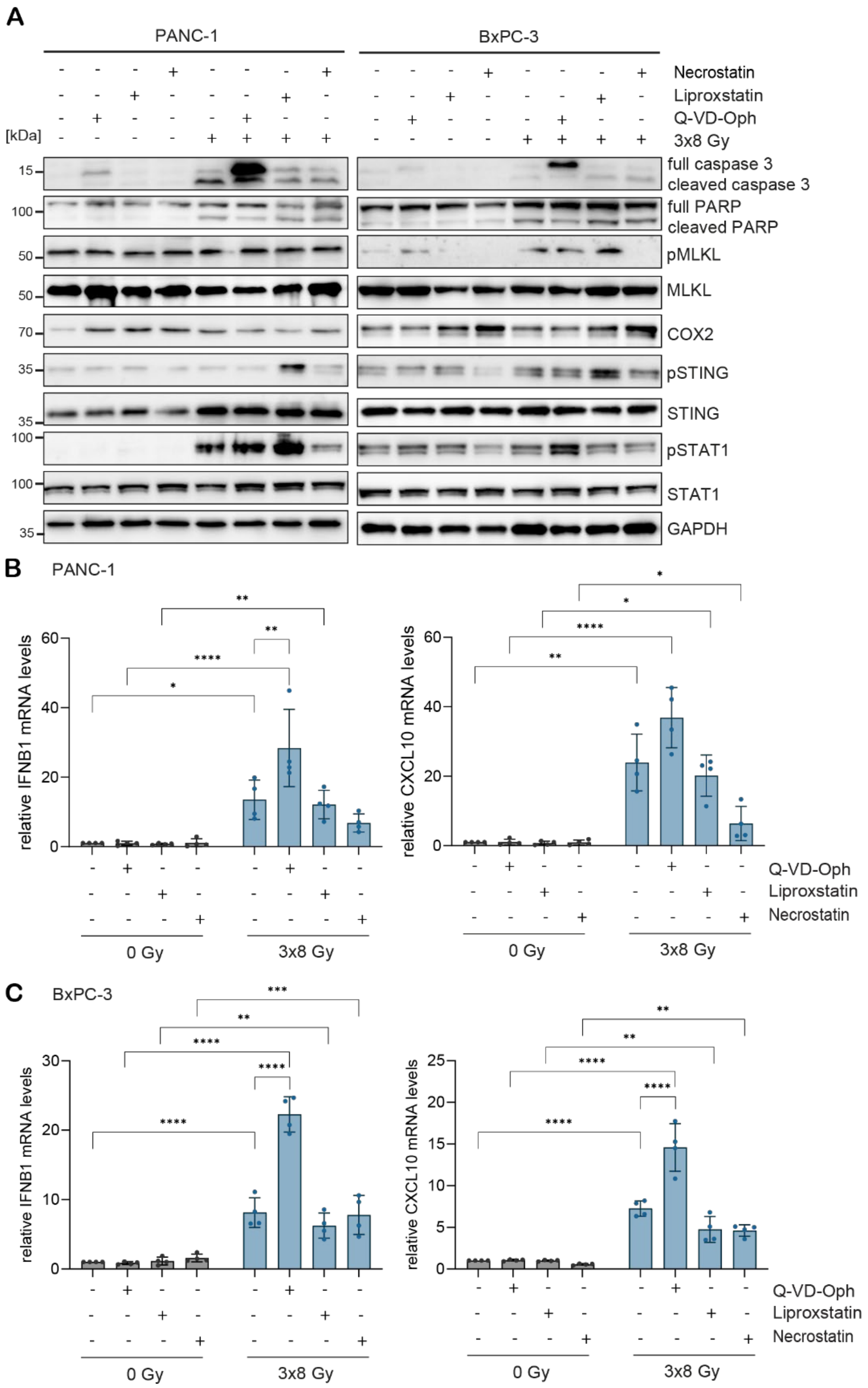

Fig. S7. Cell death pathways regulate immunogenic signaling in PDAC cells. (A) Western blot analysis of PANC-1 and BxPC-3 cells exposed to 3x8 Gy without or with ferroptosis inhibitor liproxstatin-1 (5 µM), necroptosis inhibitor necrostatin (10 µM) or apoptosis inhibitor Q-VD-Oph (10 µM). These agents were added to the cells before the first irradiation fraction and maintained until harvesting the cells 72 h after the last fraction. GAPDH was used as a loading control. (B, C) RT-qPCR analysis of the expression of *IFNB1* and *CXCL10* in (B) PANC-1 and (C) BxPC-3 cells. Gene expression was normalized to TBP. The experiments were performed in 3-4 biological replicates. Data points represent mean values ± standard deviation. One-way ANOVA with Tukey's post-hoc test was performed to determine significance (*≤ 0.05; **≤ 0.01; ***≤ 0.005, ****≤0.0001).

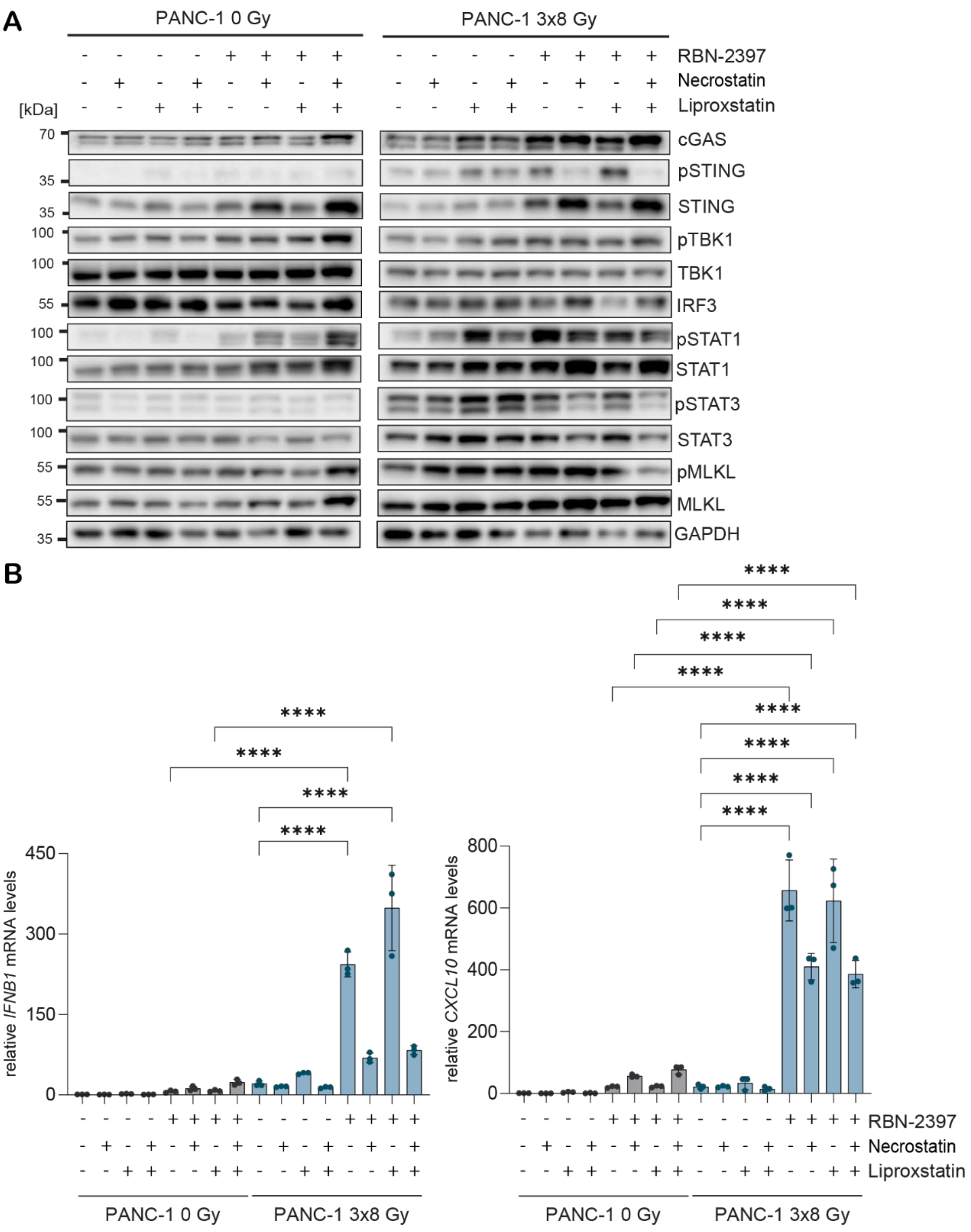

Fig. S8. Necroptosis promotes immunogenic signaling in PANC-1. (A) Western blot analysis of PANC-1 cells exposed to 3x8 Gy without or with the PARP7 inhibitor RBN-2397 (1 µM), necroptosis inhibitor necrostatin (10 µM), ferroptosis inhibitor liproxstatin-1 (5 µM), or a combination thereof. These agents were added to the cells before the first irradiation fraction and maintained until harvesting the cells 72 h after the last fraction. GAPDH was used as a loading control. (B) RT-qPCR analysis of the expression of *IFNB1* and *CXCL10*. Gene expression was normalized to TBP. The experiments were performed in three biological replicates. Data points represent mean values ± standard deviation. One-way ANOVA with Tukey's post-hoc test was performed to determine significance (*≤ 0.05; **≤ 0.01; ***≤ 0.005, ****≤0.0001).

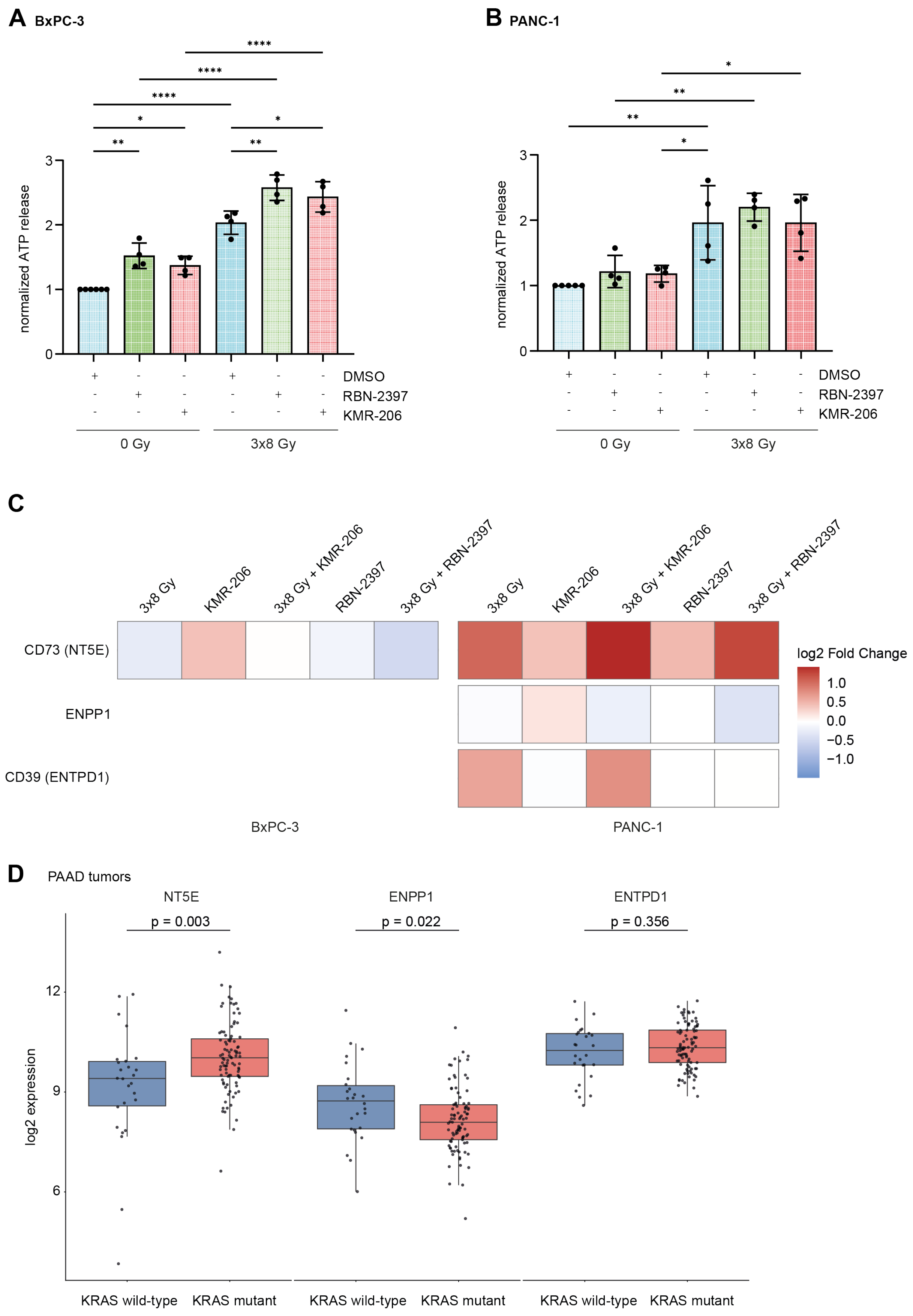

Fig. S9. Radiation induces immunogenic cell death in PDAC cells and the expression of ATP degrading enzymes in PANC-1. ATP release assay was used to measure immunogenic cell death in

(A) BxPC-3 and (B) PANC-1 cells irradiated with 3x8 Gy X-ray without or with the PARP7 inhibitors RBN-2397 (1 µM) and KMR-206 (300 nM). PARP7 inhibitors were added 24 h before irradiation and kept until the measurements 72 h after the last irradiation. The experiments were performed in four biological replicates. Data points represent mean values ± standard deviation. One-way ANOVA with Tukey's post-hoc test was performed to determine significance (*≤ 0.05; **≤ 0.01; ***≤ 0.005, ****≤0.0001). (C) Log2 fold changes in gene expression based on RNA-seq (Fig. 3) between indicated treated and control samples of genes involved in ATP degradation to adenosine. *ENPP1* and *CD39 (ENTPD1)* were not detected in BxPC-3. (D) *NT5E*, *ENPP1* and *ENTPD1* expression stratified by KRAS mutation status in TCGA PAAD tumors (KRAS wildtype n=26, KRAS mutant n=93).

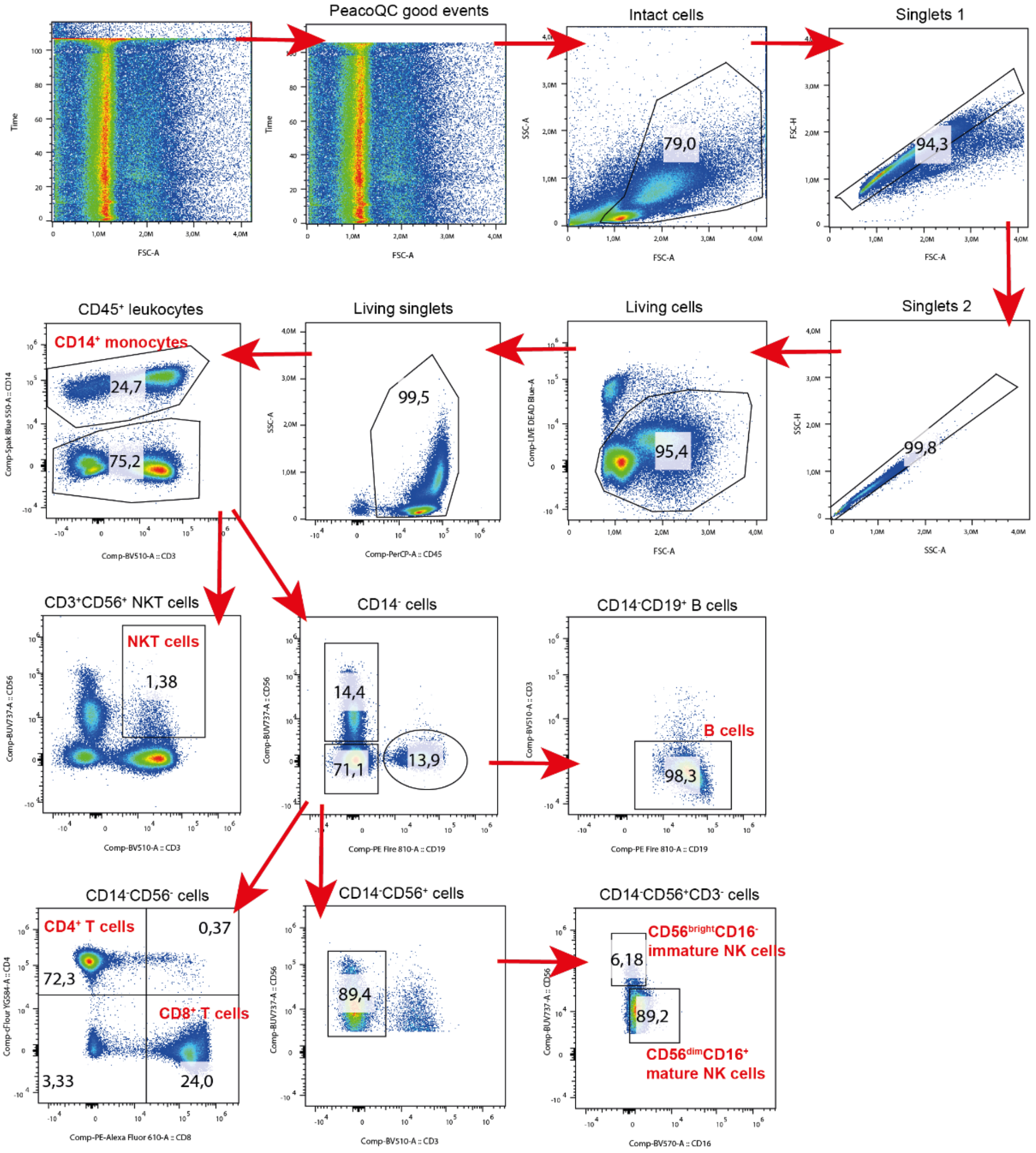

Fig. S10. Gating strategy for the identification of the immune cell subsets within peripheral blood mononuclear cells (PBMCs). Sequential gating strategy was applied to identify viable single CD45+ cells. Gating on specific lineage markers was employed to discriminate between different immune cell subsets (monocytes, NK, NKT, B cells, CD4+ and CD8+ T cells). For NK cells, two populations were identified. Only the more abundant CD56dimCD16+ mature NK cell population is discussed here and is referred to as 'NK cells'.

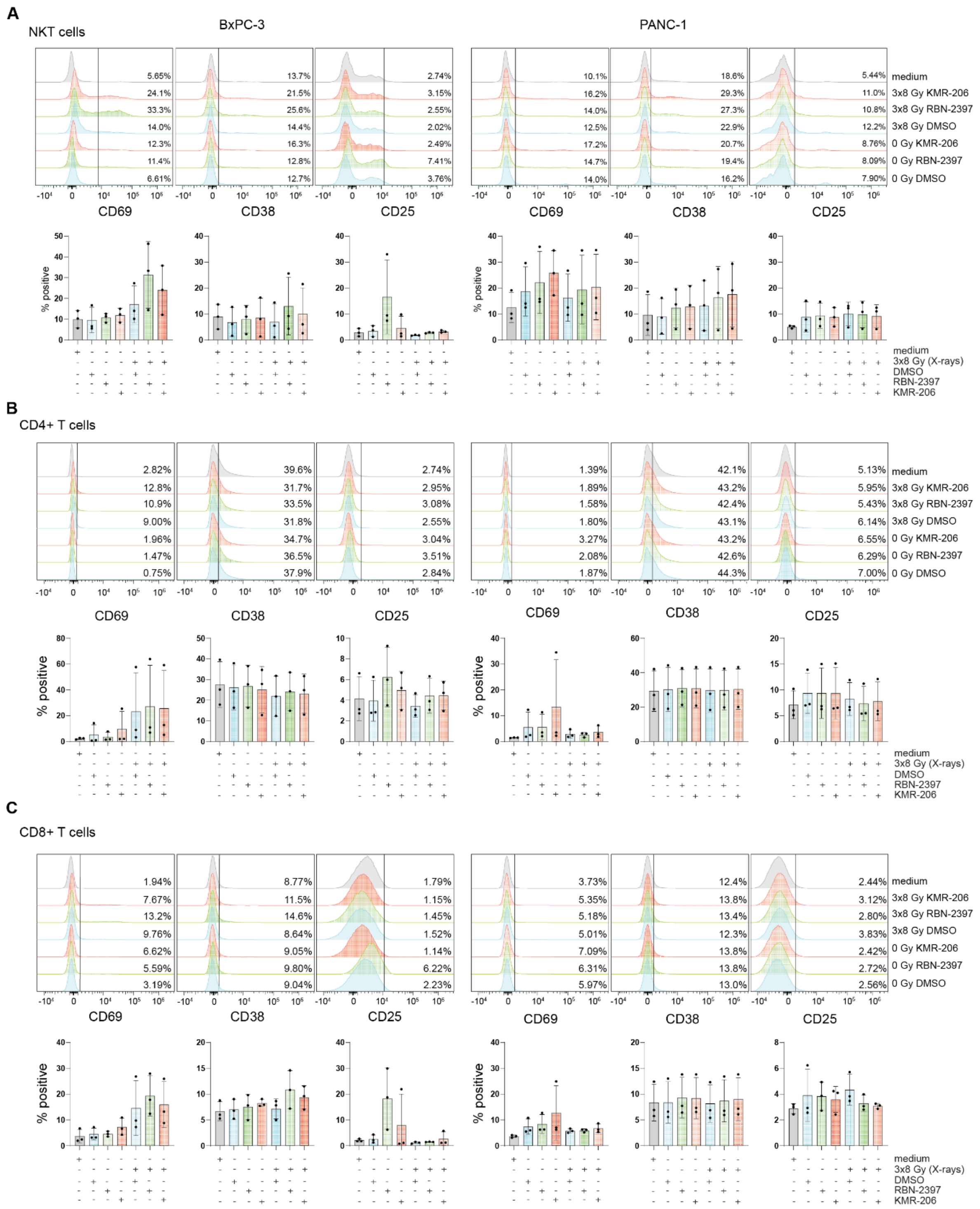

Fig. S11. Response of primary human NKT, CD4^{+} and CD8^{+} T cells to supernatants from PDAC cells treated with radiation and PARP7i. PBMCs from three different donors were incubated for 44 h in the presence of the culture medium or cell-free supernatants from BxPC-3 and PANC-1 cells treated with 3x8 Gy X-rays without or with the PARP7 inhibitors RBN-2397 (1 µM) or KMR-206 (300 nM). PARP7i were added 24 h before irradiation and kept until the measurements 72 h after the last irradiation. Human (A) NKT cells, (B) CD4^{+} T cells and (C) CD8^{+} T cells were identified according to lineage markers and their activation was assessed based on the analysis of activation markers CD25, CD38 and CD69 by spectral flow cytometry. Representative histograms of one donor (top) and percentages of marker-positive cells ± standard deviation of three PBMC donors (bottom) are shown. Statistical significance was assessed using one-way ANOVA with Tukey's post-hoc test (*≤ 0.05; **≤ 0.01; ***≤ 0.005, ****≤0.0001).

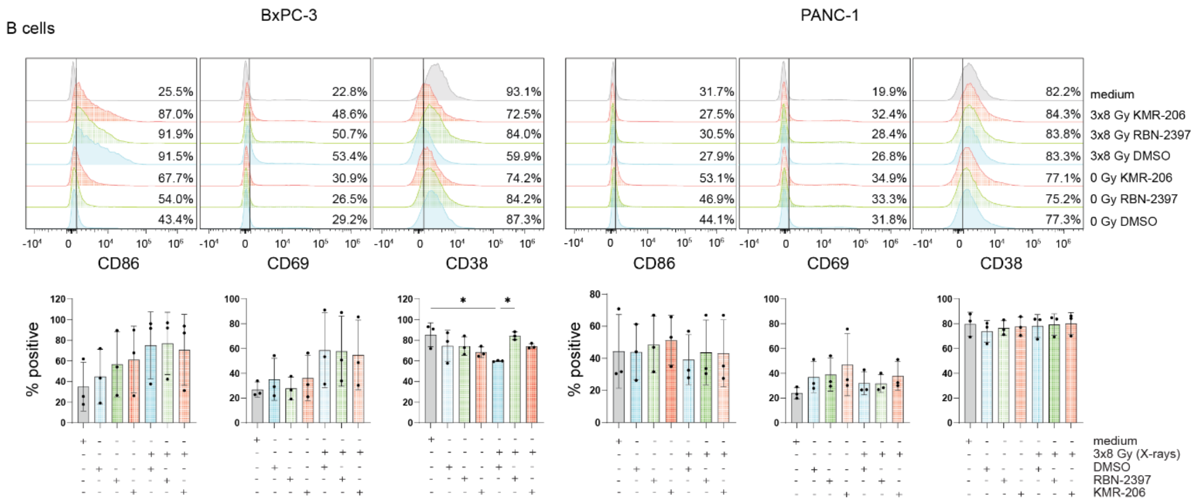

Fig. S12. Response of primary human B cells to supernatants from PDAC cells treated with radiation and PARP7i. PBMCs from three different donors were incubated for 44 h in the presence of the culture medium or cell-free supernatants from BxPC-3 and PANC-1 cells treated with 3x8 Gy X-rays without or with the PARP7 inhibitors RBN-2397 (1 µM) or KMR-206 (300 nM). PARP7i were added 24 h before irradiation and kept until the measurements 72 h after the last irradiation. Human B cells were identified according to lineage markers and their activation was assessed based on the analysis of activation markers CD86, CD69 and CD38 by spectral flow cytometry. Representative histograms of one donor (top) and percentages of marker-positive cells ± standard deviation of three PBMC donors (bottom) are shown. Statistical significance was assessed using one-way ANOVA with Tukey's post-hoc test (*≤ 0.05; **≤ 0.01; ***≤ 0.005, ****≤0.0001).

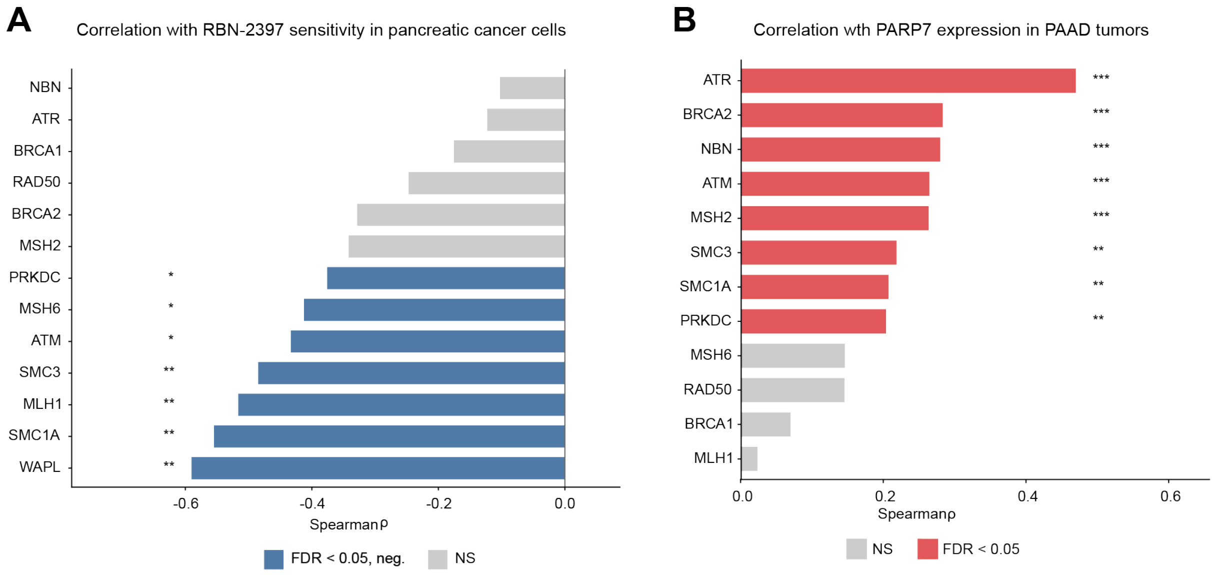

Fig. S13. PARP7 expression in pancreatic tumors correlates with genome integrity pathways. (A) Spearman correlations between expression of selected genes and RBN-2397 sensitivity in 37 pancreatic cancer cell lines. ** FDR<0.01, *** FDR<0.001. (B) Spearman correlations between PARP7 and selected genes expression in 178 TCGA PAAD tumor samples. ** FDR<0.01, *** FDR<0.001.

Table S1. RNA-seq analysis of BxPC-3 and PANC-1 cells before and after treatment with 3x8 Gy without or with the PARP7 inhibitors RBN-2397 (1 μM) and KMR-206 (300 nM). These agents were added to the cells 2 h before the first radiation fraction and maintained until harvesting the cells 72 h after the last fraction.

Table S2. RNA-seq analysis of repetitive elements in BxPC-3 and PANC-1 cells before and after treatment with 3x8 Gy without or with the PARP7 inhibitors RBN-2397 (1 μM) and KMR-206 (300 nM). These agents were added to the cells 2 h before the first radiation fraction and maintained until harvesting the cells 72 h after the last fraction.

Table S3. Immunophenotyping panel used in spectral flow cytometry analysis of PBMCs. mAbs used in both the full immunophenotyping and the lineage panel are depicted in bold. DC – dendritic cell, Treg – regulatory T cell.

Marker	Fluorochrome	Clone	Company	Cat. Number	Final dilution	Purpose
CD45RA	BUV395	5H9	BD Biosciences	750631	1:460	T cell and DC differentiation
**Viability**	**LIVE DEAD Blue**	**-**	**Thermo Fisher**	**L23105**	**1:500 (prestain)**	**Viability**
**CD56**	**BUV737**	**NCAM16.2**	**BD Biosciences**	**612766**	**1:70**	**NK cells**
CD195/CCR5	BUV805	3A9	BD Biosciences	748872	1:70	Chemokine receptor; monocyte, DC, T cell, B cell differentiation
CD197/CCR7	BV421	G043H7	BioLegend	353208	1:46	T cell differentiation
**CD3**	**BV510**	**SK7**	**BioLegend**	**344828**	**1:175**	**T cells, NKT cells**
**CD16**	**BV570**	**3G8**	**BioLegend**	**302036**	**1:140**	**Monocyte, NK cell, and DC differentiation**
CD279/PD-1	BV785	EH12.2H7	BioLegend	329930	1:140	T cell inhibitory receptor
**CD14**	**Spark Blue 550**	**63D3**	**BioLegend**	**367148**	**1:280**	**Monocyte differentiation**
**CD45**	**PerCP**	**2D1**	**BioLegend**	**368506**	**1:100**	**Leukocytes**
CD11b	PerCP-Cy5.5	LM2	BioLegend	393106	1:46	DC differentiation, monocyte activation
**TCR γδ**	**PerCP-eFluor710**	**B1.1**	**Thermo Fisher**	**46-9959-42**	**1:280**	**γδ T cells**
**CD4**	**cFluor YG584**	**SK3**	**CYTEK**	**R7-20041**	**1:116**	**CD4 T cells, NKT cells**
**CD8**	**PE-Alexa Flour 610**	**3B5**	**Thermo Fisher**	**MHCD0822**	**1:460**	CD8 T cells, NK cells and NKT cells

CD80	PE-Cy5	L307.4	BD Biosciences	559370	1:70	Monocyte, DC and B cell activation marker
CD25/IL2Rα	PE-Alexa Flour 700	CD25-3G10	Thermo Fisher	MHCD2524	1:140	Treg marker, activation marker on T cells, B cells, myeloid cells
CD183/CXCR3	PE-Vio770	CEW33D	Thermo Fisher	25-1839-42	1:200	Chemokine receptor; DC, T, and B cell differentiation
**CD19**	**PE-Fire 810**	**HIB19**	**BioLegend**	**302287**	**1:280**	**B cells**
CD86	APC	BU63	BioLegend	374208	1:280	Monocyte, DC and B cell activation marker
CD69	APC-R700	FN50	BD Biosciences	565154	1:93	Early activation marker expressed on leukocytes
HLA-DR	APC-Fire 750	L243	BioLegend	307657	1:175	T cell and monocyte activation, DC lineage marker
CD38	APC-Fire 810	HIT2	BioLegend	303549	1:200	Monocyte, DC, T and B cell activation/differentiation

Table S4. Antibodies

Rabbit anti-β-Actin	Cell Signaling	RRID: AB_2223172; 4970S; 1:1000 for WB
Rabbit anti-GAPDH	Cell Signaling	RRID: AB_10622025; 5174S; 1:1000 for WB
Rabbit anti-cGAS	Cell Signaling	RRID: AB_2732795; 15102S; 1:1000 for WB
Rabbit anti-cGAS	Proteintech	26416-1-AP; IF 1:200
Rabbit anti-pSTING	Cell Signaling	RRID: AB_2737062; 19781S; 1:1000 for WB
Rabbit anti-STING	Cell Signaling	RRID: AB_2732796; 13647S; 1:1000 for WB
Rabbit anti-RIG-I	Cell Signaling	RRID: AB_2269233; 3743S; 1:1000 for WB

Rabbit anti MAVS	Cell Signaling	RRID: AB_2798889; 24930S; 1:1000 for WB
Rabbit anti-pTBK1	Cell Signaling	RRID: AB_10693472; 5483S; 1:1000 for WB; 1:400 for IF
Rabbit anti-TBK1	Cell Signaling	RRID: AB_2827657; 38066S; 1:1000 for WB
Rabbit anti-pIRF3	Cell Signaling	RRID: AB_823547; 4947S; 1:1000 for WB
Rabbit anti-IRF3	Cell Signaling	RRID: AB_1904036; 4302S; 1:1000 for WB
Mouse anti-IkBa	Cell Signaling	RRID: AB_2084807; 4814; 1:1000 for WB
Rabbit anti-pRelA	Cell Signaling	RRID: AB_331284; 3033; 1:1000 for WB
Rabbit anti-RelA	Proteintech	RRID: AB_2178878; 10745-1-AP; 1:1000 for WB
Rabbit anti-p100/52	Cell Signaling	RRID: AB_10695537; 4882; 1:1000 for WB
Rabbit anti-pSTAT1	Cell Signaling	RRID: AB_561284; 9167S; 1:1000 for WB
Rabbit anti-STAT1	Cell Signaling	RRID: AB_2737027; 14994S; 1:1000 for WB
Rabbit anti-pSTAT3	Cell Signaling	RRID: AB_2491009; 9145; 1:1000 for WB
Mouse anti-STAT3	Abcam	RRID: AB_10901752; Ab119352; 1:1000 for WB
Rabbit anti-PARP1 / cleaved PARP1	Cell Signaling	RRID: AB_2160739; 9542; 1:1000 for WB
Rabbit cleaved caspase 3	Cell Signaling	RRID: RRID:AB_2070042; 9664; 1:1000 for WB
Rabbit anti-pMLKL	Cell Signaling	RRID: AB_2895044; 91689; 1:1000 for WB

Rabbit anti-MLKL	Cell Signaling	RRID: AB_2721822; 14993; 1:1000 for WB
Rabbit anti-COX2	Cell Signaling	RRID: AB_2571729; 12282; 1:1000 for WB
Rabbit anti-GPX4	Cell Signaling	RRID: AB_2924984; 52455; 1:1000 for WB
Goat anti-rabbit-IgG-HRP	Jackson ImmunoResearch	RRID: AB_2337937; 111-035-008; 1:10000 for WB
Goat anti-mouse-IgG-HRP	Jackson ImmunoResearch	RRID: AB_2313585; 115-035-008; 1:10000 for WB

Table S5. Raw data with uncropped western blot replicates.
