# Supplementary material for: PARP7 inhibitors enhance the immunogenic effects of radiation in pancreatic cancer cells
Source: Mol Ther Oncol. 2026 Jun 8;34(3):201258. doi: 10.1016/j.omton.2026.201258 (PMC13316578; doi:10.1016/j.omton.2026.201258)
Supplement: Document S2. Article plus supplemental information [file mmc5.pdf]

Molecular Therapy
Oncology
Original Article

PARP7 inhibitors enhance the immunogenic effects of radiation in pancreatic cancer cells

Niccolò Bragato,[1,2,3,10] Ana Beatriz Dias,[1,2,3,4,10] Anna Ohradanova-Repic,[6] Alma Dupanovic,[1,2,3] Filip Horvat,[1,2,5] Patrick Fischer,[3] Lisa-Marie Appel,[1,2,5] Lena Walch,[1,2,5] Ava Kleinwächter,[1,2,4,5] Anna Röhrer,[1,2,3] Sylvia Kerschbaum-Gruber,[1,2,3] Sandra Barna,[1] Piero Fossati,[3,7] Dietmar Georg,[1,2,3] Joachim Widder,[1,2] Klaus Podar,[7,8] Michael Cohen,[9] and Dea Slade[1,2,3,5]

[1]Department of Radiation Oncology, Medical University of Vienna, Währinger Gürtel 18-20, 1090 Vienna, Austria; [2]Comprehensive Cancer Center, Medical University of Vienna, Spitalgasse 23, 1090 Vienna, Austria; [3]MedAustron Ion Therapy Center, Marie-Curie Strasse 5a, 2700 Wiener Neustadt, Austria; [4]Vienna Biocenter PhD Program, a Doctoral School of the University of Vienna and the Medical University of Vienna, 1030 Vienna, Austria; [5]Max Perutz Labs, Vienna Biocenter Campus (VBC), Dr.-Bohr-Gasse 9, 1030 Vienna, Austria; [6]Medical University of Vienna, Center for Pathophysiology, Infectiology and Immunology, Institute for Hygiene and Applied Immunology, Lazarettgasse 19, 1090 Vienna, Austria; [7]Division of Molecular Oncology and Hematology, Department of Basic and Translational Oncology, Karl Landsteiner University of Health Sciences, 3500 Krems an der Donau, Austria; [8]Division of Internal Medicine 2, University Hospital Krems, 3500 Krems and der Donau, Austria; [9]Department of Chemical Physiology and Biochemistry, Oregon Health and Science University, 3181 SW Sam Jackson Pk. Road, Portland, OR 97239, USA

Pancreatic ductal adenocarcinoma (PDAC) is the most frequent type of pancreatic cancer with a poor prognosis and resistance to conventional radio- and chemotherapy. Radiation can induce pro-inflammatory signaling by activating the type 1 interferon (IFN-I) response, which can be enhanced by targeting negative regulators of the IFN-I pathway, such as ADP-ribosyltransferase PARP7. Here, we show that PARP7 inhibitors (PARP7i) enhance radiation-induced cell death and promote STING- and NF-κB-dependent immunogenic signaling in PDAC cells, leading to inflammatory gene expression and cytokine release. These effects were most pronounced in BxPC-3 cells, which exhibit higher baseline expression of *PARP7*, *AHR*, and interferon response genes. PARP7i potentiated the immunogenic effects of hypofractionated radiation by inducing a STING-dependent IFN-I response, leading to immunogenic cell death and activation of monocytes and NK cells. Carbon ion irradiation elicited stronger immunogenicity than X-rays when combined with PARP7i. KRAS-mutated PANC-1 cells showed a higher expression of enzymes that convert ATP to immunosuppressive adenosine, which was enhanced by radiation. This may explain why PARP7i were more effective as monotherapy in PANC-1 cells, promoting NK cell activation. These findings support further evaluation of PARP7i in PDAC in combination with radiotherapy or as monotherapy, depending on the immunosuppressive effects of radiation.

INTRODUCTION

Pancreatic ductal adenocarcinoma (PDAC) has a poor prognosis (5-year survival 12.8%) due to late diagnosis, metastasis, and resistance to immune checkpoint inhibitors, largely driven by an immunosuppressive tumor microenvironment (TME). Mutations of the KRAS oncogene contribute to the immunosuppressive TME in PDAC by promoting accumulation of myeloid-derived suppressor cells (MDSCs) and regulatory T lymphocytes (Tregs), and exclusion of cytotoxic CD8+ T cells.[1–3] PDAC patients with high cytotoxic T cell infiltration show longer overall survival,[4] suggesting that therapies that stimulate anti-tumor immune response hold promise for treating PDAC.[5]

Radiation therapy not only induces cytotoxic DNA damage but also stimulates anti-tumor immunity through release of damage-associated molecular patterns (DAMPs), activation of antigen-presenting cells, and induction of type 1 interferon (IFN-I) response and NF-κB signaling.[6] Cytosolic dsDNA and dsRNA activate the cGAS-STING and RIG-I-MAVS pathways, respectively, leading to TBK1-mediated activation of IRF3 and NF-κB, *IFNB1* transcription, and downstream JAK-STAT signaling.[7–12] This induces interferon-stimulated genes (ISGs), chemokines, and cytokines that promote recruitment and activation of cytotoxic T cells and other immune cells.

The central step for activation of the canonical NF-κB pathway is phosphorylation of the IκB kinase (IKK) complex.[13] The activated

Received 30 March 2026; accepted 3 June 2026;
https://doi.org/10.1016/j.omton.2026.201258.

[10]These authors contributed equally

Correspondence: Dea Slade, Department of Radiation Oncology, Medical University of Vienna, Währinger Gürtel 18-20, 1090 Vienna, Austria.
E-mail: dea.slade@meduniwien.ac.at

Published by Elsevier Inc. on behalf of The American Society of Gene and Cell Therapy.
This is an open access article under the CC BY license (http://creativecommons.org/licenses/by/4.0/).

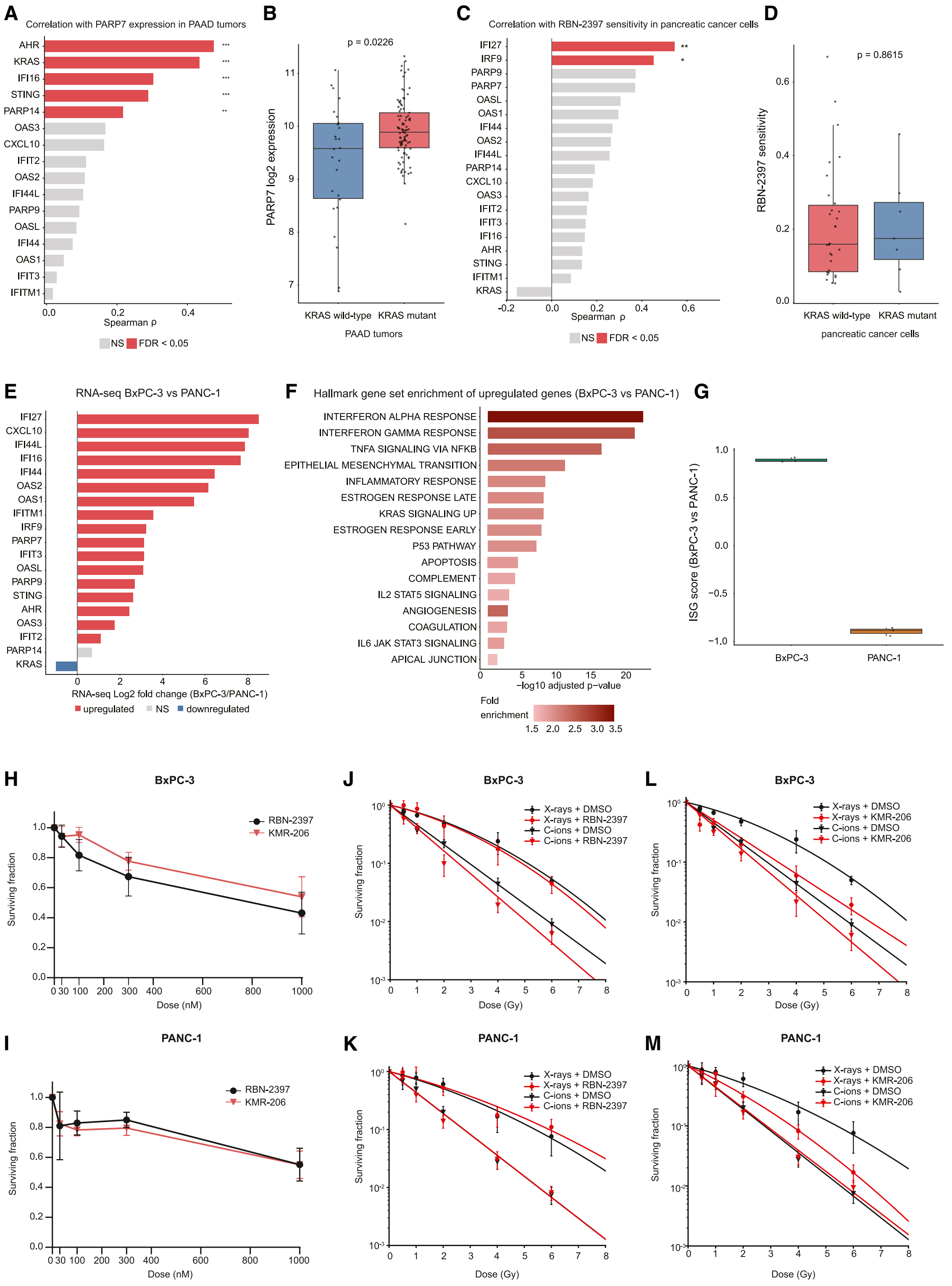

(legend on next page)

IKK complex phosphorylates the inhibitor of NF-κB alpha (IκBα), thereby triggering its proteasomal degradation.[13] This enables the release of the NF-κB heterodimer, primarily RelA/p50, which translocates to the nucleus and induces the expression of inflammatory genes such as *IFNB1*, *IL6*, *TNF* or *CXCL10*.[13]

We previously showed that hypofractionated X-ray irradiation (3 × 8 Gy) induces immunogenic cell death and IFN-I signaling in PDAC cells via cGAS-STING, RIG-I-MAVS, and NF-κB activation.[14] While particle radiation with protons did not show superior effects compared to photons (X-ray),[14] particle radiotherapy with carbon ions (C-ions) may induce a stronger anti-tumor immunogenic response due to higher ionization density (quantified by the linear energy transfer, LET) that results in clustered DNA damage and a greater release of DAMPs.[15]

PARP inhibitors can enhance radiation-induced immune responses. PARP1/2 inhibitors activate cGAS-STING signaling,[16,17] while PARP7 inhibitors (PARP7i) enhance anti-tumor immunity by relieving suppression of interferon and NF-κB pathways.[18,19] The known substrates of PARP7 include TBK1,[20] the RelA (p65) subunit of NF-κB,[21] FRA1 that suppresses RIG-I-MAVS signaling by inhibiting IRF1,[22,23] and the transcriptional co-activator CBP/p300 that induces *IFNB1* expression.[24] PARP7 expression is regulated by aryl hydrocarbon receptor (AHR), which acts as a ligand-activated transcription factor.[25]

As the first PARP7i to have entered clinical trials, RBN-2397 was shown to induce durable tumor regression in CT26 colon carcinoma-bearing mice, which is mediated by CD8+ T cells and dependent on the presence of PARP7, STING, and TBK1.[18] KMR-206 was later developed as a PARP7i with a similar potency but weaker immunogenic and anti-proliferation effects compared to RBN-2397.[19] PARP7 knockout (KO) in breast cancer or PDAC cells reduces tumor growth in mice by increasing the infiltration of immune cells.[21,26] PARP14 inhibition also promotes pro-inflammatory macrophage polarization and improves immunotherapy responses in melanomas.[27,28] Based on this, we investigated PARP1/2, PARP7, and PARP14 inhibitors combined with photon or C-ion radiation in PDAC cells BxPC-3 (KRAS wild-type, WT) and PANC-1 (KRAS G12D).

We found that PARP7i exert stronger cytotoxic effects in BxPC-3 cells, which have a higher expression of *PARP7*, *AHR*, and interferon response genes. PARP7i significantly enhanced radiation-induced cell death and immunogenic signaling, increasing IFNB1 and pro-inflammatory cytokines (CXCL10, IL-6). This effect depended on STING in both PDAC cell lines and NF-κB in KRAS-mutated PANC-1 cells. The induction of immunogenic cell death and the release of cytokines led to activation of immune cells such as monocytes and NK cells in BxPC-3 cells treated with hypofractionated radiation and PARP7i. Conversely, in PANC-1 cells PARP7i alone, but not in combination with radiation, activated NK cells. Overall, our findings demonstrate that PARP7i can enhance immunogenic and anti-tumor response in PDAC, revealing mechanistic links between PARP7, STING, and NF-κB signaling and highlighting differential responses based on PARP7 expression.

RESULTS

PARP7i reduce the viability of irradiated pancreatic cancer cells

High *PARP7* levels correlate with a worse survival in PDAC patients[29] and PARP7 KO in PDAC cells reduces tumor growth in mice by increasing the infiltration of immune cells.[26] PARP7i sensitivity seems to depend on the expression levels of *PARP7*, *AHR*, and interferon response genes, given that (1) induction of *PARP7* expression by AHR agonists sensitizes them to PARP7i,[30] (2) cells expressing higher levels of *PARP7* and interferon response genes are more sensitive to PARP7i,[18] while (3) KOs of AHR, PARP7, and IFN-I signaling genes (STING, IRF3, IFNB1, STAT2, IRF9) confer resistance to PARP7i.[18,31]

The analysis of 178 PAAD (pancreatic adenocarcinoma) patient samples from firebrowse.org revealed a positive correlation between *PARP7* expression and the expression of *AHR*, interferon response genes and *KRAS* as well as oncogenic KRAS mutations (Figures 1A and 1B). Moreover, pancreatic cancer cell lines that express higher levels of *PARP7*, *AHR*, and interferon response genes are more sensitive to the PARP7i RBN-2397[32] (Figure 1C). However, KRAS mutations do not appear to affect sensitivity to PARP7i in pancreatic cancer cell lines (Figure 1D).

For this study we chose two PDAC cell lines that have variable *PARP7* expression levels. BxPC-3 (KRAS WT) shows a significantly higher expression of *PARP7*, *AHR*, and interferon response genes,

Figure 1. PARP7 inhibitors are more toxic in cells with high levels of PARP7, AHR, and interferon response genes, and increase the sensitivity of PDAC cells to radiation

(A) Spearman's correlations between *PARP7* and *AHR*, *KRAS*, and selected ISGs in 178 TCGA PAAD tumor samples. ** FDR<0.01, *** FDR<0.001. (B) PARP7 expression stratified by KRAS mutation status in TCGA PAAD tumors (KRAS wild-type $N = 26$, KRAS mutant $N = 93$). Two-sided Wilcoxon rank-sum test was performed to determine significance. (C) Spearman's correlations between RBN-2397 sensitivity and expression of *PARP7*, *AHR*, selected ISGs and *KRAS* in 37 pancreatic cancer cell lines. ** FDR<0.01, *** FDR<0.001. (D) RBN-2397 sensitivity stratified by KRAS mutation status in 37 pancreatic cancer cell lines (wild-type $N = 7$, mutant $N = 30$). Two-sided Wilcoxon rank-sum test was performed to determine significance. (E) RNA-seq log2 fold changes of *PARP7*, *AHR*, selected ISGs and *KRAS* between BxPC-3 and PANC-1 cells (three biological replicates). (F) ORA of Hallmark gene sets (MSigDB) for genes significantly upregulated in BxPC-3 vs. PANC-1. Pathways with BH-adjusted $p < 0.05$ are shown. (G) ISG score in BxPC-3 and PANC-1 cells (three biological replicates per cell line). Central line in boxplots shows median; each point represents one sample. (H–M) Cell survival curves of (H, J, L) BxPC-3 and (I, K, M) PANC-1 cells. (H, I) Cells were treated with 30, 100, 300, or 1,000 nM of PARP7 inhibitors RBN-2397 or KMR-206 for 48 h. Cell survival was determined by clonogenic assays. The experiments were performed in three biological replicates. (J–M) Cell survival curves after X-ray (circle) or C-ion irradiation (triangle) without (black) or with PARP7i (red). KMR-206 (300 nM) and RBN-2397 (1 μM) were added 24 h prior to irradiation and removed 24 h after irradiation. The experiments were performed in four biological replicates. The linear-quadratic model was applied for survival curve fitting.

enrichment of the interferon response pathway, and a higher ISG score (Figures 1E–1G). We performed colony formation assays to test whether PARP7i impair proliferation of these two PDAC cell lines when applied alone or in combination with radiation. Both PARP7i induced cytotoxic effects in BxPC-3 and PANC-1 cells in a dose-dependent manner (Figures 1H and 1I). RBN-2397 exhibited stronger cytotoxic effects than KMR-206 in BxPC-3 cells, which were more sensitive to PARP7i than PANC-1 cells (Figures 1H and 1I). These results support PARP7 expression levels as a biomarker for PARP7i sensitivity, while suggesting that KRAS mutation status likely does not play a significant role.

Next, PDAC cells were exposed to different doses of X-rays or C-ions without or with PARP7i (Figures 1J–1M). As expected, due to their higher LET, C-ions were more potent in killing PDAC cells compared to X-rays. KMR-206 sensitized both PDAC cell lines to X-rays (Figures 1L and 1M). Both RBN-2397 and KMR-206 sensitized only BxPC-3 cells to C-ions (Figures 1J and 1L). These findings indicate that PARP7i exhibit higher cytotoxicity in BxPC-3 cells, both as monotherapy or in combination with radiation, and that KMR-206 shows a stronger radiosensitizing effect than RBN-2397.

PARP7i enhance radiation-induced type 1 interferon response in pancreatic cancer cells

In addition to directly killing cancer cells, PARP7i and radiation can also activate anti-tumor innate immune response.[29,33] To test whether they can stimulate immunogenic signaling in PDAC, we treated BxPC-3 and PANC-1 with 8 Gy X-rays or C-ions and 3 × 8 Gy X-rays (8 Gy on three consecutive days). In addition, we tested the combinatorial effects of radiation and PARP7i RBN-2397 and KMR-206, as well as the DNA damage response inhibitor olaparib targeting PARP1/2 and the PARP14 inhibitor RBN-012759. Immunogenic signaling was analyzed by western blotting to determine the expression levels and the phosphorylation status of the cGAS-STING signaling pathway (cGAS, STING, TBK1, IRF3, RelA, and STAT1), and by quantitative reverse-transcription PCR (RT-qPCR) to determine the transcriptional induction of *IFNB1* and the ISG *CXCL10*. PARP7i and hypofractionated radiation (3 × 8 Gy X-rays) showed synergistic effects in BxPC-3 and PANC-1 cells as judged by an increase in pSTAT1, *IFNB1*, and *CXCL10* (Figures S1 and S2). In both cell lines, PARP1/2 and PARP14 inhibitors failed to activate the IFN-I response (Figures S1 and S2). PARP7i showed opposing effects on pSTING, reducing its levels in BxPC-3 cells while increasing them in PANC-1 cells (Figures S1 and S2).

Given that C-ions exhibited higher lethality (Figure 1), whereas hypofractionated X-ray irradiation showed the strongest induction of immunogenic signaling (Figures S1 and S2), we tested whether hypofractionated C-ion irradiation elicits immunogenic effects comparable to hypofractionated 3 × 8 Gy X-ray irradiation. We exposed BxPC-3 and PANC-1 cells to three consecutive isoeffective doses of C-ions calculated based on relative biological effectiveness (RBE_{10}) (3 × 3.3 Gy for BxPC-3 and 3 × 3.5 Gy for PANC-1) (Figure 2). In BxPC-3, isoeffective hypofractionated C-ion irradiation induced *IFNB1*, ISGs *CXCL10*, and *IFIT1*, as well as the proinflammatory cytokine *IL6* more than 3 × 8 Gy X-rays (Figure 2A). In PANC-1, *IFNB1*, *CXCL10*, *IFIT1*, *IL6*, and *TNF* were induced to a similar extent after 3 × 3.5 Gy C-ions and 3 × 8 Gy X-rays (Figure 2B). The induction of all genes except for *CXCL10* was lower in PANC-1 compared to BxPC-3 (Figure 2).

Overall, our findings demonstrate that combining PARP7i with radiotherapy elicits synergistic immunogenic effects. Moreover, they suggest that hypofractionated C-ion therapy in PDAC may represent a promising alternative to X-rays, with the potential to enhance anti-tumor immune responses while limiting damage to surrounding healthy tissue, although further evidence is needed to confirm whether its immunogenic effects are indeed superior. In subsequent experiments, we used 3 × 8 Gy X-rays due to limitations in experimental beam time for C-ions.

Genome-wide transcriptional effects of radiation and PARP7i in pancreatic cancer cells

To examine genome-wide transcriptional changes induced by PARP7i and radiation, we performed RNA sequencing (RNA-seq) analysis of BxPC-3 and PANC-1 cells treated with 3 × 8 Gy X-rays without or with the PARP7i RBN-2397 and KMR-206 (Figures 3 and S3; Tables S1 and S2). Radiation alone upregulated 386 and 428 genes, and downregulated 150 and 198 genes in BxPC-3 and PANC-1 cells, respectively (Figure 3A). PARP7i alone upregulated 169 (KMR-206) and 177 genes (RBN-2397) in BxPC-3 cells but had no effect in PANC-1 cells (Figure 3A). Of ∼170 genes that were upregulated after PARP7i alone in BxPC-3, 43 were also upregulated after radiation and enriched for inflammatory response (e.g., *IFIT2*, *CXCL5*, *CCL20*, *CXCL1*) and 66 genes were shared between the two PARP7i (e.g., *CXCL10*, *IFIT1*, *IFIT3*, *OAS1*, *OAS3*, *MX1*) (Figure S3A).

In both cell lines, PARP7i augmented the transcriptional effects of radiation, whereby RBN-2397 showed a stronger effect compared to KMR-206 (Figures 3A–3C). The most highly upregulated pathways were inflammatory response, type 1 interferon response and NF-κB signaling (Figure 3D), in accordance with the previously conducted RT-qPCR analyses. The most strongly upregulated genes were ISGs such as *MX1*, *IFIT1*, *IFIT2*, *IFIT3*, *IFI6*, *CXCL10*, *CXCL11*, *CCL5*, *ISG15*, and cytosolic RNA sensors (e.g., *OAS1*, *OAS2*, *OASL*). Given the strong induction of repetitive elements (ERV/LTR, long terminal repeat), especially after combinatorial treatments (Figures 3B and 3C; Table S2), upregulation of cytosolic RNA sensors may contribute to IFN-I signaling. Interestingly, the induction of repetitive elements was more pronounced with RBN-2397 than KMR-206 (Figure 3B). Taken together, in BxPC-3, PARP7i alone induced immunostimulatory genes such as *CXCL10*, whereas in PANC-1 radiation combined with RBN-2397 was the most potent inducer of cytokine and chemokine expression.

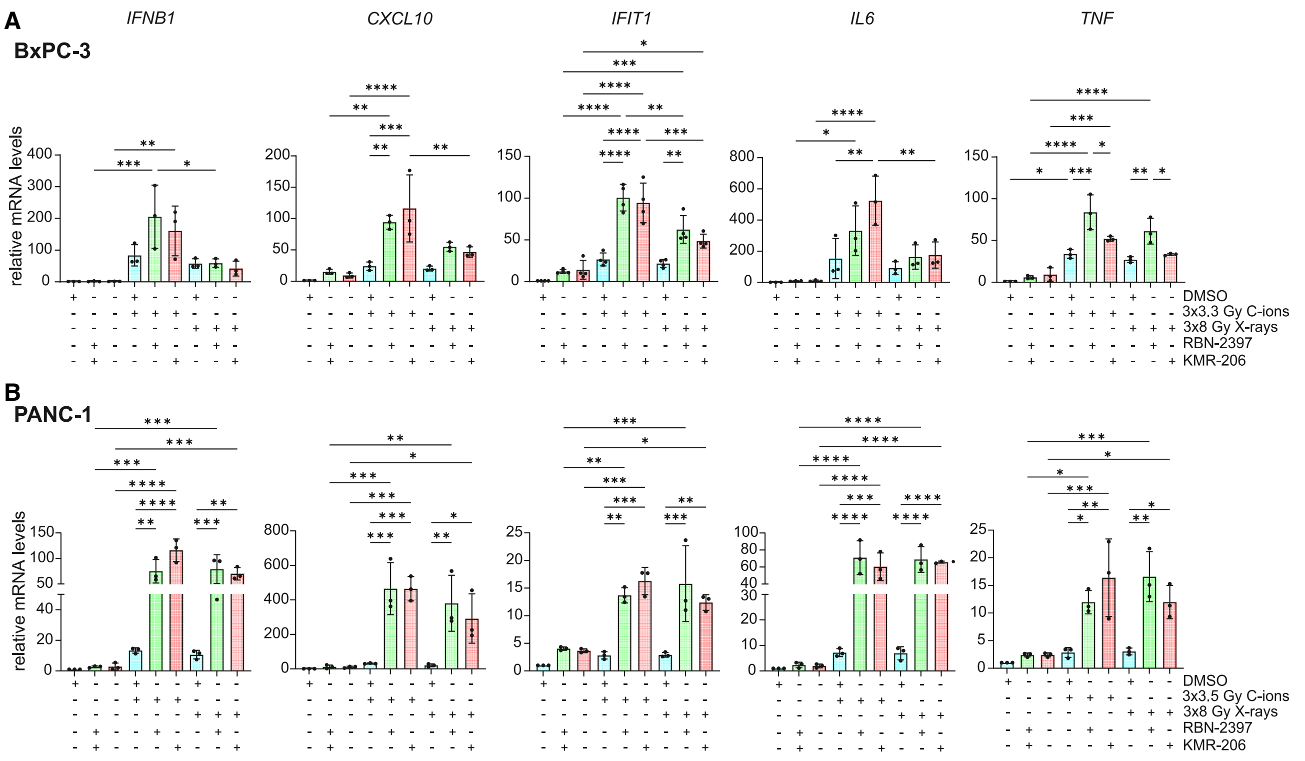

Figure 2. A combination of PARP7 inhibitors with the hypofractionated isoeffective dose of C-ions induces comparable immunogenic effects to 3 × 8 Gy X-ray irradiation

RT-qPCR analysis of the expression of *IFNB1*, *IFIT1*, *CXCL10*, *IL6*, *and TNF* in (A) BxPC-3 and (B) PANC-1 cells. Isoeffective doses for C-ions waere determined based on RBE_{10} calculated using survival curves in Figure 1. The isoeffective dose for 8 Gy X-rays is 3.3 Gy C-ions for BxPC-3 and 3.5 Gy for PANC-1 cells. The PARP7 inhibitors RBN-2397 (1 μM) and KMR-206 (300 nM) were added 24 h before irradiation and kept until harvesting 72 h after the last fraction. Gene expression was normalized to TBP. The experiments were performed in three biological replicates. Data points represent mean values ±standard deviation. One-way ANOVA with Tukey's post hoc test was performed to determine significance (*≤ 0.05; **≤ 0.01; ***≤ 0.005, ****≤0.0001).

Immunogenic signaling induced by PARP7i is STING-dependent in irradiated BxPC-3 cells

To understand the mechanistic basis of immunogenic effects induced by radiation and PARP7i in PDAC cells, we assessed whether the immunogenic response is STING-, MAVS-, or NF-κB-dependent. To this end, we used CRISPR/Cas9 KOs of STING and MAVS[14] and we treated the cells with the NF-κB inhibitor (NF-κBi) BI605906. BI605906 inhibits IKKβ and thereby prevents activation of RelA (p65).[34] We investigated the effect of the STING/MAVS KO or NF-κBi on the induction of the IFN-I response after 3 × 8 Gy in combination with PARP7i (Figure 4). STING KO in BxPC-3 cells abolished the induction of pIRF3, pSTAT1, *IFNB1*, and *CXCL10* after radiation alone or in combination with PARP7i compared to WT cells (Figure 4). In BxPC-3 MAVS KO cells, the effects of radiation and PARP7i were reduced but not abrogated (Figure S4), suggesting that STING plays a more important function in BxPC-3 than MAVS, as previously observed.[14]

Surprisingly, inhibition of NF-κB, particularly in combination with PARP7i, resulted in a pronounced upregulation of *IFNB1* after 3 × 8 Gy (Figure 4C). While NF-κB inhibition reduced radiation-induced *CXCL10* expression, it had no effect in combination with RBN-2397 and instead enhanced the effects of KMR-206 (Figure 4D).

NF-κBi severely suppressed non-canonical NF-κB signaling, as indicated by reduced levels of p100/p52 (Figure 4B). The non-canonical NF-κB pathway was shown to block *IFNB1* expression by epigenetic silencing of the *IFNB1* promoter.[35] This provides a plausible explanation for the observed increase in *IFNB1* expression upon inhibition of non-canonical NF-κB signaling in BxPC-3 cells. However, it remains unclear why the effect of NF-κBi is more pronounced in PARP7i-treated cells than in cells exposed to irradiation alone.

Taken together, these findings suggest that STING is the primary pathway driving the IFN-I response following PARP7 inhibition in BxPC-3 cells. Furthermore, NF-κB inhibition appears to enhance the immunostimulatory effects of PARP7i, most likely through suppression of non-canonical NF-κB signaling.

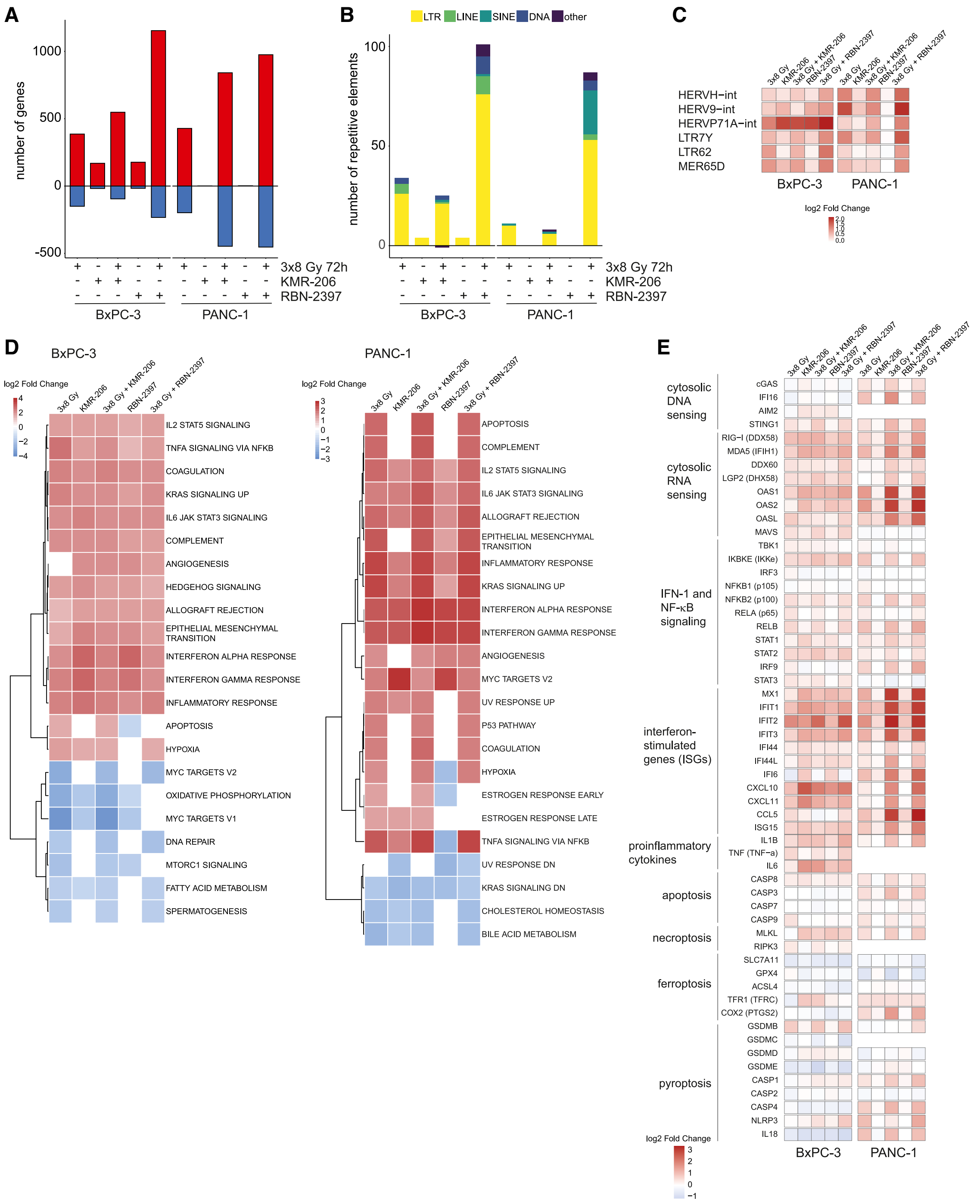

Figure 3. Transcriptome-wide induction of inflammatory response after radiation and PARP7 inhibition in PDAC cells

(A, B) Quantification of differentially expressed (A) genes or (B) repetitive elements (fold-change>2, $p < 0.05$) in BxPC-3 and PANC-1 cells treated with 3×8 Gy without or with the PARP7 inhibitors KMR-206 (300 nM) and RBN-2397 (1 μM). PARP7 inhibitors were added 24 h before irradiation and kept until harvesting 72 h after the last irradiation. (A) Upregulated genes are shown in red, downregulated genes in blue. (B) Different categories of repetitive elements are shown with different colors. (C) Log2 fold changes in gene expression between indicated treated and control samples of most strongly upregulated repetitive elements. (D) Normalized enrichment scores (NESs) from gene set enrichment analysis (GSEA) for Hallmark gene sets (MSigDB) between indicated treatments and controls in PDAC cells. Shown are pathways significant in at least 3 comparisons with significance level $p < 0.05$ adjusted for multiple testing. (E) Log2 fold changes in gene expression between indicated treated and control samples of chosen genes involved in immune signaling or cell death pathways. The experiments were performed in three biological replicates.

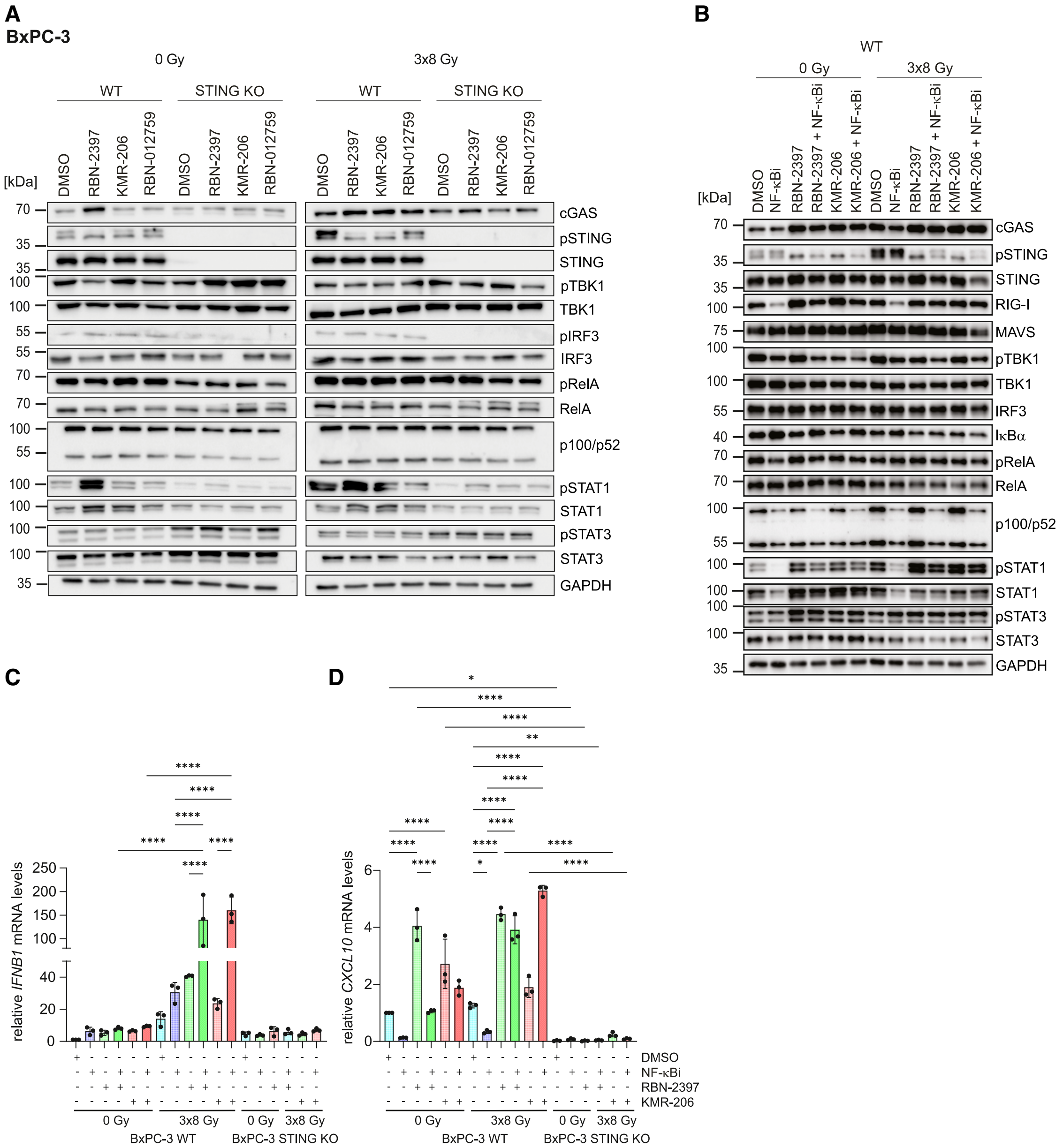

Figure 4. The type 1 interferon response induced by radiation and PARP7 inhibition is STING-dependent in BxPC-3 cells

(A, B) Western blot analysis of (A) WT and STING KO BxPC-3 cells treated with PARP7 and PARP14 inhibitors or (B) PARP7 inhibitors ± NF-κB inhibitor BI605906 without irradiation or with 3 × 8 Gy X-ray. Inhibitors were added 24 h before irradiation and kept until harvesting 72 h after the last fraction. GAPDH was used as a loading control. (C, D) RT-qPCR analysis of the expression of *IFNB1* and *CXCL10* in BxPC-3 cells. Gene expression was normalized to TBP. (A, B) The experiments were performed in two biological replicates. (C, D) The experiments were performed in three biological replicates. Data points represent mean values ±standard deviation. One-way ANOVA with Tukey's post hoc test was performed to determine significance (*≤ 0.05; **≤ 0.01; ***≤ 0.005, ****≤0.0001).

Immunogenic signaling induced by PARP7i is NF-κB- and STING-dependent in irradiated PANC-1 cells

In STING KO PANC-1 cells, pIRF3, pTBK1, pSTAT1, *IFNB1*, and *CXCL10* were severely reduced, indicating that STING regulates radiation- and PARP7i-induced IFN-I response (Figure 5). In MAVS KO PANC-1 cells, pSTING and pSTAT1 were not upregulated after radiation but showed partial induction when 3 × 8 Gy was combined with PARP7i (Figures S5A and S5B). As a result, the expression of *IFNB1* was reduced, but not abrogated, while the expression of *CXCL10* was unaffected in PANC-1 MAVS KO cells treated with 3 × 8 Gy and PARP7i (Figures S5C and S5D). Compared to STING and MAVS KO, NF-κB inhibition showed the strongest

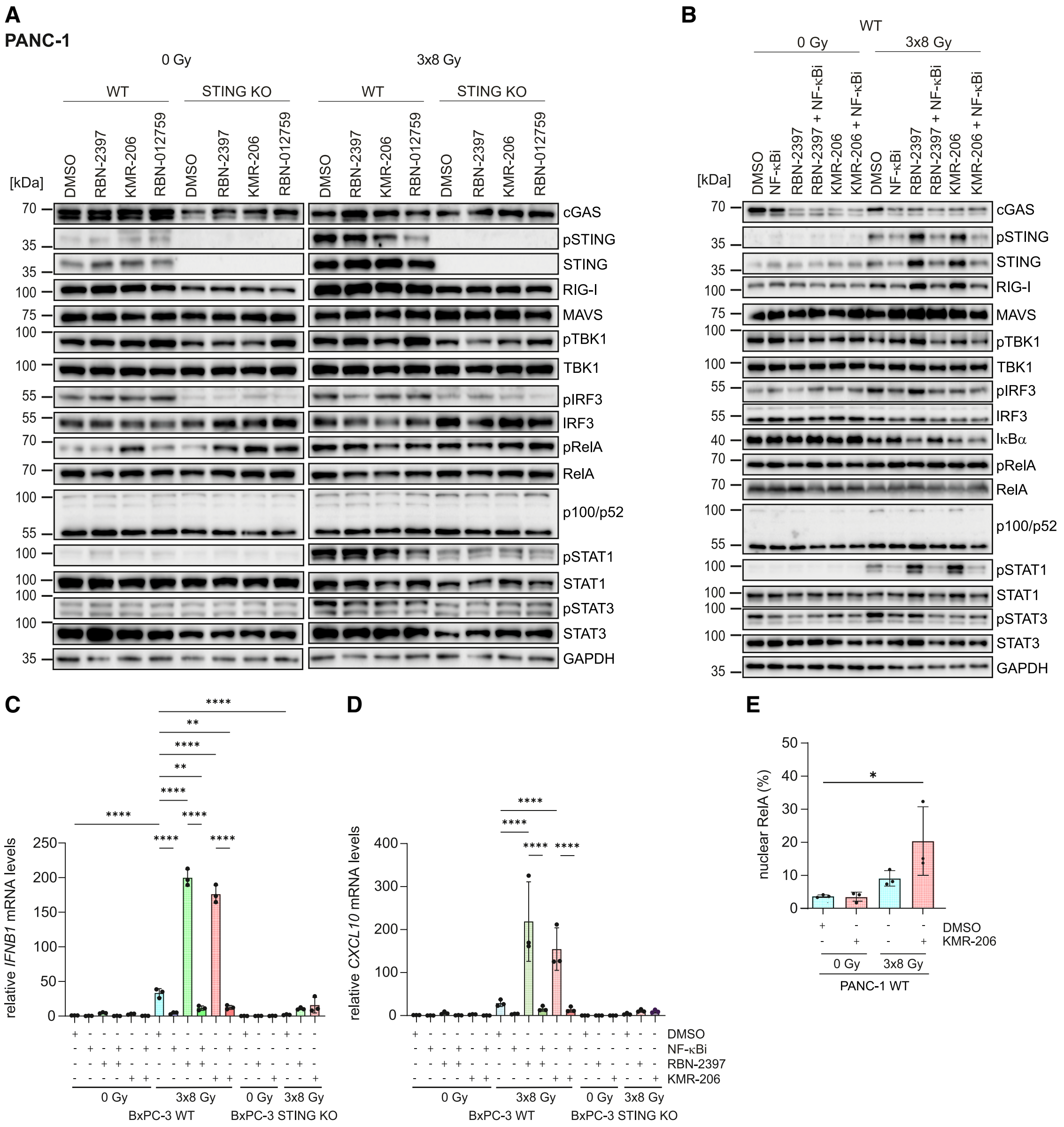

Figure 5. The type 1 interferon response induced by radiation and PARP7 inhibition is STING-and NF-κB-dependent in PANC-1 cells

(A, B) Western blot analysis of (A) WT and STING KO PANC-1 cells treated with PARP7 and PARP14 inhibitors or (B) PARP7 inhibitors ± NF-κB inhibitor BI605906 without irradiation or with 3 × 8 Gy X-ray. Inhibitors were added 24 h before irradiation and kept until harvesting 72 h after the last fraction. GAPDH was used as a loading control. (C, D) RT-qPCR analysis of the expression of *IFNB1* and *CXCL10* in PANC-1 cells. Gene expression was normalized to TBP. (E) Flow cytometry analysis of nuclear RelA in PANC-1 WT cells exposed to KMR-206, 3 × 8 Gy or their combination. (A, B) The experiments were performed in two biological replicates. (C–E) The experiments were performed in three biological replicates. Data points represent mean values ±standard deviation. One-way ANOVA with Tukey's post hoc test was performed to determine significance (*≤ 0.05; **≤ 0.01; ***≤ 0.005, ****≤0.0001).

reduction of pSTING, pSTAT1, *IFNB1*, and *CXCL10* expression (Figure 5).

To further test whether PARP7 inhibition induces NF-κB signaling, we performed flow cytometry-based analysis of nuclear NF-κB subunit RelA (p65) in PANC-1 cells. PANC-1 cells were treated with the PARP7i KMR-206, hypofractionated X-ray irradiation (3 × 8 Gy) or a combination thereof. The combination of PARP7i and radiation resulted in an increase in nuclear RelA (Figures 5E and S6), indicating a synergistic activation of the NF-κB pathway upon combinatorial treatment. Overall, these results suggest that the immunogenic effects of PARP7i combined

with radiation are primarily induced via NF-κB and STING pathways in PANC-1 cells.

Cell death pathways contribute to the immunogenic effects of radiation and PARP7i

To explore the functional outcome of immunogenic signaling induced by radiation and PARP7i, we examined the contribution of cell death pathways, release of DAMPs and cytokines, as well as activation of immune cells. Cell death pathways can exert either pro- or anti-immunogenic effects. Apoptosis suppresses the IFN-I response through caspase-mediated cleavage of cGAS, MAVS, and IRF3.[36,37] In contrast, necroptosis is strongly immunogenic due to membrane rupture and release of DAMPs.[38] Ferroptosis can exhibit both pro- and anti-immunogenic effects.[39] We pre-treated PDAC cell lines with the apoptosis inhibitor Q-VD-Oph, necroptosis inhibitor necrostatin-1, or ferroptosis inhibitor liproxstatin-1, and exposed them to 3 × 8 Gy radiation. Apoptosis inhibition resulted in upregulation of pSTAT1, *IFNB1*, and *CXCL10* in both cell lines (Figure S7), which is in line with the known anti-immunogenic effects of apoptosis.[36,37] In PANC-1 cells, necrostatin-1 dampened radiation-induced *CXCL10* (Figure S7). The combined treatment with PARP7i, radiation, and necrostatin-1 reduced pSTAT1, *IFNB1*, and *CXCL10* (Figure S8). Necrostatin-1 inhibits RIPK1, a key component of the necrosome complex formed with RIPK3.[40] Within this complex, RIPK3 phosphorylates MLKL, triggering its oligomerization and translocation to the plasma membrane, where it disrupts membrane integrity.[38] However, necrostatin-1 did not reduce pMLKL (Figure S8), suggesting that its immunosuppressive effects are RIPK1-independent. Indeed, necrostatin-1 was shown to inhibit TNF-induced necrosis in a RIPK1-independent manner.[41]

Given that immunogenic cell death pathways were shown to induce the release of DAMPs such as HMGB1 and ATP that can activate immune cells,[6] we measured ATP in the supernatant of BxPC-3 and PANC-1 cells. In both cell lines, ATP release was increased after radiation (Figures S9A and S9B). In BxPC-3 cells, PARP7i enhanced the effects of radiation, which was not the case for PANC-1 cells (Figures S9A and S9B).

Immunogenic extracellular ATP can be degraded to immunosuppressive adenosine, which is a well-known molecule creating an immunosuppressive environment in PDAC.[42] The ecto-nucleoside triphosphate diphosphohydrolase 1 (CD39/ENTPD1) and the ecto-nucleotide pyrophosphatase/phosphodiesterase 1 (ENPP1) convert ATP to AMP, which is further degraded to adenosine by the ecto-5′-nucleotidase (CD73/NT5E).[43–45] Interestingly, the expression of ATP degradation genes *CD39*, *ENPP1*, and *CD73* was higher in PANC-1 compared to BxPC-3, while CD39 and CD73 levels were increased after radiation in PANC-1 (Figure S9C). This pattern suggests that extracellular ATP, which is typically released during radiation-induced cell death and can act as an immunostimulatory signal, may be rapidly hydrolyzed into adenosine in PANC-1 cells. As adenosine is a potent immunosuppressive metabolite, its increased production could counteract ATP-mediated immune activation and promote an immunosuppressive TME. This may represent a mechanism by which PANC-1 cells limit the immunogenic effects of radiotherapy.

PARP7i potentiate the release of cytokines from irradiated PDAC cells

We further assessed whether transcriptional induction of proinflammatory cytokines is accompanied by their release from cancer cells. To this end, we performed IFNB1 ELISA and multiplex ELISA assay using LEGENDplex essential immune response panel to detect proinflammatory cytokines CXCL10, IL-6, IL-8, TNF-α, MCP-1 and anti-inflammatory cytokines IL-4 and IL-10 in supernatants from PDAC cells exposed to radiation and PARP7i. Irradiation with 3 × 8 Gy induced the release of all measured cytokines in BxPC-3, whereas only IFNB1 was induced in irradiated PANC-1 cells (Figure 6). PARP7i applied alone induced the release of IFNB1, CXCL10, IL-6, and MCP-1 in BxPC-3 cells, and acted synergistically with 3 × 8 Gy irradiation to enhance the release of IFNB1, CXCL10, IL-6, MCP-1, and IL-10 (Figure 6A), whereas in PANC-1 cells synergy was observed only for CXCL10 and IL-6 (Figure 6B). Importantly, the levels of these two cytokines measured in PANC-1 supernatants upon the combination treatment were lower than in the supernatants of similarly treated BxPC-3 cells (Figure 6). Overall, these results corroborate the RNA-seq and RT-qPCR analyses, demonstrating (1) immunogenic effects of PARP7i when applied alone in BxPC-3 cells, (2) synergistic effects with radiation for selected cytokines (IFNB1 and IL-6), but not CXCL10, in BxPC-3 cells, (3) immunogenic effects of PARP7i in PANC-1 cells only when combined with radiation, and (4) higher levels of cytokines in BxPC-3 supernatants compared to PANC-1. In PANC-1 cells, the enhanced cytokine gene expression observed upon combined PARP7 inhibition and irradiation was reflected at the protein level for CXCL10 and IL-6, but not for IFNB1.

Immune cell activation by BxPC-3 cells exposed to radiation and PARP7i

To measure the functional outcome of immunogenic effects of radiation and PARP7i in PDAC cells, we incubated peripheral blood mononuclear cells (PBMCs) with the supernatant of BxPC-3 and PANC-1 cells exposed to 3 × 8 Gy without or with PARP7i, followed by the PBMC analysis using spectral flow cytometry (Figures 7, S10, S11, and S12). Lineage markers (Table S3) enabled us to distinguish monocytes, NK cells, natural killer T (NKT) cells, CD4+ and CD8+ T cells, and B cells (Figure S10), while additional well-known activation-induced surface markers allowed us to assess their activation state.[46,47] For monocytes, we evaluated the co-stimulatory ligands CD80, CD86, and CD25, all of which are involved in antigen presentation and T cell activation. Activation of NK cells, NKT cells, CD4+, and CD8+ T cells was scored using early to intermediate activation markers CD69, CD25, and CD38, while activation of B cells was assessed using CD86, CD69, and CD38. Supernatants from irradiated BxPC-3 cells showed immunostimulatory effects by inducing the expression of CD25 and CD86 on monocytes, as well as CD69 and CD38 on NK cells, which was further increased in combination

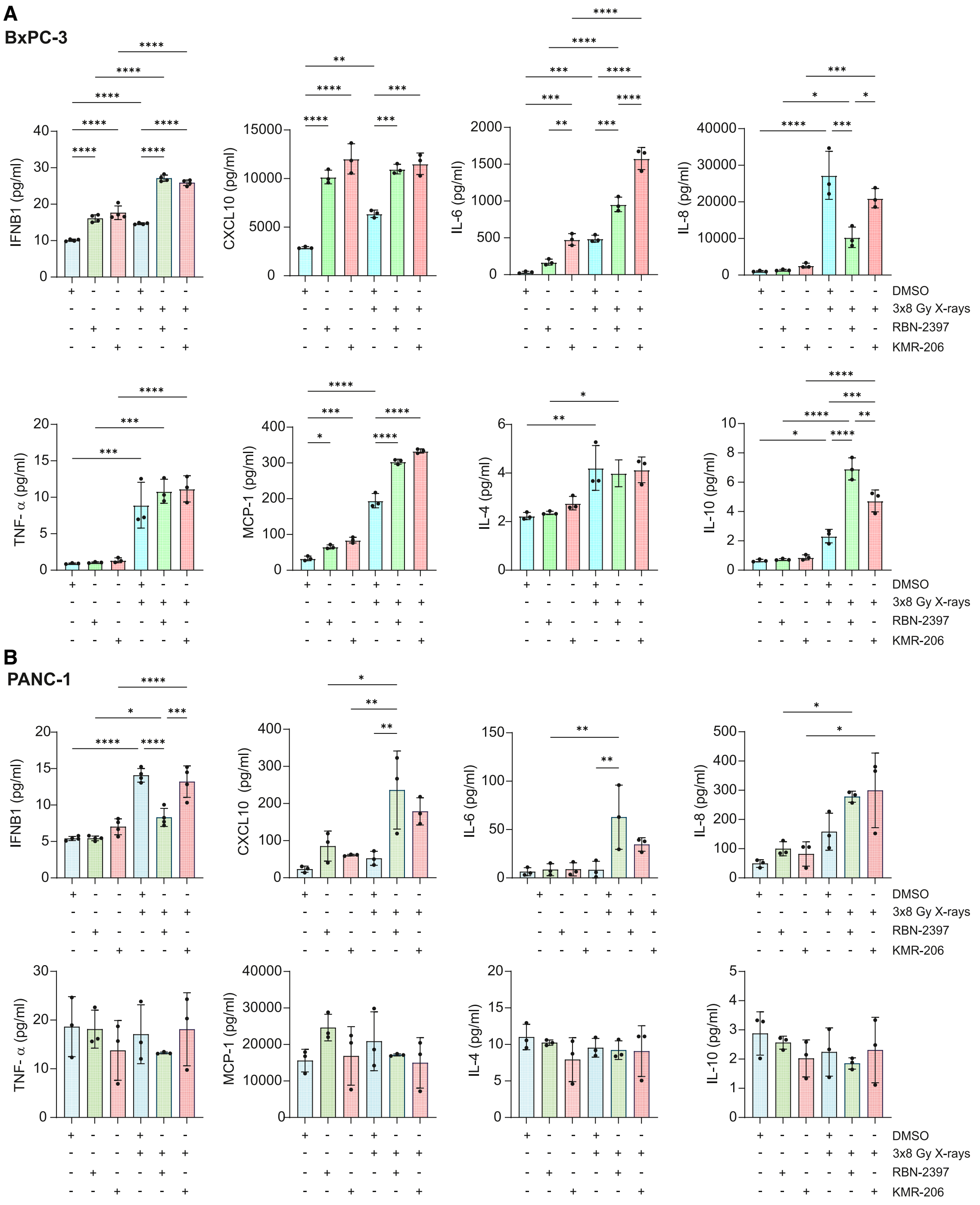

Figure 6. Release of pro-inflammatory cytokines by PDAC cells exposed to radiation and PARP7 inhibition

(A) BxPC-3 and (B) PANC-1 cells were treated with 3×8 Gy without or with the PARP7 inhibitors RBN-2397 (1 μM) and KMR-206 (300 nM). PARP7 inhibitors were added 24 h before irradiation and kept until the measurements 72 h after the last irradiation. Supernatants were tested by ELISA for IFNB1 (PBL Assay Science) and LEGENDplex Essential Immune Response Panel (CXCL10, IL-6, IL-8, TNF-α, MCP-1, IL-4, IL-10). The experiments were performed in three biological replicates. Data points represent mean values ±standard deviation. One-way ANOVA with Tukey's post hoc test was performed to determine significance (*$\leq$ 0.05; **$\leq$ 0.01; ***$\leq$ 0.005, ****$\leq$0.0001).

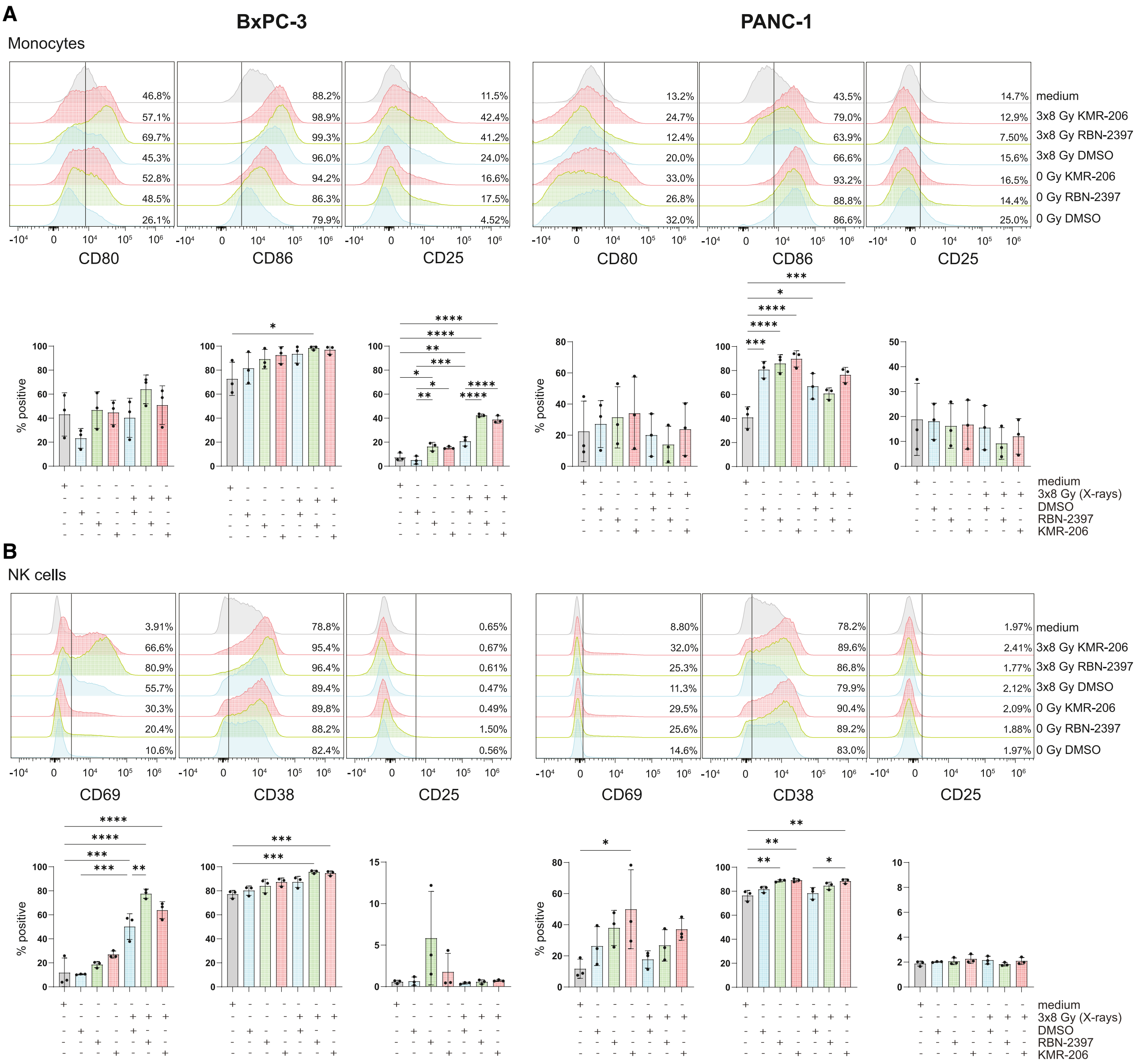

Figure 7. Response of primary human monocytes and NK cells to supernatants from PDAC cells treated with radiation and PARP7i

PBMCs from three different donors were incubated for 44 h in the presence of the culture medium or cell-free supernatants from BxPC-3 and PANC-1 cells treated with 3×8 Gy X-rays without or with the PARP7 inhibitors RBN-2397 (1 μM) or KMR-206 (300 nM). PARP7i were added 24 h before irradiation and kept until the measurements 72 h after the last irradiation. Human (A) monocytes and (B) NK cells were identified according to lineage markers and their activation was assessed based on the analysis of activation markers CD25, CD80, and CD86 (monocytes) or CD25, CD38, and CD69 (NK cells) by spectral flow cytometry. Representative histograms of one donor (top) and percentages of marker-positive cells ±standard deviation of three PBMC donors (bottom) are shown. Statistical significance was assessed using one-way ANOVA with Tukey's post hoc test (*≤ 0.05; **≤ 0.01; ***≤ 0.005, ****≤0.0001).

with PARP7i (Figures 7A and 7B). Activation of NKT and CD8+ T cells based on CD69 was not statistically significant (Figure S11). In contrast, supernatants from irradiated PANC-1 cells showed immunosuppressive effects based on the reduction in CD86 on monocytes (Figure 7A). PANC-1 supernatants treated with PARP7i alone increased the expression of CD38 and CD69 on NK cells (Figure 7B). Collectively, these results reveal that BxPC-3 cells exposed to radiation and PARP7i can activate monocytes and NK cells, while PARP7i-treated PANC-1 cells show immunostimulatory effects on NK cells.

DISCUSSION

In this work we showcase the cytotoxic and immunogenic cytotoxic effects of PARP7i in PDAC cells in combination with radiation. Furthermore, we elucidated the mechanistic basis of immunostimulatory effects induced by PARP7i and radiation as well as the differential response of PDAC cell lines relative to the expression levels of *PARP7*, *AHR*, interferon response genes, and KRAS mutation.

PARP7 was previously shown to regulate cell proliferation by ADP-ribosylating α-tubulin, resulting in microtubule instability.[48] PARP7 depletion reduces proliferation of OVCAR4 ovarian cancer cells but increases proliferation of MCF-7 breast cancer cells.[21,49] We found that both PARP7i reduce viability of PDAC cells, whereby BxPC-3 are more sensitive than PANC-1 (Figure 1). A higher sensitivity of BxPC-3 cells likely results from higher expression levels of PARP7, AHR, and interferon response genes compared to PANC-1 (Figure 1), which can be used as a biomarker for PARP7i sensitivity.

A CRISPR screen recently showed that PARP7 KO or inhibition elicits synthetic lethal interactions with a KO in genes required for chromatin remodeling (KDM5C and KDM6A), DNA damage response (CHEK2, NBN, RAD17, MSH2) and cell cycle regulation (TP53, CDKN2A, RB1).[32] Pancreatic cancer cell lines with high levels of chromatin remodeling factors (KDM5C and KDM6A), DNA damage signaling, and repair factors (ATR, ATM, DNA-PK, NBN, RAD50, BRCA1, BRCA2), mismatch repair genes (MSH2, MSH6, MLH1) and cohesion regulators (SMC1A, SMC3, WAPL) are not sensitive to PARP7i[32] (Figure S13A). Moreover, PAAD tumors show a positive correlation between the expression of PARP7 and these factors (Figure S13B), suggesting that dual inhibition of PARP7 and these factors may induce synthetic lethality in PDAC.

Both PARP7i exhibited comparable immunogenic effects in PDAC cells, but differed in their cytotoxicity: RBN-2397 showed greater efficacy as a monotherapy, whereas KMR-206 demonstrated stronger radiosensitizing properties (Figure 1). RBN-2397 and KMR-206 were previously also shown to exert different effects in colorectal (CT-26) and non-small cell lung carcinoma cells (NCI-H1373), with RBN-2397 causing a stronger reduction in viability of NCI-H1373, a stronger induction of IFNB1 in CT-26, and a stronger stabilization of PARP7 protein levels in CT-26.[19] KMR-206 is more selective against PARP2, but less selective against PARP10 and PARP11 *in vitro*.[19] However, to date PARP7i off-target effects have not been examined in cells.

In BxPC-3 cells, the induction of IFN-I response and proinflammatory cytokines is primarily driven by STING signaling (Figures 4). In this cell line, PARP7i reduced STING phosphorylation without having an effect on STING protein levels. Antibody against STING pS366, which promotes IRF3 binding and activation, detects two bands, of which the upper one was lost upon PARP7i treatment. We hypothesize that this additional phosphorylation site, distinct from pS366 but detected by the anti-pS366 antibody due to co-occurrence, exerts a negative regulatory effect on STING signaling. These findings suggest that PARP7i-mediated activation of STING signaling occurs through the removal of inhibitory phosphorylation, rather than through the canonical activation-associated phosphorylation followed by STING degradation. This possibility will be investigated in future studies.

In BxPC-3 cells, radiation strongly induces non-canonical NF-κB signaling, which dampens the IFN-I response. NF-κB inhibition also suppressed the non-canonical NF-κB pathway, and thereby enhanced *IFNB1* expression and pSTAT1 signaling. This was not observed in KRAS-mutated PANC-1 cells, where NF-κB inhibition completely abrogated IFN-I and NF-κB pathways induced by PARP7i and radiation (Figure 5), indicating that the immunogenic effects of radiation and PARP7i are primarily driven by the NF-κB pathway in PANC-1. This is not surprising considering that oncogenic KRAS mutations hyperactivate NF-κB signaling.[50]

Hypofractionated radiation induced the expression of transposable elements (ERV/LTR), which was potentiated by RBN-2397 but not KMR-206 (Figure 3B). Transposable elements (TE) make up approximately 10% of the human genome. Their expression can be induced by radiation, leading to recognition by cytosolic dsRNA sensors (RIG-I, MDA-5, OAS1, OAS2, OASL), which signal via MAVS to induce the IFN-I response.[51,52] ERV activation in PDAC patients is clinically associated with better disease outcomes.[53] The enhanced TE induction observed with radiation and RBN-2397, together with the diminished IFN-I response in MAVS KO cells, underscores the importance of this pathway in mediating IFN-I signaling through viral mimicry.

We did not observe immunogenic effects of PARP14 inhibition alone in PDAC cells (Figures S1 and S2), despite previous reports that PARP14 acts as a negative regulator of the IFN-I response by ADP-ribosylating STAT1 and thereby preventing its phosphorylation[54] and that the PARP14 inhibitor can promote the polarization of the pro-tumor M2 to anti-tumor M1 macrophages in melanomas.[27,28] More recent findings that PARP14i does not impair IFNγ and NF-κB signaling in A549 lung cancer cells[55] and does not increase pSTAT1 in U2OS osteosarcoma cells[56] align with our findings. In conclusion, PARP14i may have cell line-specific effects.

Despite the immunogenic effects of PARP7i combined with radiation in KRAS-mutated PANC-1 cells, as indicated by transcriptional upregulation of *IFNB1* and ISGs (Figures 2, 3, and 5), IFNB1 secretion was reduced with RBN-2397 or unchanged with KMR-206 in combination treatments compared to radiation alone (Figure 6). Moreover, ATP release as a marker of immunogenic cell death was not enhanced in the presence of PARP7i (Figure S9), and immune cells were not activated following incubation with supernatants from radiation-PARP7i-treated PANC-1 cells (Figure 7). The immunosuppressive effects of radiation and PARP7i in PANC-1 cells may result from degradation of immunogenic ATP to immunosuppressive adenosine (Figure S9C), which promotes M2 polarization of macrophages and suppresses IFNγ production by T cells.[42] The

expression of *CD73* (*NT5E*) is higher whereas the expression of *ENPP1* is lower in KRAS-mutated PAAD tumors compared to KRAS WT (Figure S9D). Radiation was shown to increase the production of adenosine,[57] which may explain the immunosuppressive effects of radiation in KRAS-mutated tumors. ENPP1 is an emerging drug target in the context of cGAS-STING signaling as it was shown to degrade extracellular cGAMP and dampen the immunogenic effects of radiation.[58] ENPP1 inhibitors increase cGAMP concentration, immune infiltration, and anti-tumor immunity in combination with radiation in breast and pancreatic cancer mouse models.[58] ENPP1 inhibitors could therefore synergize with PARP7i in KRAS-mutated PDAC.

In contrast to KRAS-mutated PANC-1 cells, KRAS WT BxPC-3 cells induced a strong IFN-I response when PARP7i were combined with radiation, as evidenced by the release of IFNB1 and ATP, ultimately leading to immune cell activation (Figures 2–4, 6, 7, and S9).

PARP7 is highly expressed in PDAC patients and its expression levels correlate with worse survival,[29] suggesting that PARP7 targeting with specific inhibitors is a promising strategy for PDAC tumors. Based on our findings, we propose an approach to induce anti-tumor immune response with PARP7i applied alone or in combination with radiotherapy. The cytotoxic effects of PARP7i depend on the expression levels of *PARP7*, *AHR*, and interferon response genes. Its immunogenic effects, however, may be attenuated in KRAS-mutated tumors and when combined with radiation, potentially due to adenosine accumulation. Consequently, in KRAS WT PDAC cells with high expression of *PARP7* and interferon response genes, PARP7i are expected to synergize with radiation to enhance both cytotoxic and immunogenic responses. In contrast, in KRAS-mutated cells, PARP7i are likely to be more effective as monotherapy. Moreover, our results support the use of hypofractionated C-ion irradiation in PDAC patients given the stronger immunogenic effects in combination with PARP7i compared to conventional X-ray irradiation (Figure 2) and the normal tissue-sparing properties of C-ion irradiation.[15] This study was limited to PDAC cell lines; therefore, further investigation of the effect of PARP7 inhibition will necessitate a PDAC mouse model as a step closer toward *in vivo* implementation of our *in vitro* findings.

MATERIALS AND METHODS

Cell culture

The human pancreatic cancer cell lines PANC-1 and BxPC-3 were purchased from ATCC. PANC-1 has a G12D mutation and is classified as mesenchymal. PANC-1 was cultured in Dulbecco's modified Eagle's medium (DMEM) medium (Gibco, 41965-039), supplemented with 10% fetal bovine serum (FBS) (Sigma, F7524-500 mL) and 1% penicillin/streptomycin (P/S) (Gibco, 15140-122). BxPC-3 was cultured in Roswell Park Memorial Institute (RPMI) 1640 medium (Gibco, 21875-034), supplemented with 10% FBS, 1% P/S and 1% L-Glutamine (Gibco, 25030-024). STING and MAVS KO cell lines were previously generated by CRISPR/Cas9.[14] All cell lines were cultured at 37°C in a humidified environment containing 95% air and 5% CO_2. Cells reached 70%–80% confluency at the time of irradiation. Before irradiation, culture flasks were filled without air bubbles using unsupplemented medium to allow vertical positioning during both irradiation workflows. Immediately after irradiation, this medium was replaced with fresh, supplemented medium.

Inhibitor treatments

For all experiments except for clonogenic assays, the PARP7i RBN-2397 (1 μM; TargetMol Chemicals), the PARP7i KMR-206 (300 nM; Michael Cohen), the PARP14 inhibitor RBN-012759 (1 μM; TargetMol Chemicals), and the NF-κB inhibitor BI605906 (1 μM; TargetMol Chemicals) were added 24 h prior to irradiation and kept until harvesting 72 h after the last irradiation. For clonogenic assays, RBN-2397 and KMR-206 were added 24 h prior to irradiation and removed 24 h after irradiation. Apoptosis, necroptosis, and ferroptosis were inhibited using Q-VD-Oph (10 μM; TargetMol Chemicals), necrostatin-1 (10 μM; TargetMol Chemicals) and liproxstatin-1 (5 μM; TargetMol Chemicals), respectively. The inhibitors were added 1 h before irradiation and maintained until harvesting.

Irradiation procedures

X-ray irradiations were carried out using a dedicated irradiation cabinet with horizontal X-ray tube orientation (YXLON, TU32-D03, 20 mA, 5.5 FOC, filtration: 3 mm Be + 3 mm Al + 0.5 mm Cu), to enable identical experimental setups as with C-ion irradiations performed at the horizontal experimental beamline of MedAustron. Thus, systematic setup-related uncertainties can be ruled out in *in vitro* experiments. For the C-ion studies, custom-made cell holders were designed and dosimetrically validated to allow cell irradiation in water, in line with recommendations from a recent National Cancer Institute special panel.[59] The positioning uncertainty was estimated at 0.3 mm.[60] For both experimental configurations (ChamberFlask and T25 flasks), a spread-out Bragg peak (SOBP) with a width of 4 cm and a range at the 80% dose level (R80) of 10.6 cm, corresponding to a C-ion energy of 285 MeV/u, was used. The dose-averaged linear energy transfer (LETD) was calculated using GATE/Geant4-based Monte Carlo simulations that accounted for the beam nozzle of the MedAustron experimental beamline, including contributions from all primary and secondary particles. The scoring volume was defined as a cylindrical geometry with a diameter of approximately 80 mm. At a depth of 8 cm, corresponding to the cell position at the center of the SOBP, the LETD was determined to be 58 keV/μm.[61] Cells were exposed to a physical dose of 8 Gy using either 200 kV X-rays or C-ions. In addition, a fractionated regimen of 3×8 Gy X-rays and 3xRBE-adjusted dose of C-ions was applied. RBE_{10} of C-ions was calculated as a ratio of X-ray and C-ion dose required to kill 90% of the cells (D_{10}). RBE_{10} for C-ions, as determined from our cell survival curves, was 2.41 in BxPC-3 cells and 2.32 in PANC-1 cells.

Clonogenic survival assay

Clonogenic survival assays were performed after X-ray or C-ion irradiation. 2.5×10^5 cells were seeded in Nunc Lab-Tek ChamberFlasks (170920) 2 days prior to irradiation. The PARP7i RBN-2397 (1 μM)

and KMR-206 (300 nM) were added 1 day before irradiation. Cells were harvested immediately after irradiation, diluted with supplemented medium appropriate for the cell line and seeded on 6-well plates in previously established, cell line specific concentrations. The inhibitors were removed 1 day after irradiation. Following an incubation period of 12 days (PANC-1) or 16 days (BxPC-3), cells were fixed with 96% methanol and stained with 0.5% crystal violet solution. Colonies of more than 50 cells were regarded as surviving clones. Surviving fractions in relation to the plating efficiency of non-irradiated control samples were calculated for each value of the delivered physical dose in Gy. The mean and standard deviation result from a minimum of 4 independent experiments. The linear-quadratic (LQ) fit was performed using the uncertainties package (v.3.2.4) from the open source Python library, as well as the lmfit package (v.1.3.4) with a user-defined model. To exclude over-survival and negative RBE, the following constraints were applied: (1) alpha <1.0 and (2) beta >0.0.

Western blotting

Cell pellets were resuspended in lysis buffer [50 mM Tris-Cl, pH 7.4, 250 mM NaCl, 1% NP-40, 5 mM EDTA, 50 mM NaF, 1 mM Na_3VO_4, 1 mM PMSF, 1% protease and phosphatase inhibitor cocktail (Thermo Fisher Scientific, D12345), PhosStop (Merck, 4906837001)], lysed on ice for 20 min, followed by centrifugation at 4°C for 10 min. Protein concentration was determined using the Bradford assay. About 20 μg of cell lysates were loaded onto SDS-PAGE gels and transferred onto nitrocellulose membranes using transfer buffer (25 mM Tris, 192 mM glycine, 10% ethanol) at 35 V overnight. Membranes were blocked for 1 h and incubated with primary antibodies overnight at 4°C on a roller. After three 10-min washing steps in Tris-buffered saline (TBS)-T (0.1% Tween 20 in TBS), membranes were incubated with HRP-conjugated secondary antibodies for 2 h at 4°C. Chemiluminescent signals were detected using the ChemiDoc MP Imaging System (Bio-Rad), operated with Bio-Rad Image Lab Touch Software (v.2.3.0.07), and analyzed using Bio-Rad Image Lab Software (v.5.2.1). All antibodies are listed in Table S4.

RNA isolation, reverse transcription, and quantitative reverse-transcription PCR

Cell pellets were resuspended in 1 mL TRAzol reagent (Neo-biotech), followed by the addition of 200 μL chloroform (Applichem). Samples were vortexed and centrifuged at maximum speed (21,100 × g) for 15 min at 4°C. The aqueous phase was carefully transferred to a fresh tube and RNA was precipitated by adding 0.5 mL isopropanol. The RNA pellet was collected by centrifugation for 30 min at 4°C, washed with 1 mL of 75% ethanol, and centrifuged again for 10 min, dried and resuspended in 70 μL RNase-free water. For removal of genomic DNA, 20 μg RNA was treated with 40 U DNase I (Roche) for 30 min at 37°C, followed by phenol-chloroform extraction and ethanol precipitation. Subsequently 1 μg of RNA was reverse transcribed using LunaScript RT SuperMix (NEB). Afterwards, cDNA was diluted 1:5 in nuclease-free water. *IFNB1*, *CXCL10*, *IL6*, and *TNF* expression levels relative to *TBP* were quantified by multiplexing reverse-transcription PCR (RT-PCR) based on commercial primer sets (human *IFNB1* UniqueAssayID: qHsaCEP0054112, human *CXCL10* UniqueAssayID: qHsaCEP0053880, human *IL6* UniqueAssayID: qHsaCEP0051939, human *TNF* UniqueAssayID: qHsaCEP0040184, human *TBP* UniqueAssayID: qHsaCIP0036255). PCRs were run on a CFX Opus 96 machine (Bio-Rad) operated by the embedded CFX Maestro software (Bio-Rad) using iQ Multiplex Powermix (Bio-Rad). RT-PCR data were analyzed using the ΔΔCt method with TBP as a housekeeping gene.

RNA isolation and RNA-seq library preparation

Cells were harvested and counted, PDAC cells were mixed with 10% mouse embryonic fibroblasts (MEFs) as a spike-in control. Cell pellets were resuspended in 1 mL TRAzol reagent (Neo-biotech). About 200 μL chloroform (Applichem) was added, samples were mixed and centrifuged at 4°C maximum speed for 15 min. The upper phase was transferred to a new tube and subjected to isopropanol precipitation. 20 μg of RNA were treated with 40 U DNase I (Roche, 50-100-3290) at 37°C for 30 min and purified by phenol-chloroform extraction and ethanol precipitation. Polyadenylated RNA was enriched and RNA-seq libraries were prepared with NEBNext Poly(A) mRNA Magnetic Isolation Module (New England Biolabs E7490) and NEBNext Ultra-Express RNA Library Prep Kit for Illumina (New England Biolabs E3330) according to the manufacturer's instructions using 250 ng total RNA input. Sequencing was performed on an Illumina NovaSeq X instrument in readmode PE150 by the Next Generation Sequencing facility at Vienna BioCenter Core Facilities (VBCF).

RNA-seq data analysis

RNA-seq data was processed using nf-core/rnaseq pipeline.[62] Data were quantified using combined GRCh38/hg38, and the mm10 versions of the human and mouse spike-in transcriptome downloaded from the ENSEMBL database[63] using SALMON[64] with default parameters. Only reads uniquely mapping to combined human and mouse genome assemblies were quantified. The quantified data were processed using tximport[65] and the differential expression analysis was done using DESeq2.[66] Genes with less than 5 reads in all biological replicates of one condition were filtered out before the differential analysis. Genes were defined as differentially expressed if they had a minimum absolute log2 fold change of 1, and a Benjamini-Hochberg (BH) adjusted *p* value less than 0.05. Genes from Hallmark gene sets from Molecular Signatures Database (MSigDB)[67] were tested for enrichment among expressed genes using gene set enrichment analysis (GSEA) algorithm[68] implemented in clusterProfiler R package.[69] Gene sets with adjusted *p* value <0.05 were defined as enriched. For the analysis of the repetitive genomic elements, reads were first trimmed by Trim Galore 0.6.10 with default parameters and then aligned to the concatenated human (GRCh38/hg38) and mouse (mm10) genome index using STAR 2.7.11b[70] with parameters chosen to optimize alignment for quantification of transposable elements.[71]

–outFilterMultimapNmax 5000 –winAnchorMultimapNmax 5000 –seedSearchStartLmax 30 –alignTranscriptsPerReadNmax 30000

–alignWindowsPerReadNmax 30000 –alignTranscriptsPerWindowNmax 300 –seedPerReadNmax 3000 –seedPerWindowNmax 300 –seedNoneLociPerWindow 1000 –outFilterMultimapScoreRange 0 –outFilterMismatchNoverLmax 0.05 –sjdbScore 2.

Custom.gtf file was created from RepeatMasker (Smit, A. F. A., Hubley, R. & Green, P. RepeatMasker Open-4.0 (RepeatMasker, 2013–2015)) annotation: simple repeats and low complexity regions were removed, and repeat names (repName) values were set as gene_id. This.gtf was concatenated to ENSEMBL.gtf file and used for alignment quantification with featureCounts 2.0.8[72] with parameters: *-F GTF -M -O –fraction –verbose –primary -s 2*. Differential expression analysis was done using DESeq2 on all quantified genes and repeats with more than 5 reads in all biological replicates of one condition. RNA-seq data have been deposited in "ArrayExpress: E-MTAB-16549."

TCGA expression and mutation data

RSEM-normalized, log2-transformed expression values for 178 PAAD tumor samples from The Cancer Genome Atlas Program (TCGA)[73] were obtained from firebrowse.org. Tumors lacking mutation sequencing data were excluded. A tumor was classified as KRAS mutant if it carried at least one KRAS missense mutation, retaining 93 KRAS mutant and 26 KRAS WT samples. For each gene, Spearman's rank correlation coefficient (ρ) with PARP7 expression was computed across all 178 samples, and p values were adjusted for multiple testing using the BH method.

Cell line expression and drug sensitivity data

TPM-normalized expression values for 37 pancreatic cancer cell lines were obtained from Cancer Dependency Map (DepMap) Cell Model Passports.[74] RBN-2397 sensitivity was defined as 1—AUC of the dose-response curve for cell proliferation.[32] Spearman's correlations between gene expression and RBN-2397 sensitivity were computed and BH-adjusted across all tested genes. Cell lines were classified as KRAS mutant if carrying at least one KRAS missense mutation (n = 30 mutant, n = 7 WT).

ISG score

An ISG score was computed from variance-stabilized (VST, DESeq2) expression values of 14 ISGs (*IFI16*, *STING1*, *PARP14*, *OAS1*, *OAS2*, *OAS3*, *OASL*, *CXCL10*, *IFIT2*, *IFIT3*, *IFI44*, *IFI44L*, *PARP9*, *IFITM1*). Expression values for each gene were Z score normalized across samples, and the ISG score was defined as the mean Z score across all 14 genes per sample.

ELISA IFNB1

PANC-1 (5×10^5) and BxPC-3 (6×10^5) cells were seeded into T25 flasks 2 days prior to irradiation and treated with the inhibitors the next day. After 72 h of the last irradiation, supernatants were collected and IFNB1 levels were quantified using the Human IFN-Beta TCM ELISA, High Sensitivity kit (41415-1, PBL Assay Science). Photometric measurements were taken with a microplate reader and normalized to viable cells count assessed with a Varioskan Flash Microplate Reader (Thermo Scientific).

Cytokine profiling with LegendPlex multi-analyte flow assay kit

PANC-1 and BxPC-3 supernatants were collected, centrifuged 1 min at 200 g to remove debris and stored at 4°C. Buffers and bead solutions were prepared and all subsequent experimental steps were performed according to the "Human Essential Immune Response Panel (13-plex) with Filter Plate" Manual (BioLegend, 740929) together with a Vacuum Manifold (Thermo Fisher Scientific, MSVMHTS00) with the following minor changes and acquisition parameters determined by pre-conducted test runs: undiluted supernatant were used as samples, PE incubation time lowered to 20 min only for PANC-1 samples and resuspended beads (after step 10 of the protocol) were transfered to a V-bottom plate for acquisition to prevent leakage during shaking. Samples were analyzed on a Bio-Rad ZE5 Cell Analyzer with 488 laser for FSC/SSC, 561 nm laser/577/15 filter for PE and 640 nm laser/670/30 filter for antigen-presenting cell (APC) acquisition. Raw and PE beads were used for instrument set up and PMT values set to 488: FSC 348 and SSC 524, 561: PE 420 and 640: APC 461, respectively. Flow rate was held at 0.3 μL/s, 3,900 events were set as total gate limit.

Isolation of PBMCs

PBMCs were obtained from venous blood of three healthy human donors in accordance with the Declaration of Helsinki and approved by the Ethics Committee of the Medical University of Vienna (2001/2018). Healthy donors came from the thrombocyte donation at the Medical University of Vienna, Department of Transfusion Medicine and Cell Therapy, Vienna, Austria. They were: D1 (male, 35 years old), D2 (female, 24 years old), and D3 (male, 38 years old). Written informed consent was obtained from all participants. PBMCs were isolated by density gradient centrifugation (800 g without break for 20 min) using Lymphoprep (Axis-Shield), followed by two washes with Ca^{2+}/Mg^{2+}-free phosphate buffered saline (PBS). Cells were afterward portioned into 2×10^7 cell aliquots and cryopreserved in FBS with 10% DMSO. On the day of the stimulation, PBMCs were thawed and thoroughly washed with the RPMI medium. PBMCs were then divided into 4×10^6 cell aliquots, which were sedimented by centrifugation (350× g, 5 min) and directly resuspended in 2 mL of fresh, cell-free supernatants collected from PDAC cell lines 72 h after the last irradiation. As a control, PBMCs were resuspended in fresh, supplemented DMEM or RPMI medium. PBMCs were analyzed by spectral flow cytometry after 44 h of culturing at 37°C in a humidified atmosphere with 5% CO_2.

Spectral flow cytometry

PBMCs were harvested and detached from cell culture plates using ice-cold PBS with 1.5 mM EDTA. After pelleting by centrifugation (350× g, 5 min) and washing with PBS, cells were stained with the LIVE/DEAD Fixable Blue Dead Cell Stain Kit (Thermo Fisher Scientific, L23105, 1:500 final concentration) for 20 min at room temperature, followed by two washing steps with pre-cooled staining buffer (PBS containing 1% BSA and 0.02% NaN_3). Cells were resuspended

in Brilliant Stain Buffer (BD Biosciences, 566349) supplemented with 3.2 mg/mL human IgG (Beriglobin P; CSL Behring) and 20 μL/mL True-Stain Monocyte Blocker (BioLegend, 426102) and incubated for 30 min on ice. 2×10^6 cells were stained using the full immunophenotyping panel, which consisted of 21 mAbs against surface antigens prediluted in Brilliant Stain Buffer, as indicated in Table S3. In parallel, a control lineage panel consisting of 9 mAbs was used to stain 10^6 cells. Pre-diluted chemokine receptor mAbs against CCR7, CCR5, CXCR3, and TCRγδ mAb were added prior to the remaining 17 mAbs in order to enhance the staining. TCRγδ mAb was also applied earlier in the lineage panel. Cells were incubated for 30 min on ice in the complete mAb cocktail. Cytek FSP CompBeads (Cytek Biosciences, #B7-10011) or non-stained cells were used in parallel for single stains and controls for unmixing and autofluorescence subtraction. Following mAb staining, samples were washed two times with staining buffer and data were acquired immediately using a Cytek Aurora spectral flow cytometer equipped with SpectroFlo software (Cytek Biosciences). Data unmixing was performed with SpectroFlo software and further analysis was performed using FlowJo 10 software (BD Biosciences). In order to remove artifacts, all samples were first subjected to data cleanup using the PeacoQC plugin in FlowJo.[75] For the removal of debris and doubles, all samples were pre-gated by evaluating the scatter profiles, and subsequently gated for individual PBMC subsets in accordance with the literature[76] and displayed in Figure S10. Data from lineage control stain was used for setting the gates for individual activation markers, which allowed for evaluation of the percentage of activated cells within individual PBMC lineages in the full stain samples.

Nuclear RelA (NF-κB p65) staining

Cells were washed twice with 1×PBS, fixed and permeabilized using Fixation/Permeabilization Diluent and Concentrate (#00–5223, #00–5123, eBioscience) and Permeabilization buffer (#00–5523, eBioscience) before intracellular staining with APC-conjugated anti- NF-κB p65 (#653005, BioLegend) for 30 min at room temperature. Samples were then washed twice with permeabilization buffer and PBS sequentially before sorting on the Cytek Aurora CS Cell Sorter. Untreated cells (0 Gy) were used as negative controls.

ATP assay

To assess ATP release following irradiation and inhibitor treatment, PANC-1 (5×10^5 for T25; 2×10^5 for ChamberFlasks) and BxPC-3 (6×10^5 for T25; 3×10^5 for ChamberFlasks) cells were seeded into T25 flasks or Nunc Lab-Tek ChamberFlasks (170920) 2 days prior to irradiation. Immediately after irradiation, cells were trypsinized and counted. Subsequently, 3,000 cells per well were seeded into a 96-well plate. Around 72 h after the last irradiation, 100 μL of the supernatant was collected from each well and ATP release was quantified using the CellTiter-Glo 2.0 Cell Viability Assay (Promega). To measure viability, cells were fixed using methanol and stained with 0.5% crytal violet. Plates were incubated overnight with 150 μL of 1% SDS per well, and absorbance was measured at 590 nm using a microplate reader. ATP levels were normalized to cell viability and all irradiated samples were afterward normalized to the 0 Gy control. Three biological experiments with three technical replicates each were performed.

Statistical analysis

All experiments were performed in 3–4 independent biological replicates and statistical significance was determined using two-way analysis of variance (ANOVA) with multiple comparisons correction: $* \leq 0.05$; $** \leq 0.01$; $*** \leq 0.005$, $**** \leq 0.0001$.

DATA AND CODE AVAILABILITY

RNA-seq dataset was deposited in ”ArrayExpress: E-MTAB-16549.” The processed RNA-seq data are provided in Tables S1 and S2. Raw data including western blot replicates are provided in Table S5.

ACKNOWLEDGMENTS

This work was supported by the FWF doc.funds.connect PAIR “Pre-clinical ion beam therapy” (DFH 13), FWF Weave (FW774A0501), and the Technopol grant K3-F-730/003-2020. We thank the ministry of Education, Science, and Research is gratefully acknowledged for the financial support providing beam time at MedAustron.

AUTHOR CONTRIBUTIONS

N.B. performed and analyzed experiments in Figures 2, 4, 5, S4, and S5; A.B.D. performed and analyzed experiments in Figures 1, 2, 3, 7, S9, S10, S11, and S12; A.O.-R. performed and analyzed experiments in Figures 7, S10, S11, and S12; A.D. performed and analyzed experiments in Figures 2, 6, and S8, and analyzed experiments in Figures 7, S10, S11, and S12; P. Fischer performed and analyzed experiments in Figures 1, 3, 4, S1, and S2; F.H. performed database and RNA-seq analysis; L.-M.A. generated RNA-seq libraries; L.W. performed experiments in Figure 6; A.K. performed and analyzed experiments in Figure 5E; A.R. analyzed CFU experiments; S.K.-G. performed and analyzed experiments in Figures 2 and S7; S.B. performed the survival curve fitting analysis in Figure 1; P. Fossati., D.G., J.W., and K.P. proofread the manuscript; M.C. provided the PARP7i KMR-206; D.S. designed and supervised the study, and wrote the manuscript.

DECLARATION OF INTERESTS

K.P. has received speaker’s honoraria from Celgene, Amgen Inc., and Janssen Pharmaceuticals, consultancy fees from Celgene, Takeda, and Janssen Pharmaceuticals, and research support from Roche Pharmaceuticals.

SUPPLEMENTAL INFORMATION

Supplemental information can be found online at https://doi.org/10.1016/j.omton.2026.201258.

REFERENCES

1. Pylayeva-Gupta, Y., Lee, K.E., Hajdu, C.H., Miller, G., and Bar-Sagi, D. (2012). Oncogenic Kras-induced GM-CSF production promotes the development of pancreatic neoplasia. Cancer Cell *21*, 836–847. https://doi.org/10.1016/j.ccr.2012.04.024.

2. Bayne, L.J., Beatty, G.L., Jhala, N., Clark, C.E., Rhim, A.D., Stanger, B.Z., and Vonderheide, R.H. (2012). Tumor-derived granulocyte-macrophage colony-stimulating factor regulates myeloid inflammation and T cell immunity in pancreatic cancer. Cancer Cell *21*, 822–835. https://doi.org/10.1016/j.ccr.2012.04.025.

3. Cheng, H., Fan, K., Luo, G., Fan, Z., Yang, C., Huang, Q., Jin, K., Xu, J., Yu, X., and Liu, C. (2019). Kras(G12D) mutation contributes to regulatory T cell conversion through activation of the MEK/ERK pathway in pancreatic cancer. Cancer Lett. *446*, 103–111. https://doi.org/10.1016/j.canlet.2019.01.013.

4. Balachandran, V.P., Łuksza, M., Zhao, J.N., Makarov, V., Moral, J.A., Remark, R., Herbst, B., Askan, G., Bhanot, U., Senbabaoglu, Y., et al. (2017). Identification of unique neoantigen qualities in long-term survivors of pancreatic cancer. Nature *551*, 512–516. https://doi.org/10.1038/nature24462.

5. Dongre, A., and Weinberg, R.A. (2021). Leveraging immunochemotherapy for treating pancreatic cancer. Cell Res. *31*, 1228–1229. https://doi.org/10.1038/s41422-021-00574-x.

6. Donlon, N.E., Power, R., Hayes, C., Reynolds, J.V., and Lysaght, J. (2021). Radiotherapy, immunotherapy, and the tumour microenvironment: Turning an immunosuppressive milieu into a therapeutic opportunity. Cancer Lett. *502*, 84–96. https://doi.org/10.1016/j.canlet.2020.12.045.

7. Balka, K.R., Louis, C., Saunders, T.L., Smith, A.M., Calleja, D.J., D'Silva, D.B., Moghaddas, F., Tailler, M., Lawlor, K.E., Zhan, Y., et al. (2020). TBK1 and IKKε Act Redundantly to Mediate STING-Induced NF-κB Responses in Myeloid Cells. Cell Rep. *31*, 107492. https://doi.org/10.1016/j.celrep.2020.03.056.

8. Ishikawa, H., and Barber, G.N. (2008). STING is an endoplasmic reticulum adaptor that facilitates innate immune signalling. Nature *455*, 674–678. https://doi.org/10.1038/nature07317.

9. Zhang, B.C., Pedersen, A., Reinert, L.S., Li, Y., Narita, R., Idorn, M., Hu, L., Skouboe, M.K., Li, S., Maimaitili, M., et al. (2025). STING signals to NF-κB from late endolysosomal compartments using IRF3 as an adaptor. Nat. Immunol. *26*, 1916–1930. https://doi.org/10.1038/s41590-025-02283-8.

10. Ablasser, A., and Hur, S. (2020). Regulation of cGAS- and RLR-mediated immunity to nucleic acids. Nat. Immunol. *21*, 17–29. https://doi.org/10.1038/s41590-019-0556-1.

11. Hopfner, K.-P., and Hornung, V. (2020). Molecular mechanisms and cellular functions of cGAS–STING signalling. Nat. Rev. Mol. Cell Biol. *21*, 501–521. https://doi.org/10.1038/s41580-020-0244-x.

12. Platanitis, E., Demiroz, D., Schneller, A., Fischer, K., Capelle, C., Hartl, M., Gossenreiter, T., Müller, M., Novatchkova, M., and Decker, T. (2019). A molecular switch from STAT2-IRF9 to ISGF3 underlies interferon-induced gene transcription. Nat. Commun. *10*, 2921. https://doi.org/10.1038/s41467-019-10970-y.

13. Liu, T., Zhang, L., Joo, D., and Sun, S.-C. (2017). NF-κB signaling in inflammation. Signal Transduct. Target. Ther. *2*, 17023. https://doi.org/10.1038/sigtrans.2017.23.

14. Kerschbaum-Gruber, S., Kleinwächter, A., Popova, K., Kneringer, A., Appel, L.M., Stasny, K., Röhrer, A., Dias, A.B., Benedum, J., Walch, L., et al. (2024). Cytosolic nucleic acid sensors and interferon beta-1 activation drive radiation-induced antitumour immune effects in human pancreatic cancer cells. Front. Immunol. *15*, 1286942. https://doi.org/10.3389/fimmu.2024.1286942.

15. Helm, A., Totis, C., Durante, M., and Fournier, C. (2023). Are charged particles a good match for combination with immunotherapy? Current knowledge and perspectives. Int Rev Cell Mol Biol *376*, 1–36. https://doi.org/10.1016/bs.ircmb.2023.01.001.

16. Ding, L., Kim, H.J., Wang, Q., Kearns, M., Jiang, T., Ohlson, C.E., Li, B.B., Xie, S., Liu, J.F., Stover, E.H., et al. (2018). PARP Inhibition Elicits STING-Dependent Antitumor Immunity in Brca1-Deficient Ovarian Cancer. Cell Rep. *25*, 2972–2980.e5. https://doi.org/10.1016/j.celrep.2018.11.054.

17. Pantelidou, C., Sonzogni, O., De Oliveria Taveira, M., Mehta, A.K., Kothari, A., Wang, D., Visal, T., Li, M.K., Pinto, J., Castrillon, J.A., et al. (2019). PARP Inhibitor Efficacy Depends on CD8(+) T-cell Recruitment via Intratumoral STING Pathway Activation in BRCA-Deficient Models of Triple-Negative Breast Cancer. Cancer Discov. *9*, 722–737. https://doi.org/10.1158/2159-8290.Cd-18-1218.

18. Gozgit, J.M., Vasbinder, M.M., Abo, R.P., Kunii, K., Kuplast-Barr, K.G., Gui, B., Lu, A.Z., Molina, J.R., Minissale, E., Swinger, K.K., et al. (2021). PARP7 negatively regulates the type I interferon response in cancer cells and its inhibition triggers antitumor immunity. Cancer Cell *39*, 1214–1226.e10. https://doi.org/10.1016/j.ccell.2021.06.018.

19. Sanderson, D.J., Rodriguez, K.M., Bejan, D.S., Olafsen, N.E., Bohn, I.D., Kojic, A., Sundalam, S., Siordia, I.R., Duell, A.K., Deng, N., et al. (2023). Structurally distinct PARP7 inhibitors provide new insights into the function of PARP7 in regulating nucleic acid-sensing and IFN-β signaling. Cell Chem. Biol. *30*, 43–54.e8. https://doi.org/10.1016/j.chembiol.2022.11.012.

20. Yamada, T., Horimoto, H., Kameyama, T., Hayakawa, S., Yamato, H., Dazai, M., Takada, A., Kida, H., Bott, D., Zhou, A.C., et al. (2016). Constitutive aryl hydrocarbon receptor signaling constrains type I interferon-mediated antiviral innate defense. Nat. Immunol. *17*, 687–694. https://doi.org/10.1038/ni.3422.

21. Rasmussen, M., Alvik, K., Kannen, V., Olafsen, N.E., Erlingsson, L.A.M., Grimaldi, G., Takaoka, A., Grant, D.M., and Matthews, J. (2023). Loss of PARP7 Increases Type I Interferon Signaling in EO771 Breast Cancer Cells and Prevents Mammary Tumor Growth by Increasing Antitumor Immunity. Cancers (Basel) *15*, 3689. https://doi.org/10.3390/cancers15143689.

22. Manetsch, P., Böhi, F., Nowak, K., Leslie Pedrioli, D.M., and Hottiger, M.O. (2023). PARP7-mediated ADP-ribosylation of FRA1 promotes cancer cell growth by repressing IRF1- and IRF3-dependent apoptosis. Proc. Natl. Acad. Sci. USA *120*, e2309047120. https://doi.org/10.1073/pnas.2309047120.

23. Naulin, F., Guilbaud, E., and Galluzzi, L. (2024). PARP7 and nucleic acid-driven oncosuppression. Cell. Mol. Immunol. *21*, 1177–1179. https://doi.org/10.1038/s41423-024-01182-6.

24. Jeltema, D., Knox, K., Dobbs, N., Tang, Z., Xing, C., Araskiewicz, A., Yang, K., Siordia, I.R., Matthews, J., Cohen, M., and Yan, N. (2025). PARP7 inhibits type I interferon signaling to prevent autoimmunity and lung disease. J. Exp. Med. *222*, e20241184. https://doi.org/10.1084/jem.20241184.

25. Diani-Moore, S., Ram, P., Li, X., Mondal, P., Youn, D.Y., Sauve, A.A., and Rifkind, A. B. (2010). Identification of the aryl hydrocarbon receptor target gene TiPARP as a mediator of suppression of hepatic gluconeogenesis by 2,3,7,8-tetrachlorodibenzo-p-dioxin and of nicotinamide as a corrective agent for this effect. J. Biol. Chem. *285*, 38801–38810. https://doi.org/10.1074/jbc.M110.131573.

26. Kannen, V., Rasmussen, M., Das, S., Giuliana, P., Izzati, F.N., Choksi, H., Erlingsson, L.A.M., Olafsen, N.E., Åhrling, S.S., Cappello, P., et al. (2024). Loss of Parp7 increases type I interferon signalling and reduces pancreatic tumour growth by enhancing immune cell infiltration. Front. Immunol. *15*, 1513595. https://doi.org/10.3389/fimmu.2024.1513595.

27. Schenkel, L.B., Molina, J.R., Swinger, K.K., Abo, R., Blackwell, D.J., Lu, A.Z., Cheung, A.E., Church, W.D., Kunii, K., Kuplast-Barr, K.G., et al. (2021). A potent and selective PARP14 inhibitor decreases protumor macrophage gene expression and elicits inflammatory responses in tumor explants. Cell Chem. Biol. *28*, 1158–1168.e13. https://doi.org/10.1016/j.chembiol.2021.02.010.

28. Wong, C.W., Evangelou, C., Sefton, K.N., Leshem, R., Zhang, W., Gopalan, V., Chattrakarn, S., Fernandez Carro, M.L., Uzuner, E., Mole, H., et al. (2023). PARP14 inhibition restores PD-1 immune checkpoint inhibitor response following IFNγ-driven acquired resistance in preclinical cancer models. Nat. Commun. *14*, 5983. https://doi.org/10.1038/s41467-023-41737-1.

29. Popova, K., Benedum, J., Engl, M., Lütgendorf-Caucig, C., Fossati, P., Widder, J., Podar, K., and Slade, D. (2025). PARP7 as a new target for activating anti-tumor immunity in cancer. EMBO Mol. Med. *17*, 872–888. https://doi.org/10.1038/s44321-025-00214-6.

30. Yang, C., Wierbiłowicz, K., Dworak, N.M., Bae, S.Y., Tengse, S.B., Abianeh, N., Drake, J.M., Abbas, T., Ratan, A., Wotton, D., and Paschal, B.M. (2023). Induction of PARP7 Creates a Vulnerability for Growth Inhibition by RBN2397 in Prostate Cancer Cells. Cancer Res. Commun. *3*, 592–606. https://doi.org/10.1158/2767-9764.Crc-23-0086.

31. Chen, H., Diolaiti, M.E., O'Leary, P.C., Rojc, A., Krogan, N.J., Kim, M., and Ashworth, A. (2022). A Whole-Genome CRISPR Screen Identifies AHR Loss as a Mechanism of Resistance to a PARP7 Inhibitor. Mol. Cancer Ther. *21*, 1076–1089. https://doi.org/10.1158/1535-7163.Mct-21-0841.

32. Fong, S.H., Kuenzi, B.M., Mattson, N.M., Lee, J., Sanchez, K., Bojorquez-Gomez, A., Ford, K., Munson, B.P., Licon, K., Bergendahl, S., et al. (2025). A multilineage screen identifies actionable synthetic lethal interactions in human cancers. Nat. Genet. *57*, 154–164. https://doi.org/10.1038/s41588-024-01971-9.

33. Charpentier, M., Spada, S., Van Nest, S.J., and Demaria, S. (2022). Radiation therapy-induced remodeling of the tumor immune microenvironment. Semin. Cancer Biol. *86*, 737–747. https://doi.org/10.1016/j.semcancer.2022.04.003.

34. Clark, K., Peggie, M., Plater, L., Sorcek, R.J., Young, E.R.R., Madwed, J.B., Hough, J., McIver, E.G., and Cohen, P. (2011). Novel cross-talk within the IKK family controls innate immunity. Biochem. J. *434*, 93–104. https://doi.org/10.1042/bj20101701.

35. Jin, J., Hu, H., Li, H.S., Yu, J., Xiao, Y., Brittain, G.C., Zou, Q., Cheng, X., Mallette, F. A., Watowich, S.S., and Sun, S.C. (2014). Noncanonical NF-κB pathway controls the production of type I interferons in antiviral innate immunity. Immunity *40*, 342–354. https://doi.org/10.1016/j.immuni.2014.02.006.

36. Ning, X., Wang, Y., Jing, M., Sha, M., Lv, M., Gao, P., Zhang, R., Huang, X., Feng, J. M., and Jiang, Z. (2019). Apoptotic Caspases Suppress Type I Interferon Production via the Cleavage of cGAS, MAVS, and IRF3. Molecular cell *74*, 19–31.e7. https://doi.org/10.1016/j.molcel.2019.02.013.

37. White, M.J., McArthur, K., Metcalf, D., Lane, R.M., Cambier, J.C., Herold, M.J., van Delft, M.F., Bedoui, S., Lessene, G., Ritchie, M.E., et al. (2014). Apoptotic caspases suppress mtDNA-induced STING-mediated type I IFN production. Cell *159*, 1549–1562. https://doi.org/10.1016/j.cell.2014.11.036.

38. Meier, P., Legrand, A.J., Adam, D., and Silke, J. (2024). Immunogenic cell death in cancer: targeting necroptosis to induce antitumour immunity. Nat. Rev. Cancer *24*, 299–315. https://doi.org/10.1038/s41568-024-00674-x.

39. Chen, F., Kang, R., Tang, D., and Liu, J. (2024). Ferroptosis: principles and significance in health and disease. J. Hematol. Oncol. *17*, 41. https://doi.org/10.1186/s13045-024-01564-3.

40. Degterev, A., Hitomi, J., Germscheid, M., Ch'en, I.L., Korkina, O., Teng, X., Abbott, D., Cuny, G.D., Yuan, C., Wagner, G., et al. (2008). Identification of RIP1 kinase as a specific cellular target of necrostatins. Nat. Chem. Biol. *4*, 313–321. https://doi.org/10.1038/nchembio.83.

41. Cho, Y., McQuade, T., Zhang, H., Zhang, J., and Chan, F.K.M. (2011). RIP1-dependent and independent effects of necrostatin-1 in necrosis and T cell activation. PLoS One *6*, e23209. https://doi.org/10.1371/journal.pone.0023209.

42. Jacoberger-Foissac, C., Cousineau, I., Bareche, Y., Allard, D., Chrobak, P., Allard, B., Pommey, S., Messaoudi, N., McNicoll, Y., Soucy, G., et al. (2023). CD73 Inhibits cGAS-STING and Cooperates with CD39 to Promote Pancreatic Cancer. Cancer Immunol. Res. *11*, 56–71. https://doi.org/10.1158/2326-6066.Cir-22-0260.

43. Borza, R., Salgado-Polo, F., Moolenaar, W.H., and Perrakis, A. (2022). Structure and function of the ecto-nucleotide pyrophosphatase/phosphodiesterase (ENPP) family: Tidying up diversity. J. Biol. Chem. *298*, 101526. https://doi.org/10.1016/j.jbc.2021.101526.

44. Robson, S.C., Sévigny, J., and Zimmermann, H. (2006). The E-NTPDase family of ectonucleotidases: Structure function relationships and pathophysiological significance. Purinergic Signal. *2*, 409–430. https://doi.org/10.1007/s11302-006-9003-5.

45. Bach, N., Winzer, R., Tolosa, E., Fiedler, W., and Brauneck, F. (2023). The Clinical Significance of CD73 in Cancer. Int. J. Mol. Sci. *24*, 11759. https://doi.org/10.3390/ijms241411759.

46. Chulpanova, D.S., Kitaeva, K.V., Green, A.R., Rizvanov, A.A., and Solovyeva, V.V. (2020). Molecular Aspects and Future Perspectives of Cytokine-Based Anti-cancer Immunotherapy. Front. Cell Dev. Biol. *8*, 402. https://doi.org/10.3389/fcell.2020.00402.

47. Velten, F.W., Rambow, F., Metharom, P., and Goerdt, S. (2007). Enhanced T-cell activation and T-cell-dependent IL-2 production by CD83+, CD25high, CD43high human monocyte-derived dendritic cells. Mol. Immunol. *44*, 1544–1550. https://doi.org/10.1016/j.molimm.2006.08.020.

48. Palavalli Parsons, L.H., Challa, S., Gibson, B.A., Nandu, T., Stokes, M.S., Huang, D., Lea, J.S., and Kraus, W.L. (2021). Identification of PARP-7 substrates reveals a role for MARylation in microtubule control in ovarian cancer cells. eLife *10*, e60481. https://doi.org/10.7554/eLife.60481.

49. Rasmussen, M., Tan, S., Somisetty, V.S., Hutin, D., Olafsen, N.E., Moen, A., Anonsen, J.H., Grant, D.M., and Matthews, J. (2021). PARP7 and Mono-ADP-Ribosylation Negatively Regulate Estrogen Receptor α Signaling in Human Breast Cancer Cells. Cells *10*, 623. https://doi.org/10.3390/cells10030623.

50. Ling, J., Kang, Y., Zhao, R., Xia, Q., Lee, D.F., Chang, Z., Li, J., Peng, B., Fleming, J.B., Wang, H., et al. (2012). KrasG12D-induced IKK2/β/NF-κB activation by IL-1α and p62 feedforward loops is required for development of pancreatic ductal adenocarcinoma. Cancer Cell *21*, 105–120. https://doi.org/10.1016/j.ccr.2011.12.006.

51. Lee, A.K., Pan, D., Bao, X., Hu, M., Li, F., and Li, C.Y. (2020). Endogenous Retrovirus Activation as a Key Mechanism of Anti-Tumor Immune Response in Radiotherapy. Radiat. Res. *193*, 305–317. https://doi.org/10.1667/rade-20-00013.

52. Du, J., Kageyama, S.I., Yamashita, R., Tanaka, K., Okumura, M., Motegi, A., Hojo, H., Nakamura, M., Hirata, H., Sunakawa, H., et al. (2023). Transposable elements potentiate radiotherapy-induced cellular immune reactions via RIG-I-mediated virus-sensing pathways. Commun. Biol. *6*, 818. https://doi.org/10.1038/s42003-023-05080-x.

53. Parikh, A.R., Szabolcs, A., Allen, J.N., Clark, J.W., Wo, J.Y., Raabe, M., Thel, H., Hoyos, D., Mehta, A., Arshad, S., et al. (2021). Radiation therapy enhances immunotherapy response in microsatellite stable colorectal and pancreatic adenocarcinoma in a phase II trial. Nat. Cancer *2*, 1124–1135. https://doi.org/10.1038/s43018-021-00269-7.

54. Iwata, H., Goettsch, C., Sharma, A., Ricchiuto, P., Goh, W.W.B., Halu, A., Yamada, I., Yoshida, H., Hara, T., Wei, M., et al. (2016). PARP9 and PARP14 cross-regulate macrophage activation via STAT1 ADP-ribosylation. Nat. Commun. *7*, 12849. https://doi.org/10.1038/ncomms12849.

55. Raja, R., Biswas, B., Abraham, R., Wang, Y., Chang, C.Y., Hendriks, I.A., Buch-Larsen, S.C., Liu, H., Yang, X., Wang, C., et al. (2025). Interferon-induced PARP14-mediated ADP-ribosylation in p62 bodies requires the ubiquitin-proteasome system. EMBO J. *44*, 2741–2773. https://doi.org/10.1038/s44318-025-00421-4.

56. Kar, P., Chatrin, C., Đukić, N., Suyari, O., Schuller, M., Zhu, K., Prokhorova, E., Bigot, N., Baretić, D., Ahel, J., et al. (2024). PARP14 and PARP9/DTX3L regulate interferon-induced ADP-ribosylation. EMBO J. *43*, 2929–2953. https://doi.org/10.1038/s44318-024-00126-0.

57. Wirsdörfer, F., de Leve, S., Cappuccini, F., Eldh, T., Meyer, A.V., Gau, E., Thompson, L.F., Chen, N.Y., Karmouty-Quintana, H., Fischer, U., et al. (2016). Extracellular Adenosine Production by ecto-5'-Nucleotidase (CD73) Enhances Radiation-Induced Lung Fibrosis. Cancer Res. *76*, 3045–3056. https://doi.org/10.1158/0008-5472.Can-15-2310.

58. Carozza, J.A., Böhnert, V., Nguyen, K.C., Skariah, G., Shaw, K.E., Brown, J.A., Rafat, M., von Eyben, R., Graves, E.E., Glenn, J.S., et al. (2020). Extracellular cGAMP is a cancer cell-produced immunotransmitter involved in radiation-induced anti-cancer immunity. Nat. Cancer *1*, 184–196. https://doi.org/10.1038/s43018-020-0028-4.

59. Durante, M., Paganetti, H., Pompos, A., Kry, S.F., Wu, X., and Grosshans, D.R. (2019). Report of a National Cancer Institute special panel: Characterization of the physical parameters of particle beams for biological research. Med. Phys. *46*, e37–e52. https://doi.org/10.1002/mp.13324.

60. Barna, S., Resch, A.F., Puchalska, M., Georg, D., and Palmans, H. (2022). Technical note: Experimental determination of the effective point of measurement of the PTW-31010 ionization chamber in proton and carbon ion beams. Med. Phys. *49*, 675–681. https://doi.org/10.1002/mp.15377.

61. Fischer, P., Schmid, M., Ohradanova-Repic, A., Schneeweiss, R., Hadatsch, J., Grünert, O., Benedum, J., Röhrer, A., Staudinger, F., Schatzlmaier, P., et al. (2025). Molecular features of TNBC govern heterogeneity in the response to radiation and autophagy inhibition. Cell Death Dis. *16*, 540. https://doi.org/10.1038/s41419-025-07873-w.

62. Ewels, P.A., Peltzer, A., Fillinger, S., Patel, H., Alneberg, J., Wilm, A., Garcia, M.U., Di Tommaso, P., and Nahnsen, S. (2020). The nf-core framework for community-curated bioinformatics pipelines. Nat. Biotechnol. *38*, 276–278. https://doi.org/10.1038/s41587-020-0439-x.

63. Zerbino, D.R., Achuthan, P., Akanni, W., Amode, M.R., Barrell, D., Bhai, J., Billis, K., Cummins, C., Gall, A., Girón, C.G., et al. (2018). Ensembl 2018. Nucleic Acids Res. *46*, D754–D761. https://doi.org/10.1093/nar/gkx1098.

64. Patro, R., Duggal, G., Love, M.I., Irizarry, R.A., and Kingsford, C. (2017). Salmon provides fast and bias-aware quantification of transcript expression. Nat. Methods *14*, 417–419. https://doi.org/10.1038/nmeth.4197.

65. Soneson, C., Love, M.I., and Robinson, M.D. (2016). Differential analyses for RNA-seq: transcript-level estimates improve gene-level inferences. F1000Res. *4*, 1521. https://doi.org/10.12688/f1000research.7563.2.

66. Love, M.I., Huber, W., and Anders, S. (2014). Moderated estimation of fold change and dispersion for RNA-seq data with DESeq2. Genome Biol. *15*, 550. https://doi.org/10.1186/s13059-014-0550-8.

67. Liberzon, A., Birger, C., Thorvaldsdóttir, H., Ghandi, M., Mesirov, J.P., and Tamayo, P. (2015). The Molecular Signatures Database (MSigDB) hallmark gene set collection. Cell Syst. *1*, 417–425. https://doi.org/10.1016/j.cels.2015.12.004.

68. Subramanian, A., Tamayo, P., Mootha, V.K., Mukherjee, S., Ebert, B.L., Gillette, M. A., Paulovich, A., Pomeroy, S.L., Golub, T.R., Lander, E.S., and Mesirov, J.P. (2005). Gene set enrichment analysis: a knowledge-based approach for interpreting genome-wide expression profiles. Proc. Natl. Acad. Sci. USA *102*, 15545–15550. https://doi.org/10.1073/pnas.0506580102.

69. Yu, G., Wang, L.G., Han, Y., and He, Q.Y. (2012). clusterProfiler: an R package for comparing biological themes among gene clusters. OMICS *16*, 284–287. https://doi.org/10.1089/omi.2011.0118.

70. Dobin, A., Davis, C.A., Schlesinger, F., Drenkow, J., Zaleski, C., Jha, S., Batut, P., Chaisson, M., and Gingeras, T.R. (2013). STAR: ultrafast universal RNA-seq aligner. Bioinformatics (Oxford, England) *29*, 15–21. https://doi.org/10.1093/bioinformatics/bts635.

71. Teissandier, A., Servant, N., Barillot, E., and Bourc'his, D. (2019). Tools and best practices for retrotransposon analysis using high-throughput sequencing data. Mob. DNA *10*, 52. https://doi.org/10.1186/s13100-019-0192-1.

72. Liao, Y., Smyth, G.K., and Shi, W. (2014). featureCounts: an efficient general purpose program for assigning sequence reads to genomic features. Bioinformatics (Oxford, England) *30*, 923–930. https://doi.org/10.1093/bioinformatics/btt656.

73. (2017). Integrated Genomic Characterization of Pancreatic Ductal Adenocarcinoma. Cancer Cell *32*, 185–203.e113. https://doi.org/10.1016/j.ccell.2017.07.007.

74. Arafeh, R., Shibue, T., Dempster, J.M., Hahn, W.C., and Vazquez, F. (2025). The present and future of the Cancer Dependency Map. Nat. Rev. Cancer *25*, 59–73. https://doi.org/10.1038/s41568-024-00763-x.

75. Emmaneel, A., Quintelier, K., Sichien, D., Rybakowska, P., Marañón, C., Alarcón-Riquelme, M.E., Van Isterdael, G., Van Gassen, S., and Saeys, Y. (2022). PeacoQC: Peak-based selection of high quality cytometry data. Cytometry. A. *101*, 325–338. https://doi.org/10.1002/cyto.a.24501.

76. Preglej, T., Brinkmann, M., Steiner, G., Aletaha, D., Göschl, L., and Bonelli, M. (2023). Advanced immunophenotyping: A powerful tool for immune profiling, drug screening, and a personalized treatment approach. Front. Immunol. *14*, 1096096. https://doi.org/10.3389/fimmu.2023.1096096.

OMTON, Volume *34*

Supplemental information

PARP7 inhibitors enhance the immunogenic effects of radiation in pancreatic cancer cells

Niccolò Bragato, Ana Beatriz Dias, Anna Ohradanova-Repic, Alma Dupanovic, Filip Horvat, Patrick Fischer, Lisa-Marie Appel, Lena Walch, Ava Kleinwächter, Anna Röhrer, Sylvia Kerschbaum-Gruber, Sandra Barna, Piero Fossati, Dietmar Georg, Joachim Widder, Klaus Podar, Michael Cohen, and Dea Slade

Supplemental Figures

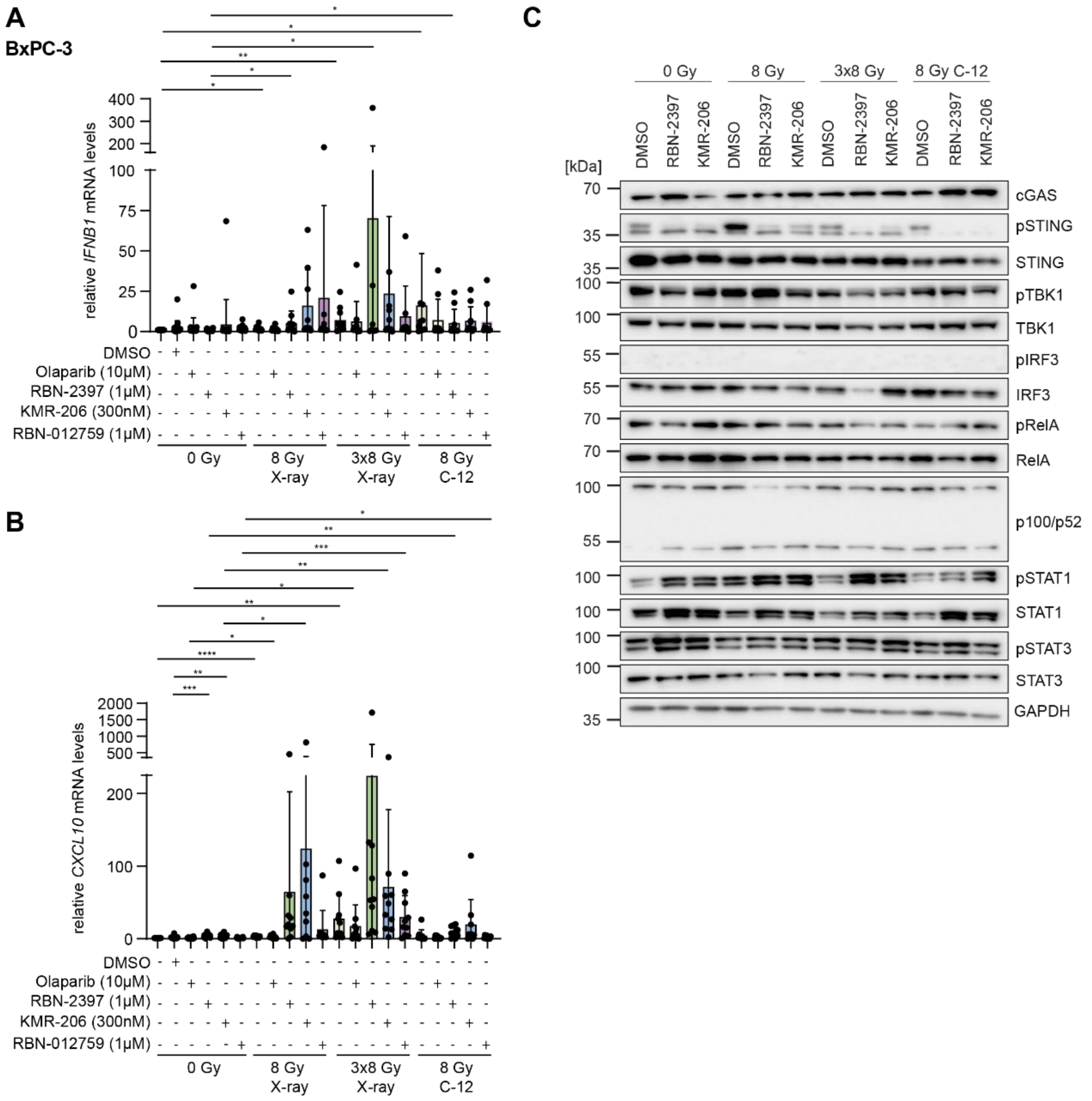

Fig. S1. PARP7 inhibitors potentiate the immunogenic effects of hypofractionated X-ray irradiation in BxPC-3 cells. (A, B) RT-qPCR analysis of the expression of *IFNB1* and *CXCL10* in BxPC-3 cells after 8 Gy or 3x8 Gy X-ray and 8 Gy C-ions without or with the PARP1/2 inhibitor olaparib, PARP7 inhibitors RBN-2397 and KMR-206 and the PARP14 inhibitor RBN-012759. Inhibitors were added 24 h before irradiation and kept until harvesting 72 h after the last fraction. Gene expression was normalized to TBP. Data points represent mean values ± standard deviation (N=3-6). Multiple t-tests were performed to determine significance (*≤ 0.05; **≤ 0.01; ***≤ 0.005, ****≤0.0001). (C) Western blot analysis of BxPC-3 cells irradiated with 8 Gy or 3x8 Gy X-ray and 8 Gy C-ions without or with PARP7 inhibitors RBN-2397 and KMR-206. GAPDH was used as a loading control.

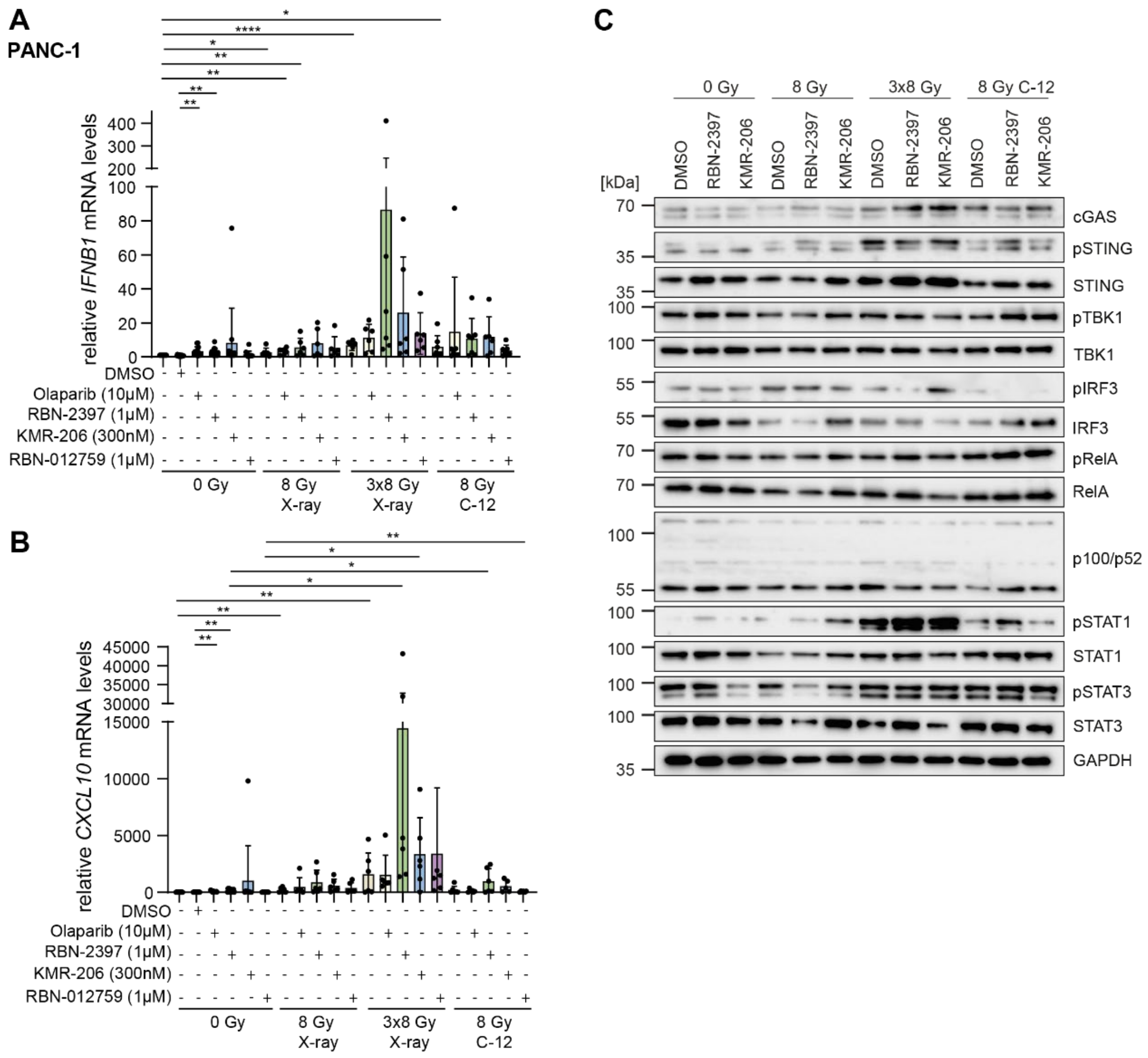

Fig. S2. PARP7 inhibitors potentiate the immunogenic effects of hypofractionated X-ray irradiation in PANC-1 cells. (A, B) RT-qPCR analysis of the expression of *IFNB1* and *CXCL10* in PANC-1 cells after 8 Gy or 3x8 Gy X-ray and 8 Gy C-ions without or with the PARP1/2 inhibitor olaparib, PARP7 inhibitors RBN-2397 and KMR-206 and the PARP14 inhibitor RBN-012759. Inhibitors were added 24 h before irradiation and kept until harvesting 72 h after the last fraction. Gene expression was normalized to TBP. Data points represent mean values ± standard deviation (N=3-6). Multiple t-tests were performed to determine significance (*≤ 0.05; **≤ 0.01; ***≤ 0.005, ****≤0.0001). (C) Western blot analysis of PANC-1 cells irradiated with 8 Gy or 3x8 Gy X-ray and 8 Gy C-ions without or with PARP7 inhibitors RBN-2397 and KMR-206.

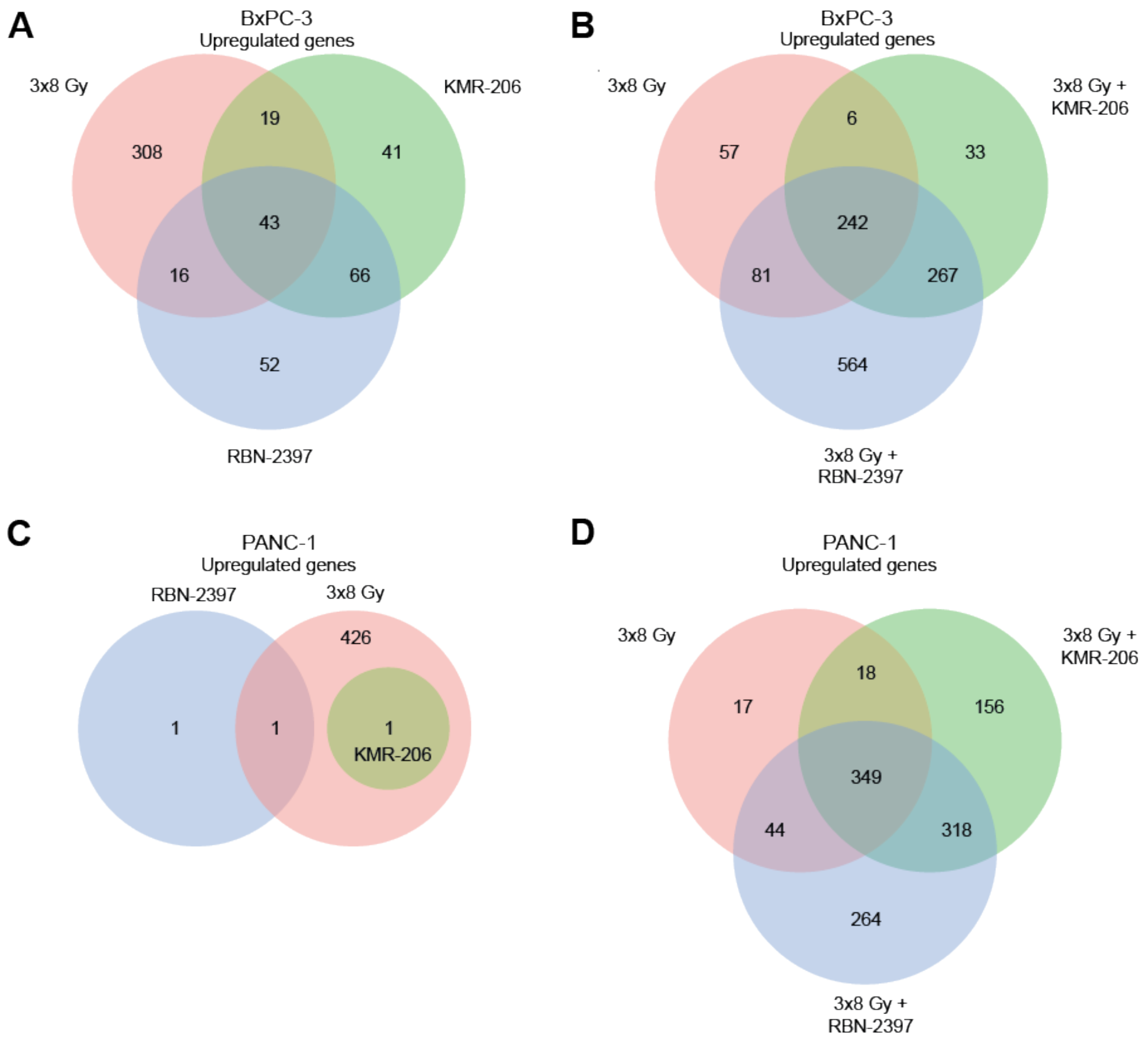

Fig. S3. Overlapping upregulated genes after radiation and PARP7i inhibitor treatments in BxPC-3 and PANC-1 cells. (A, C) Overlapping upregulated genes for 3x8 Gy, RBN-2397 and KMR-206 in (A) BxPC-3 and (C) PANC-1 cells. (B, D) Overlapping upregulated genes for 3x8 Gy, 3x8 Gy + RBN-2397 and 3x8 Gy + KMR-206 in (B) BxPC-3 and (D) PANC-1 cells.

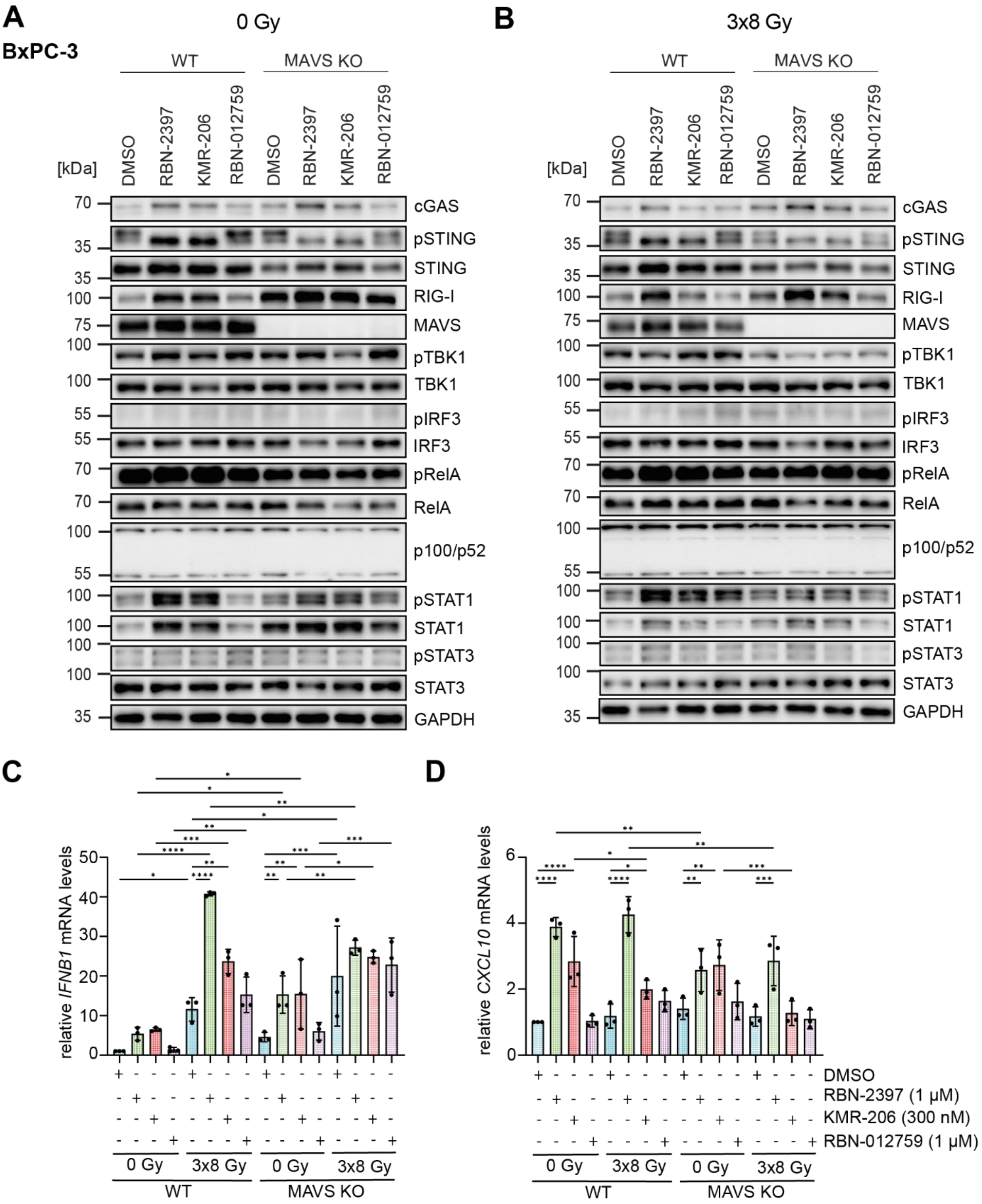

Fig. S4. The type I interferon response induced by radiation and PARP7 inhibition is partly MAVS-dependent in BxPC-3 cells. (A, B) Western blot analysis of WT and MAVS KO BxPC-3 cells treated with PARP7 and PARP14 inhibitors (A) without irradiation and (B) with 3x8 Gy X-ray. Inhibitors were added 24 h before irradiation and kept until harvesting 72 h after the last fraction. GAPDH was used as a loading control. (C, D) RT-qPCR analysis of the expression of *IFNB1* and *CXCL10* in BxPC-3 cells. Gene expression was normalized to TBP. Data points represent mean values ± standard deviation (N=3). One-way ANOVA with Tukey's post-hoc test was performed to determine significance (*≤ 0.05; **≤ 0.01; ***≤ 0.005, ****≤0.0001).

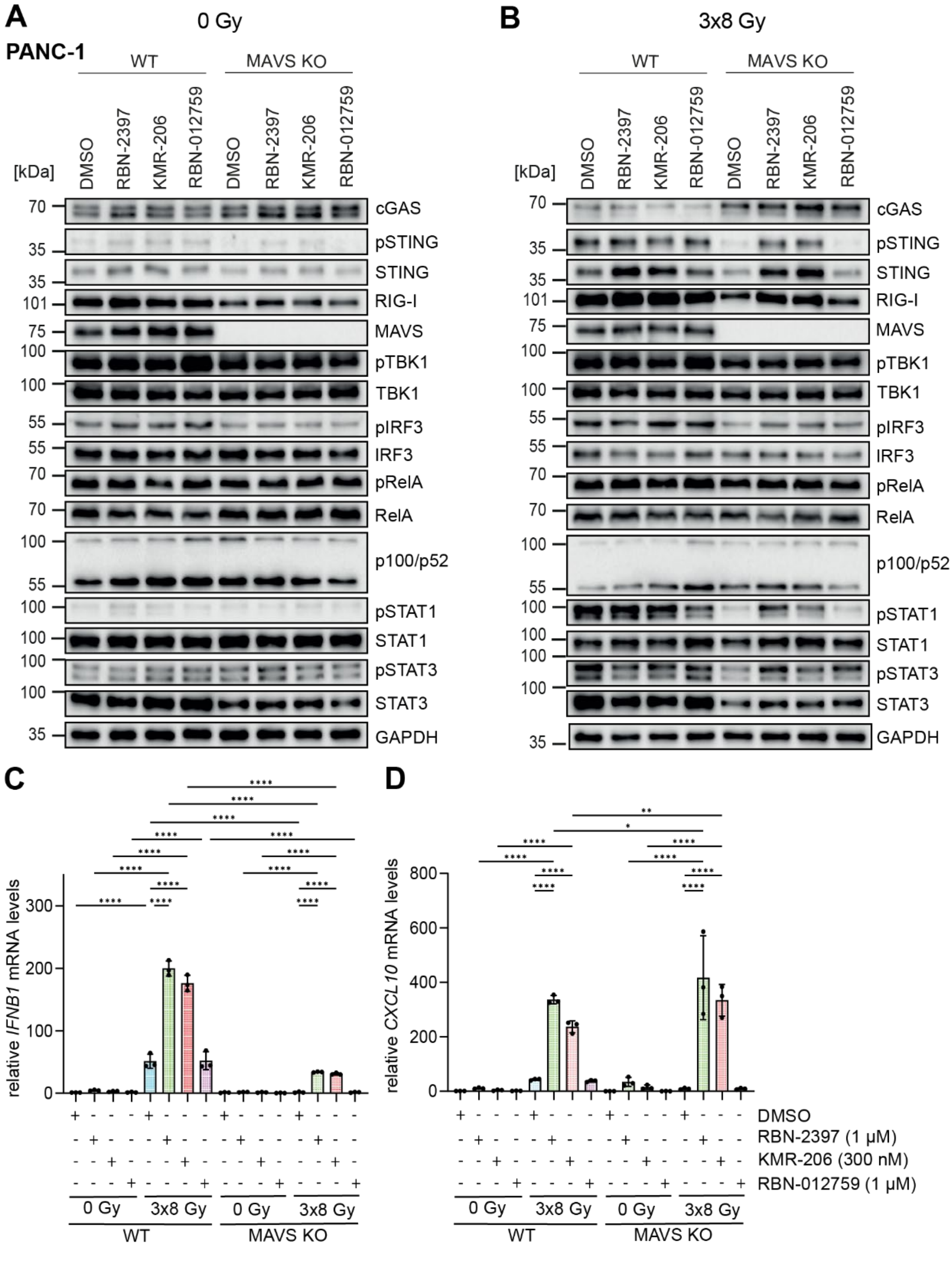

Fig. S5. The type I interferon response induced by radiation and PARP7 inhibition is partly MAVS-dependent in PANC-1 cells. (A, B) Western blot analysis of WT and MAVS KO PANC-1 cells treated with PARP7 and PARP14 inhibitors (A) without irradiation and (B) with 3x8 Gy X-ray. Inhibitors were added 24 h before irradiation and kept until harvesting 72 h after the last fraction. GAPDH was used as a loading control. (C-F) RT-qPCR analysis of the expression of *IFNB1* and *CXCL10* in PANC-1 cells. Gene expression was normalized to TBP. Data points represent mean values ± standard deviation (N=3). One-way ANOVA with Tukey's post-hoc test was performed to determine significance (*≤ 0.05; **≤ 0.01; ***≤ 0.005, ****≤0.0001).

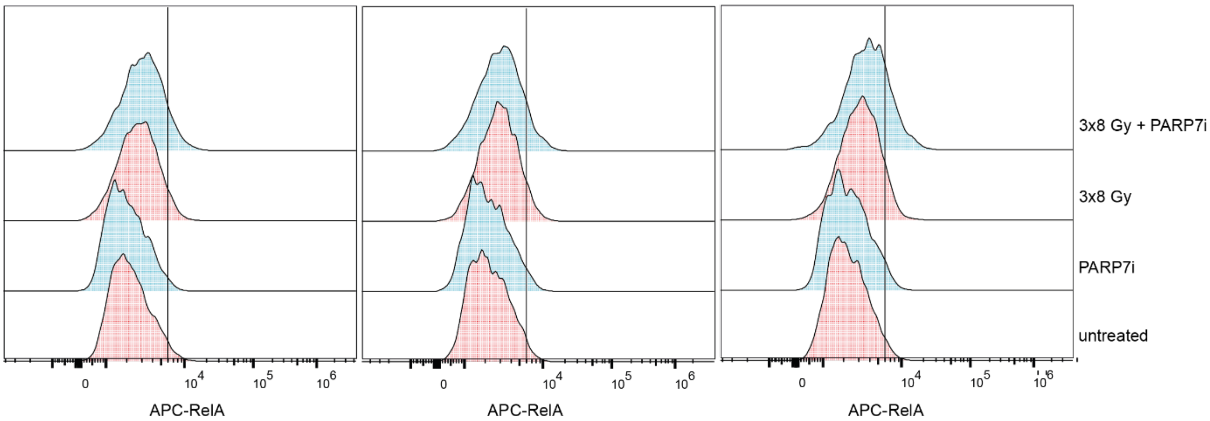

Fig. S6. Flow cytometry analysis of nuclear RelA in PANC-1. To quantify RelA nuclear translocation, nuclear proteins were immunostained with APC-RelA antibody and analyzed by flow cytometry in PANC-1 cells treated with the PARP7 inhibitor KMR-206 (300 nM), 3x8 Gy and their combination. The three sets of graphs represent three biological replicates. Cells were harvested 72 h after treatment. The solid lines represent the gate to determine the percentage of nuclear RelA-stained cells and each gate was adjusted based on the untreated sample. Flow cytometry measurements were analyzed with FlowJo_v10.8.1.

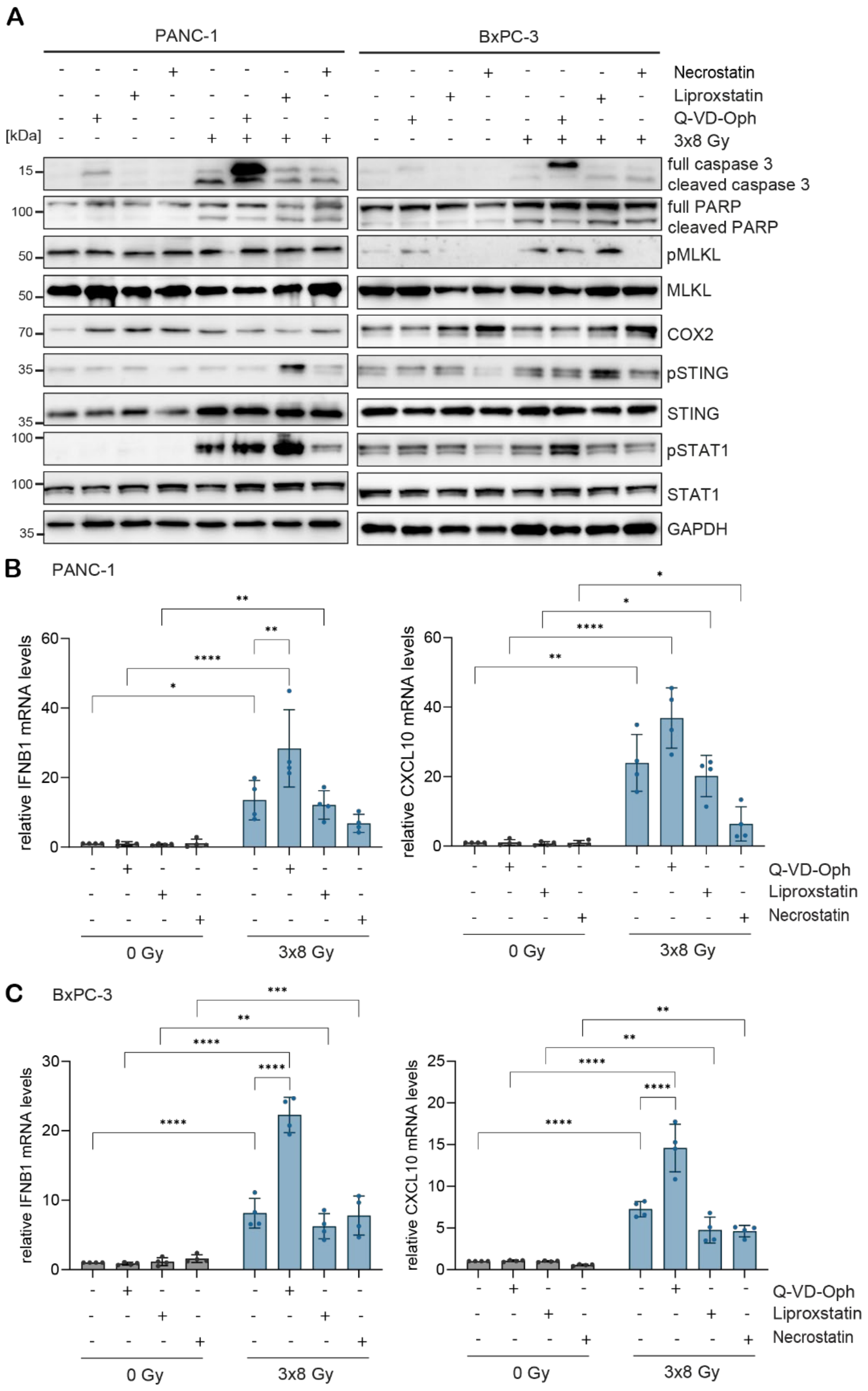

Fig. S7. Cell death pathways regulate immunogenic signaling in PDAC cells. (A) Western blot analysis of PANC-1 and BxPC-3 cells exposed to 3x8 Gy without or with ferroptosis inhibitor liproxstatin-1 (5 µM), necroptosis inhibitor necrostatin (10 µM) or apoptosis inhibitor Q-VD-Oph (10 µM). These agents were added to the cells before the first irradiation fraction and maintained until harvesting the cells 72 h after the last fraction. GAPDH was used as a loading control. (B, C) RT-qPCR analysis of the expression of *IFNB1* and *CXCL10* in (B) PANC-1 and (C) BxPC-3 cells. Gene expression was normalized to TBP. The experiments were performed in 3-4 biological replicates. Data points represent mean values ± standard deviation. One-way ANOVA with Tukey's post-hoc test was performed to determine significance (*≤ 0.05; **≤ 0.01; ***≤ 0.005, ****≤0.0001).

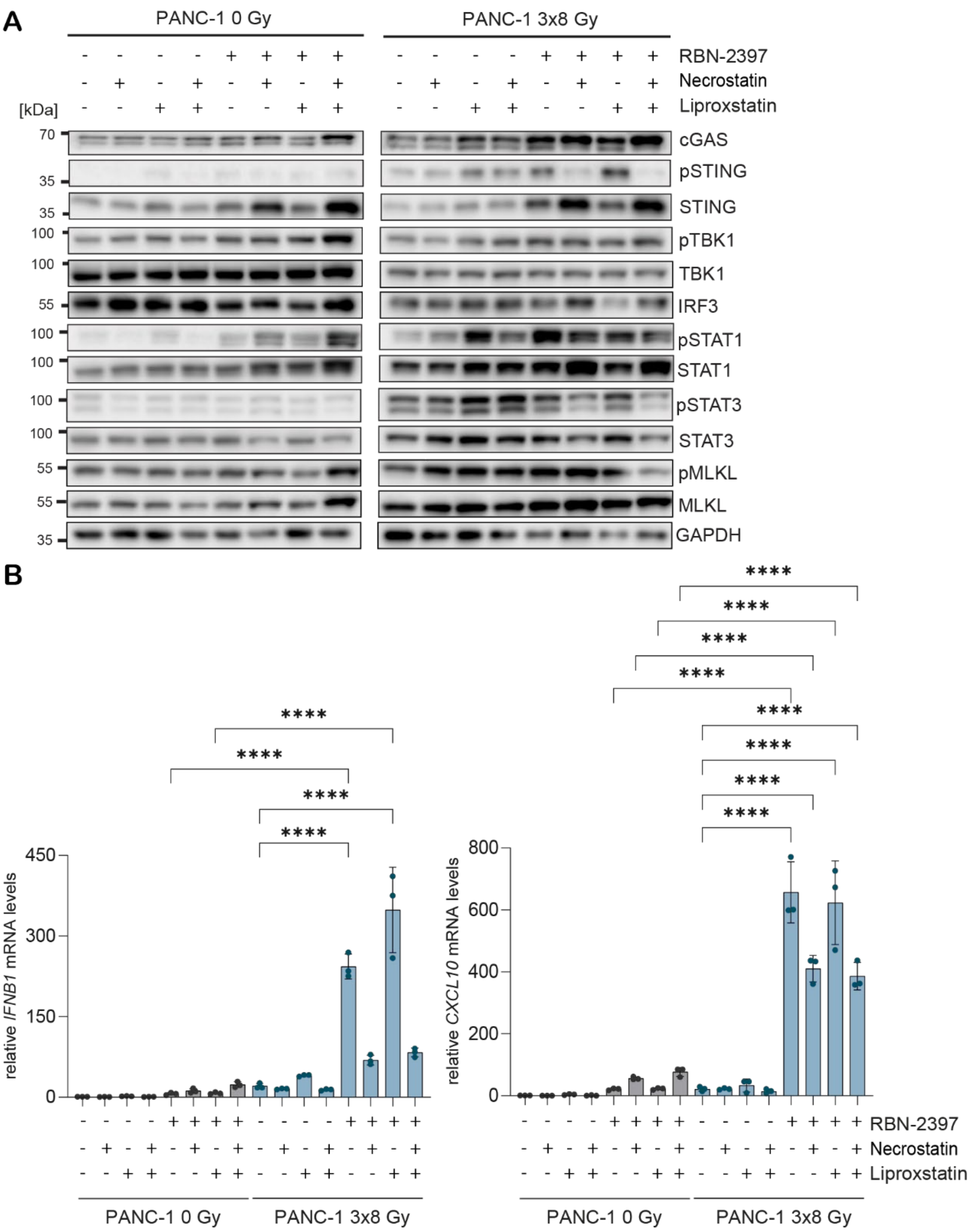

Fig. S8. Necroptosis promotes immunogenic signaling in PANC-1. (A) Western blot analysis of PANC-1 cells exposed to 3x8 Gy without or with the PARP7 inhibitor RBN-2397 (1 µM), necroptosis inhibitor necrostatin (10 µM), ferroptosis inhibitor liproxstatin-1 (5 µM), or a combination thereof. These agents were added to the cells before the first irradiation fraction and maintained until harvesting the cells 72 h after the last fraction. GAPDH was used as a loading control. (B) RT-qPCR analysis of the expression of *IFNB1* and *CXCL10*. Gene expression was normalized to TBP. The experiments were performed in three biological replicates. Data points represent mean values ± standard deviation. One-way ANOVA with Tukey's post-hoc test was performed to determine significance (*≤ 0.05; **≤ 0.01; ***≤ 0.005, ****≤0.0001).

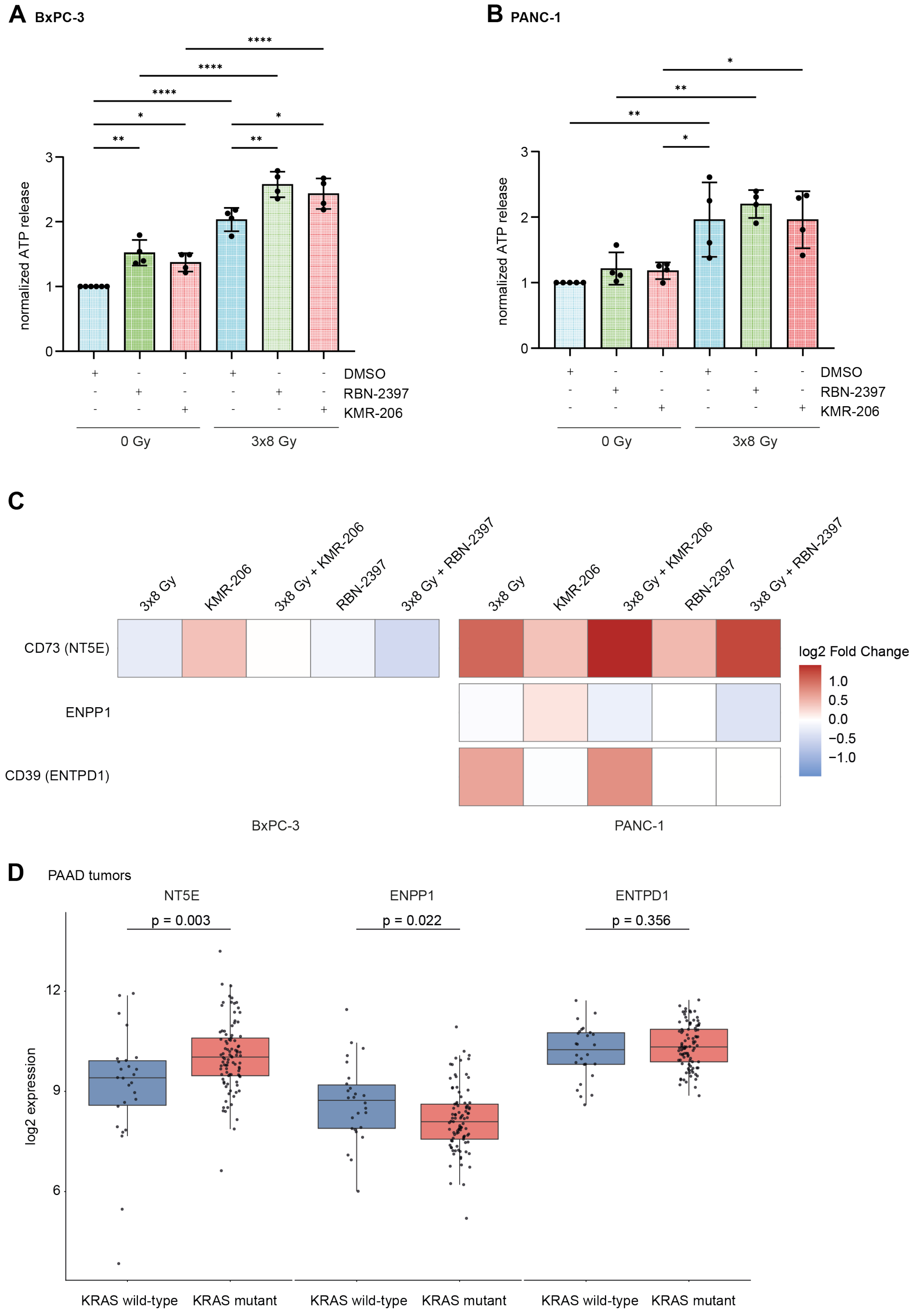

Fig. S9. Radiation induces immunogenic cell death in PDAC cells and the expression of ATP degrading enzymes in PANC-1. ATP release assay was used to measure immunogenic cell death in

(A) BxPC-3 and (B) PANC-1 cells irradiated with 3x8 Gy X-ray without or with the PARP7 inhibitors RBN-2397 (1 µM) and KMR-206 (300 nM). PARP7 inhibitors were added 24 h before irradiation and kept until the measurements 72 h after the last irradiation. The experiments were performed in four biological replicates. Data points represent mean values ± standard deviation. One-way ANOVA with Tukey's post-hoc test was performed to determine significance (*≤ 0.05; **≤ 0.01; ***≤ 0.005, ****≤0.0001). (C) Log2 fold changes in gene expression based on RNA-seq (Fig. 3) between indicated treated and control samples of genes involved in ATP degradation to adenosine. *ENPP1* and *CD39 (ENTPD1)* were not detected in BxPC-3. (D) *NT5E*, *ENPP1* and *ENTPD1* expression stratified by KRAS mutation status in TCGA PAAD tumors (KRAS wildtype n=26, KRAS mutant n=93).

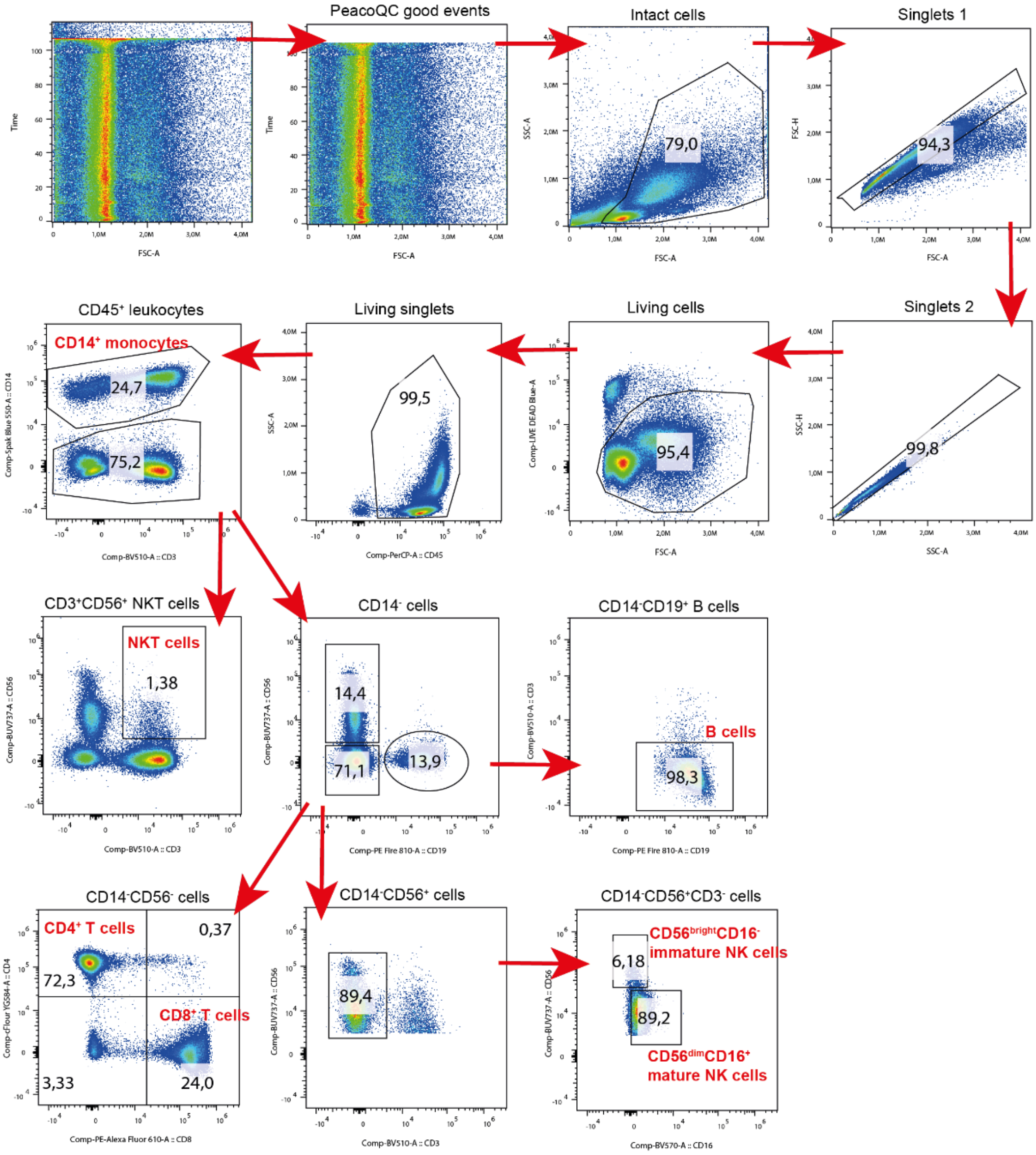

Fig. S10. Gating strategy for the identification of the immune cell subsets within peripheral blood mononuclear cells (PBMCs). Sequential gating strategy was applied to identify viable single CD45+ cells. Gating on specific lineage markers was employed to discriminate between different immune cell subsets (monocytes, NK, NKT, B cells, CD4+ and CD8+ T cells). For NK cells, two populations were identified. Only the more abundant CD56dimCD16+ mature NK cell population is discussed here and is referred to as 'NK cells'.

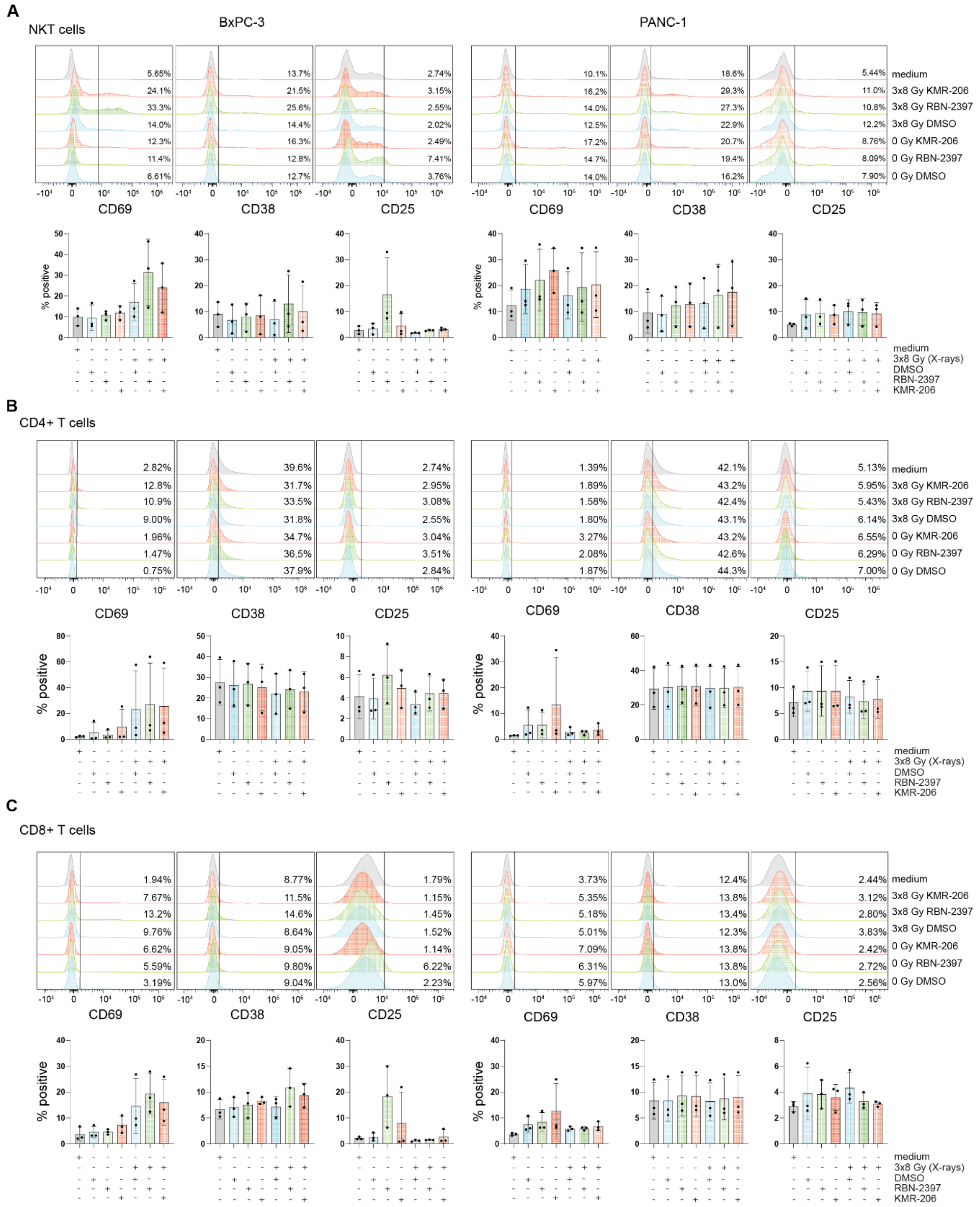

Fig. S11. Response of primary human NKT, CD4⁺ and CD8⁺ T cells to supernatants from PDAC cells treated with radiation and PARP7i. PBMCs from three different donors were incubated for 44 h in the presence of the culture medium or cell-free supernatants from BxPC-3 and PANC-1 cells treated with 3x8 Gy X-rays without or with the PARP7 inhibitors RBN-2397 (1 µM) or KMR-206 (300 nM). PARP7i were added 24 h before irradiation and kept until the measurements 72 h after the last irradiation. Human (A) NKT cells, (B) $CD4^+$ T cells and (C) $CD8^+$ T cells were identified according to lineage markers and their activation was assessed based on the analysis of activation markers CD25, CD38 and CD69 by spectral flow cytometry. Representative histograms of one donor (top) and percentages of marker-positive cells ± standard deviation of three PBMC donors (bottom) are shown. Statistical significance was assessed using one-way ANOVA with Tukey's post-hoc test (*≤ 0.05; **≤ 0.01; ***≤ 0.005, ****≤0.0001).

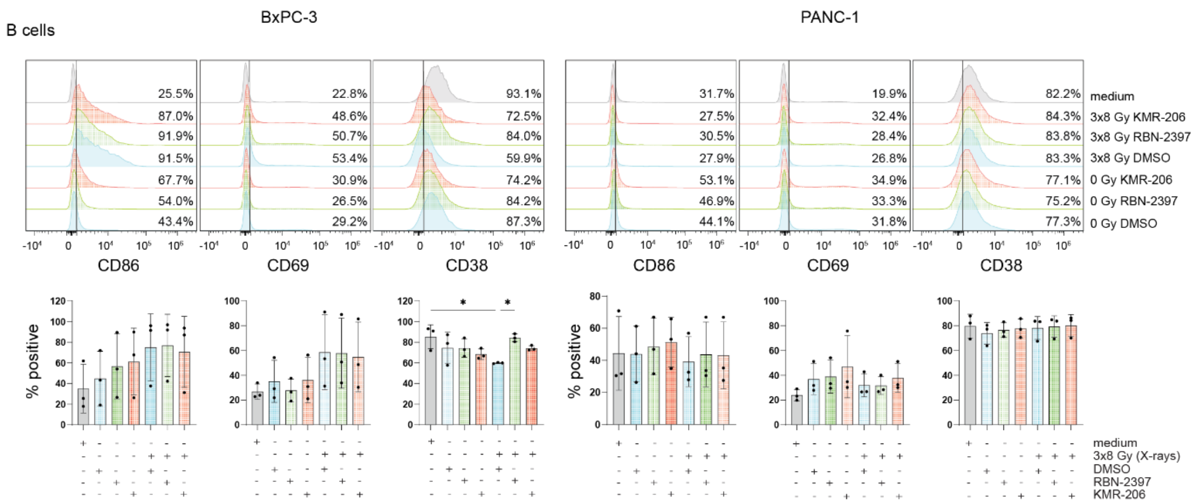

Fig. S12. Response of primary human B cells to supernatants from PDAC cells treated with radiation and PARP7i. PBMCs from three different donors were incubated for 44 h in the presence of the culture medium or cell-free supernatants from BxPC-3 and PANC-1 cells treated with 3x8 Gy X-rays without or with the PARP7 inhibitors RBN-2397 (1 µM) or KMR-206 (300 nM). PARP7i were added 24 h before irradiation and kept until the measurements 72 h after the last irradiation. Human B cells were identified according to lineage markers and their activation was assessed based on the analysis of activation markers CD86, CD69 and CD38 by spectral flow cytometry. Representative histograms of one donor (top) and percentages of marker-positive cells ± standard deviation of three PBMC donors (bottom) are shown. Statistical significance was assessed using one-way ANOVA with Tukey's post-hoc test (*≤ 0.05; **≤ 0.01; ***≤ 0.005, ****≤0.0001).

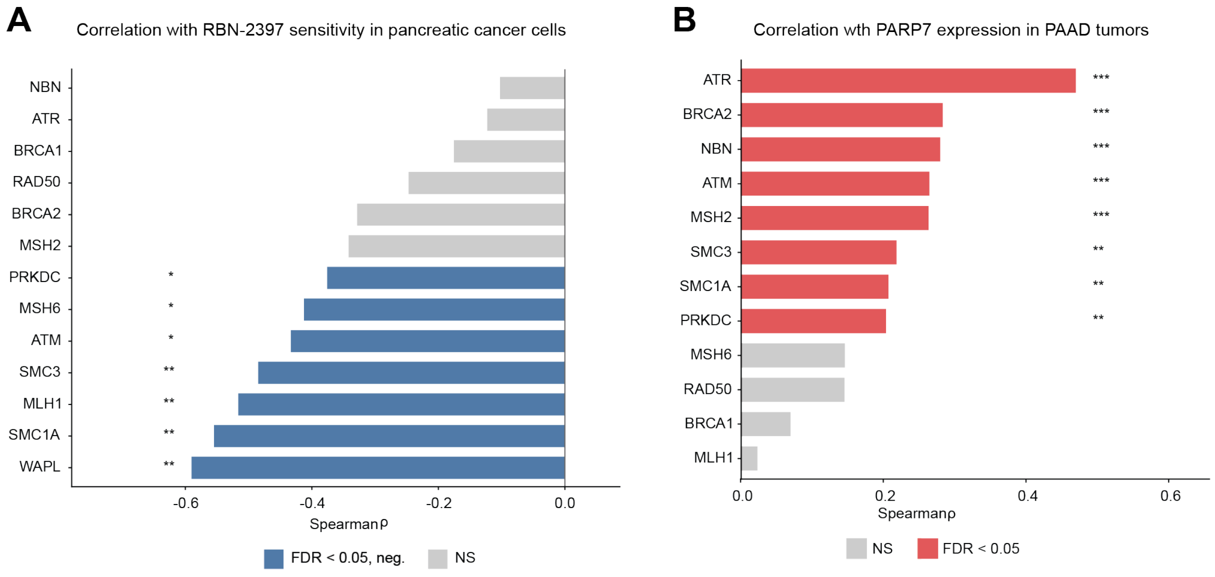

Fig. S13. PARP7 expression in pancreatic tumors correlates with genome integrity pathways. (A) Spearman correlations between expression of selected genes and RBN-2397 sensitivity in 37 pancreatic cancer cell lines. ** FDR<0.01, *** FDR<0.001. (B) Spearman correlations between PARP7 and selected genes expression in 178 TCGA PAAD tumor samples. ** FDR<0.01, *** FDR<0.001.

Table S1. RNA-seq analysis of BxPC-3 and PANC-1 cells before and after treatment with 3x8 Gy without or with the PARP7 inhibitors RBN-2397 (1 μM) and KMR-206 (300 nM). These agents were added to the cells 2 h before the first radiation fraction and maintained until harvesting the cells 72 h after the last fraction.

Table S2. RNA-seq analysis of repetitive elements in BxPC-3 and PANC-1 cells before and after treatment with 3x8 Gy without or with the PARP7 inhibitors RBN-2397 (1 μM) and KMR-206 (300 nM). These agents were added to the cells 2 h before the first radiation fraction and maintained until harvesting the cells 72 h after the last fraction.

Table S3. Immunophenotyping panel used in spectral flow cytometry analysis of PBMCs. mAbs used in both the full immunophenotyping and the lineage panel are depicted in bold. DC – dendritic cell, Treg – regulatory T cell.

**Marker**	**Fluorochrome**	**Clone**	**Company**	**Cat. Number**	**Final dilution**	**Purpose**
CD45RA	BUV395	5H9	BD Biosciences	750631	1:460	T cell and DC differentiation
**Viability**	**LIVE DEAD Blue**	**-**	**Thermo Fisher**	**L23105**	**1:500 (prestain)**	**Viability**
**CD56**	**BUV737**	**NCAM16.2**	**BD Biosciences**	**612766**	**1:70**	**NK cells**
CD195/CCR5	BUV805	3A9	BD Biosciences	748872	1:70	Chemokine receptor; monocyte, DC, T cell, B cell differentiation
CD197/CCR7	BV421	G043H7	BioLegend	353208	1:46	T cell differentiation
**CD3**	**BV510**	**SK7**	**BioLegend**	**344828**	**1:175**	**T cells, NKT cells**
**CD16**	**BV570**	**3G8**	**BioLegend**	**302036**	**1:140**	**Monocyte, NK cell, and DC differentiation**
CD279/PD-1	BV785	EH12.2H7	BioLegend	329930	1:140	T cell inhibitory receptor
**CD14**	**Spark Blue 550**	**63D3**	**BioLegend**	**367148**	**1:280**	**Monocyte differentiation**
**CD45**	**PerCP**	**2D1**	**BioLegend**	**368506**	**1:100**	**Leukocytes**
CD11b	PerCP-Cy5.5	LM2	BioLegend	393106	1:46	DC differentiation, monocyte activation
**TCR γδ**	**PerCP-eFluor710**	**B1.1**	**Thermo Fisher**	**46-9959-42**	**1:280**	**γδ T cells**
**CD4**	**cFluor YG584**	**SK3**	**CYTEK**	**R7-20041**	**1:116**	**CD4 T cells, NKT cells**
**CD8**	**PE-Alexa Flour 610**	**3B5**	**Thermo Fisher**	**MHCD0822**	**1:460**	CD8 T cells, NK cells and NKT cells

CD80	PE-Cy5	L307.4	BD Biosciences	559370	1:70	Monocyte, DC and B cell activation marker
CD25/IL2Rα	PE-Alexa Flour 700	CD25-3G10	Thermo Fisher	MHCD2524	1:140	Treg marker, activation marker on T cells, B cells, myeloid cells
CD183/CXCR3	PE-Vio770	CEW33D	Thermo Fisher	25-1839-42	1:200	Chemokine receptor; DC, T, and B cell differentiation
**CD19**	**PE-Fire 810**	**HIB19**	**BioLegend**	**302287**	**1:280**	**B cells**
CD86	APC	BU63	BioLegend	374208	1:280	Monocyte, DC and B cell activation marker
CD69	APC-R700	FN50	BD Biosciences	565154	1:93	Early activation marker expressed on leukocytes
HLA-DR	APC-Fire 750	L243	BioLegend	307657	1:175	T cell and monocyte activation, DC lineage marker
CD38	APC-Fire 810	HIT2	BioLegend	303549	1:200	Monocyte, DC, T and B cell activation/differentiation

Table S4. Antibodies

Rabbit anti-β-Actin	Cell Signaling	RRID: AB_2223172; 4970S; 1:1000 for WB
Rabbit anti-GAPDH	Cell Signaling	RRID: AB_10622025; 5174S; 1:1000 for WB
Rabbit anti-cGAS	Cell Signaling	RRID: AB_2732795; 15102S; 1:1000 for WB
Rabbit anti-cGAS	Proteintech	26416-1-AP; IF 1:200
Rabbit anti-pSTING	Cell Signaling	RRID: AB_2737062; 19781S; 1:1000 for WB
Rabbit anti-STING	Cell Signaling	RRID: AB_2732796; 13647S; 1:1000 for WB
Rabbit anti-RIG-I	Cell Signaling	RRID: AB_2269233; 3743S; 1:1000 for WB

Rabbit anti MAVS	Cell Signaling	RRID: AB_2798889; 24930S; 1:1000 for WB
Rabbit anti-pTBK1	Cell Signaling	RRID: AB_10693472; 5483S; 1:1000 for WB; 1:400 for IF
Rabbit anti-TBK1	Cell Signaling	RRID: AB_2827657; 38066S; 1:1000 for WB
Rabbit anti-pIRF3	Cell Signaling	RRID: AB_823547; 4947S; 1:1000 for WB
Rabbit anti-IRF3	Cell Signaling	RRID: AB_1904036; 4302S; 1:1000 for WB
Mouse anti-IkBa	Cell Signaling	RRID: AB_2084807; 4814; 1:1000 for WB
Rabbit anti-pRelA	Cell Signaling	RRID: AB_331284; 3033; 1:1000 for WB
Rabbit anti-RelA	Proteintech	RRID: AB_2178878; 10745-1-AP; 1:1000 for WB
Rabbit anti-p100/52	Cell Signaling	RRID: AB_10695537; 4882; 1:1000 for WB
Rabbit anti-pSTAT1	Cell Signaling	RRID: AB_561284; 9167S; 1:1000 for WB
Rabbit anti-STAT1	Cell Signaling	RRID: AB_2737027; 14994S; 1:1000 for WB
Rabbit anti-pSTAT3	Cell Signaling	RRID: AB_2491009; 9145; 1:1000 for WB
Mouse anti-STAT3	Abcam	RRID: AB_10901752; Ab119352; 1:1000 for WB
Rabbit anti-PARP1 / cleaved PARP1	Cell Signaling	RRID: AB_2160739; 9542; 1:1000 for WB
Rabbit cleaved caspase 3	Cell Signaling	RRID: RRID:AB_2070042; 9664; 1:1000 for WB
Rabbit anti-pMLKL	Cell Signaling	RRID: AB_2895044; 91689; 1:1000 for WB

Rabbit anti-MLKL	Cell Signaling	RRID: AB_2721822; 14993; 1:1000 for WB
Rabbit anti-COX2	Cell Signaling	RRID: AB_2571729; 12282; 1:1000 for WB
Rabbit anti-GPX4	Cell Signaling	RRID: AB_2924984; 52455; 1:1000 for WB
Goat anti-rabbit-IgG-HRP	Jackson ImmunoResearch	RRID: AB_2337937; 111-035-008; 1:10000 for WB
Goat anti-mouse-IgG-HRP	Jackson ImmunoResearch	RRID: AB_2313585; 115-035-008; 1:10000 for WB

Table S5. Raw data with uncropped western blot replicates.
